# Supplementary material for: Associations between preoperative Oxford hip and knee scores and costs and quality of life of patients undergoing primary total joint replacement in the NHS England: an observational study
Source: BMJ Open. 2018 Apr 10;8(4):e019477. doi: 10.1136/bmjopen-2017-019477 (PMC5898298; doi:10.1136/bmjopen-2017-019477)
Supplement: Supplementary data [file bmjopen-2017-019477supp001.pdf]

Appendix

A. Diagnoses and procedures codes used to define hospital readmissions

We used ICD-10 diagnosis codes and OPCS 4 procedure codes to determine whether a hospital admission was related to arthroplasty. Specifically, we used the following criteria:

- A hospital admission occurred within 30 days after the primary arthroplasty episode. This criterion was applied regardless of any specific diagnosis or procedure codes.
- The primary diagnosis (i.e., the first recorded ICD-10 diagnosis code) was M17.X (knee arthritis) for a knee replacement episode, or M16.X (hip arthritis) for a hip replacement episode.
- One of the recorded procedure codes referred to the hip or knee joint. The OPCS 4 codes Z843, Z761 or Z756 were used for hip replacement episodes, and the codes Z846, Z765, Z845. Z844, Z774 or Z787 were used for knee replacement episodes.
- The admission had a diagnosis code associated with infections of the skin, the joint or the prosthesis. We used the following ICD-10 codes for this purpose:
    - M25.X with Z96.6 – "Other joint disorder, not elsewhere classified" and "Presence of orthopedic joint implant"
    - T81.X with either Z96.6 or Y83.1 – "Complications of procedures, not elsewhere classified" and "Presence of orthopedic joint implant" or "Surgical operation with implant of artificial internal device […]"
    - T84.X – "Complications of internal orthopedic prosthetic devices, implants and grafts"
    - M96.X – "Intraoperative and postprocedural complications and disorders of musculoskeletal system, not elsewhere classified"
    - M00.X with Z96.6 – "Pyogenic arthritis" and "Presence of orthopedic joint implant"
    - M86.X with Z96.6 – "Osteomyelitis" and "Presence of orthopedic joint implant".

B. Unit costs

Table B.1 Unit costs applied to all primary datasets providing resource use data

Healthcare resource	Units	Unit cost	Reference and details
GP surgery visit	Per visit	£46	PSSRU 2014 page 195[1]: Cost of a surgery patient contact lasting 11.7 minutes was £46 including direct care staff costs and qualifications. A clinic patient contact lasting 17.2 minutes costs £67 including direct care staff costs and qualifications.
GP Practice nurse visit	Per visit	£13.69	PSSRU 2014 page 192: £53 per hour of face-to-face contact including qualifications (£44/hour excluding qualifications). Average surgery consultation for a GP practice nurse lasts 15.5 minutes, giving a consultation cost of £14
Community physiotherapist at clinic/GP surgery	Per visit	£51	NHS reference costs – PSSRU 2014 page 179 states that the mean cost for a one-to-one contact in physiotherapy services in 2013/2014 was £51. PSSRU estimates the unit cost per hour in 2013/2014 to be £32 without qualifications or £36 with qualifications (page 179)
Community physiotherapist – at home	Per visit	£51	For simplicity, applied the same cost as physiotherapy at GP surgery/clinic
Hospital physiotherapist	Per visit	£34	NHS reference costs – PSSRU 2014 page 235 states that the mean average cost for a non-consultant led (not admitted) follow-up physiotherapy attendance in 2013/14 was £34 (IQR £28-£38). PSSRU 2014 estimates the hourly cost of a hospital physiotherapist to be £37/hour including qualifications and £33 excluding qualifications
Physiotherapy – average	Per visit	£42.50	We assumed that 50% of physiotherapy visits are in hospital and 0% are in the community.

Healthcare resource	Units	Unit cost	Reference and details
Outpatient visit – orthopaedics – Follow-up visit	Per visit	£71	Payment by results tariff [2] Tariff information spreadsheet 2013-14: outpatient attendances- WF01A Follow Up Attendance - Single Professional Or WF02A Follow Up Attendance - Multi-Professional (both of these codes are the same price). Follow-up attendance is used here, since patients are assumed to have already attended clinic for their joint replacement
A&E attendance – not admitted	Per visit	£81.26	National schedule of reference costs [3] weighted average of all non-admitted accident and emergency attendances , weighted by the number of attendances.
Admission to hospital related to hip arthroplasty (No data on length of stay)	Per admission	£2,662	Department of Health reference costs 2013-14. [3] This was calculated by taking a weighted average cost of all HRGs with "hip" within the currency description on the EL & NEL worksheets, weighting by the number of FCEs.
Admission to hospital related to knee arthroplasty (No data on length of stay)	Per admission	£3,907	Department of Health reference costs 2013-14 [3]. This was calculated by taking a weighted average cost of all HRGs with "knee" within the currency description on the EL & NEL worksheets, weighting by the number of FCEs.
Readmission to hospital within 30 days of hip or knee arthroplasty	Per bed-day	£395.83	Average costs per bed-day for readmissions in PROMs/HES within 30 days of hip or knee arthroplasty
Readmission to hospital within 30 days of hip or knee arthroplasty (No data on length of stay)	Per admission	£1,255	Average costs for readmissions in PROMs/HES within 30 days of hip or knee arthroplasty
Readmission to hospital with a diagnosis of hip/knee arthritis after hip/knee arthroplasty	Per bed-day	£1,107.25	Average costs per bed-day for readmissions in PROMs/HES with a diagnosis of hip/knee arthritis after hip/knee arthroplasty
Readmission to hospital with a diagnosis of	Per	£5,220	Average costs for readmissions in PROMs/HES with a diagnosis of

Healthcare resource	Units	Unit cost	Reference and details
hip/knee arthritis after hip/knee arthroplasty (No data on length of stay)	admission		hip/knee arthritis after hip/knee arthroplasty
Readmission to hospital for a hip/knee-specific procedure following hip/knee arthroplasty	Per bed-day	£551.33	Average costs per bed-day for readmissions in PROMs/HES for a hip/knee-specific procedure following hip/knee arthroplasty
Readmission to hospital for a hip/knee-specific procedure following hip/knee arthroplasty (No data on length of stay)	Per admission	£1,654	Average costs for readmissions in PROMs/HES for a hip/knee-specific procedure following hip/knee arthroplasty
Readmission to hospital with a diagnosis for infection following hip/knee arthroplasty	Per bed-day	£694.8	Average costs per bed-day for readmissions in PROMs/HES with a diagnosis for infection following hip/knee arthroplasty
Readmission to hospital with a diagnosis for infection following hip/knee arthroplasty (No data on length of stay)	Per admission	£1,876	Average costs for readmissions in PROMs/HES diagnosis for infection following hip/knee arthroplasty

References

[1] PSSRU | Unit Costs of Health and Social Care 2014 n.d. http://www.pssru.ac.uk/project-pages/unit-costs/2014/ (accessed June 14, 2017).
[2] Department of Health. Payment by Results in the NHS: tariff for 2014 to 2015: 2014- 15 tariff information spreadsheet. 2014.
[3] Department of Health. NHS reference costs 2013 to 2014 2014. https://www.gov.uk/government/publications/nhs-reference-costs-2013-to-2014 (accessed June 14, 2017).

C. Regression results

Table C.1: Associations between pre-operative OHS/OKS and post-operative EQ-5D-3L

	**Hip arthroplasty**			**Knee arthroplasty**		
Model	*Tobit model censored at -0.594 and 1*			*Tobit model censored at -0.594 and 1*		
**Variable**	**Coefficient**		**Robust S.E.**	**Coefficient**		**Robust S.E.**
			OHS/OKS			
Linear term	5.839E-02	***	4.337E-03	5.725E-02	***	2.482E-03
Quadratic term	-3.312E-03	***	5.400E-04	-2.356E-03	***	2.008E-04
Cubic term	1.182E-04	***	2.990E-05	5.390E-05	***	6.600E-06
Quartic term	-2.110E-06	**	7.520E-07	-4.660E-07	***	7.530E-08
Quintic term	1.460E-08	*	6.980E-09	-	-	-
			Age (reference category: 50-54)			
Age <30	-9.039E-02	***	1.717E-02	-1.467E-01	*	6.181E-02
Age 30-39	-4.423E-02	***	1.047E-02	-4.303E-02		2.371E-02
Age 40-49	-1.240E-02	*	6.128E-03	-2.316E-02	**	6.787E-03
Age 55-59	3.204E-03		5.012E-03	1.138E-02	*	4.470E-03
Age 60-64	2.120E-02	***	4.592E-03	4.654E-02	***	4.094E-03
Age 65-69	3.235E-02	***	4.422E-03	8.938E-02	***	3.980E-03
Age 70-74	3.831E-03		4.377E-03	8.991E-02	***	3.966E-03
Age 75-79	-2.073E-02	***	4.400E-03	8.561E-02	***	4.000E-03
Age 80-84	-4.607E-02	***	4.629E-03	8.467E-02	***	4.208E-03
Age 85-89	-6.786E-02	***	5.558E-03	7.706E-02	***	5.099E-03
Age 90-94	-6.984E-02	***	1.027E-02	7.987E-02	***	1.066E-02
Age >=95	-1.072E-01	**	4.130E-02	9.356E-02		4.858E-02
Female sex	-3.099E-02	***	1.691E-03	-	-	-
Constant term	4.451E-01	***	1.297E-02	1.741E-01	***	1.105E-02
σ (Tobit term)	3.442E-01	-	8.904E-04	2.997E-01	-	7.109E-04
N	208,345			223,836		
Data	PROMs-HES			PROMs-HES		
			Candidate models			
Model class	OLS, Tobit, two-part (Logit-OLS)			OLS, Tobit, two-part (Logit-OLS)		
OHS/OKS trend	Quadratic, cubic, quartic, quintic, log-linear			Quadratic, cubic, quartic, quintic, log-linear		
Age trend	Linear, quadratic, cubic, quartic, quintic, five-year bands, exclusion			Linear, quadratic, cubic, quartic, quintic, linear spline (65), five-year bands, exclusion		
Sex	yes/no			yes/no		

Table C.2: Associations between pre-operative OHS/OKS and EQ-5D-3L before TJR

	Hip arthroplasty			Knee arthroplasty		
Model	*Tobit model censored at -0.594 and 1*			*Tobit model censored at -0.594 and 1*		
**Variable**	**Coefficient**		**Robust S.E.**	**Coefficient**		**Robust S.E.**
			OHS/OKS			
Linear term	5.719E-03	**	2.040E-03	1.001E-02	***	2.403E-03
Quadratic term	2.385E-03	***	2.641E-04	2.150E-03	***	2.997E-04
Cubic term	-3.800E-05	*	1.500E-05	-3.140E-05		1.660E-05
Quartic term	-1.480E-06	***	3.850E-07	-1.650E-06	***	4.190E-07
Quintic term	3.010E-08	***	3.650E-09	3.240E-08	***	3.920E-09
			Age			
Linear term	-8.742E-03	***	3.866E-03	-2.943E-02	**	1.046E-02
Quadratic term	3.742E-04		1.062E-04	7.750E-04	**	2.541E-04
Cubic term	-5.190E-06	***	1.240E-06	-8.360E-06	**	2.700E-06
Quartic term	2.340E-08		5.290E-09	3.270E-08	**	1.060E-08
Female sex	-	-	-	1.040E-02	***	8.351E-04
Constant term	-1.649E-01	**	5.079E-02	1.791E-01		1.592E-01
σ (Tobit term)	2.128E-01	-	3.022E-04	2.177E-01	-	2.895E-04
N	271,045			290,983		
Data	PROMs-HES			PROMs-HES		
			Candidate models			
Model class	OLS, Tobit, two-part (Logit-OLS)			OLS, Tobit, two-part (Logit-OLS)		
OHS/OKS trend	Quadratic, cubic, quartic, quintic, log-linear			Quadratic, cubic, quartic, quintic, log-linear		
Age trend	Linear, quadratic, cubic, quartic, quintic, five-year bands, exclusion			Linear, quadratic, cubic, quartic, quintic, five-year bands, exclusion		
Sex	yes/no			yes/no		

Sources: NHS PROMs and HES data, 2009-2015; own calculations.* p<0.05; ** p<0.01; *** p<0.001. Details on how to calculate predictions are provided in Appendix D.

Table C.3: Associations between pre-operative OKS and EQ-5D-3L between 1 and 12 years after TKR

	Knee arthroplasty		
Model	*OLS*		
**Variable**	**Coefficient**		**Clustered S.E.**
	OKS		
Log-linear term	1.776E-01	***	1.111E-02
	Current age		
Linear term	7.168E-03	***	1.541E-03
Linear spline term (70)	-8.851E-03	**	2.551E-03
Year since operation	-6.154E-03	***	1.417E-03
Interaction between year and linear spline term	-3.729E-04	*	1.669E-04
Previously revised	-1.598E-01	***	3.239E-02
Constant term	-2.372E-01	*	1.031E-01
N	15,414 (2,004 patients)		
Data	KAT		
	Candidate models		
Model class	OLS with clustered standard errors, linear mixed models with random intercept and fixed slopes		
Time trend	Linear, quadratic		
OKS trend	Linear, quadratic, cubic, log-linear		
Age trend	For both age at operation and current age: Linear, quadratic, cubic, linear spline (70), exclusion		
Sex	yes/no		

Sources: KAT data; own calculations.* p<0.05; ** p<0.01; *** p<0.001. Details on how to calculate predictions are provided in Appendix D. The model was estimated on data in long-format (i.e., one observation per patient and year). The inclusion of the time trend was tested after selecting the model class and before choosing a trend for OKS. The linear spline term equals "(current age - 70)" for patients above 70, and "0" for those below. The interaction term is derived by multiplying the linear spline term with the year variable. We did not estimate a model for hips due to a lack of data.

Table C.4: Associations between pre-operative OHS/OKS and EQ-5D-3L before revision surgery

	Hip arthroplasty			Knee arthroplasty		
Model	*Tobit model censored at -0.594 and 1*			*Tobit model censored at -0.594 and 1*		
**Variable**	**Coefficient**		**Clustered S.E.**	**Coefficient**		**Clustered S.E.**
OHS/OKS						
Linear term	2.665E-02	***	4.188E-03	2.631E-02	**	8.271E-03
Quadratic term	-2.867E-04	**	1.041E-04	2.356E-04		4.623E-04
Cubic term	-	-	-	-1.510E-05		7.740E-06
Age at primary operation						
Linear term	-	-	-	4.472E-03	***	9.990E-04
Linear spline term (70)	-	-	-	-5.607E-03		2.879E-03
Age at revision surgery						
Linear term	2.123E-03	**	7.587E-04	-	-	-
Logarithm of year of revision	-	-	-	-3.601E-02		1.882E-02
Male sex	-	-	-	-2.358E-02		1.368E-02
Constant term	-1.444E-01	*	6.087E-02	-3.498E-01	***	7.534E-02
σ (Tobit term)	3.178E-01	-	4.755E-03	2.999E-01	-	3.676E-03
N	1,391 (1,331 patients)			2,227 (2,073 patients)		
Data	PROMs-HES			PROMs-HES		
Candidate models						
Model class	OLS, Tobit			OLS, Tobit		
Year of revision (time trend)	Linear, exclusion			Linear, quadratic, cubic, log-linear, exclusion		
OHS/OKS trend	Linear, quadratic, cubic, log-linear			Linear, quadratic, cubic, quartic, log-linear, linear spline (30)		
Age trend	Age at operation vs. age at revision surgery; for age at revision surgery: linear, quadratic, cubic, exclusion			Age at operation vs. age at revision surgery; for age at operation: linear, quadratic, cubic, linear spline (70), exclusion		
Sex	yes/no			yes/no		

Sources: NHS PROMs and HES data, 2009-2015; own calculations.* p<0.05; ** p<0.01; *** p<0.001. Details on how to calculate predictions are provided in Appendix D. The linear spline term equals "(age at operation - 70)" for patients above 70, and "0" for those below.

Table C.5: Associations between pre-operative OHS/OKS and EQ-5D-3L after revision surgery

	Hip arthroplasty			Knee arthroplasty		
Model	*Tobit model censored at -0.594 and 1*			*Tobit model censored at -0.594 and 1*		
**Variable**	**Coefficient**		**Clustered S.E.**	**Coefficient**		**Clustered S.E.**
	OHS/OKS					
Log-linear term	2.201E-01	***	2.580E-02	2.556E-01	***	1.950E-02
	Age at revision surgery					
Linear term	-8.181E-02		6.954E-02	1.352E-02	***	2.570E-03
Quadratic term	1.755E-03		1.099E-03	-	-	-
Cubic term	-1.120E-05		5.680E-06	-	-	-
Linear spline term (65)	-	-	-	-1.533E-02	***	3.747E-03
Male sex	-5.907E-02	*	2.696E-02	-8.874E-02	***	1.982E-02
Constant term	1.120E+00		1.445E+00	-9.426E-01	***	1.623E-01
σ (Tobit term)	3.633E-01	-	1.184E-02	3.441E-01	-	7.576E-03
N	884 (860 patients)			1,398 (1,331 patients)		
Data	PROMs-HES			PROMs-HES		
	Candidate models					
Model class	OLS, Tobit, two-part (Logit-OLS)			OLS, Tobit, two-part (Logit-OLS)		
Year of revision (time trend)	Linear, exclusion			Linear, quadratic, cubic, log-linear, exclusion		
OHS/OKS trend	Linear, quadratic, cubic, log-linear			Linear, quadratic, cubic, quartic, log-linear		
Age trend	Age at operation vs. age at revision surgery; for age at revision surgery: linear, quadratic, cubic, exclusion			Age at operation vs. age at revision surgery; for age at revision surgery: linear, quadratic, cubic, linear spline (65), exclusion		
Sex	yes/no			yes/no		

Sources: NHS PROMs and HES data, 2009-2015; own calculations.* p<0.05; ** p<0.01; *** p<0.001. Details on how to calculate predictions are provided in Appendix D. The linear spline term equals "(age at revision surgery - 65)" for patients above 65, and "0" for those below.

Table C.6: Associations between pre-operative OHS/OKS and the costs of primary arthroplasty

	Hip arthroplasty			Knee arthroplasty		
Model	*gamma-GLM with inverse link function*			*gamma-GLM with inverse link function*		
**Variable**	**Coefficient**		**S.E.**	**Coefficient**		**SE**
			OHS/OKS			
Linear term	3.100E-06	***	1.970E-07	2.670E-06	***	2.920E-07
Quadratic term	-2.580E-07	***	2.500E-08	-2.260E-07	***	3.580E-08
Cubic term	1.070E-08	***	1.400E-09	9.700E-09	***	1.960E-09
Quartic term	-2.080E-10	***	3.500E-11	-2.020E-10	***	4.870E-11
Quintic term	1.500E-12	***	3.220E-13	1.600E-12	***	4.470E-13
			Age			
Linear term	-8.280E-07		1.220E-06	7.640E-07	***	6.090E-08
Quadratic term	4.310E-08		4.730E-08	-7.760E-09	***	4.460E-10
Cubic term	-8.870E-10		8.730E-10	-	-	-
Quartic term	8.530E-12		7.740E-12	-	-	-
Quintic term	-3.510E-14		2.650E-14	-	-	-
Female sex	7.440E-07	***	9.180E-08	-2.470E-07	*	1.110E-07
Constant term	1.717E-04	***	1.190E-05	1.377E-04	***	2.210E-06
N	286,507			308,638		
Data	PROMs-HES			PROMs-HES		
			Candidate models			
Model class	OLS, gamma-GLM with inverse link, gamma-GLM with log-link, Gaussian-GLM with log-link			OLS, gamma-GLM with inverse link, gamma-GLM with log-link, Gaussian-GLM with log-link		
OHS/OKS trend	Linear, cubic, quartic, quintic, sextic, log-linear			Linear, cubic, quartic, quintic, sextic, log-linear		
Age trend	Linear, quadratic, cubic, quartic, quintic, five-year bands, exclusion			Linear, quadratic, cubic, five-year bands, ten-year bands, exclusion		
Sex	yes/no			yes/no		

Sources: NHS PROMs and HES data, 2009-2015; own calculations.* p<0.05; ** p<0.01; *** p<0.001. Details on how to calculate predictions are provided in Appendix D.

Table C.7: Associations between pre-operative OHS/OKS and the costs of revision arthroplasty

	Hip arthroplasty			Knee arthroplasty		
Model	*gamma-GLM with inverse link function*			*OLS*		
**Variable**	**Coefficient**		**Clustered S.E.**	**Coefficient**		**Clustered S.E.**
			OHS/OKS			
Linear term	-2.370E-07	*	9.790E-08	-1.404E+00		6.816E+00
			Age			
Linear term	9.060E-07	*	3.500E-07	-2.101E+01	***	4.846E+00
Quadratic term	-6.620E-09	*	3.010E-09	-	-	-
Female sex	6.700E-06	***	1.570E-06	7.458E+01		9.551E+01
Constant term	9.570E-05	***	1.000E-05	9.111E+03	***	3.275E+02
N	2,359			3,416		
Data	PROMs-HES			PROMs-HES		
			Candidate models			
Model class	OLS, gamma-GLM with inverse link, gamma-GLM with log-link, Gaussian-GLM with log-link			OLS, gamma-GLM with inverse link, gamma-GLM with log-link, Gaussian-GLM with log-link		
OHS/OKS trend	Linear, cubic, log-linear			Linear, cubic, quartic, log-linear		
Age trend	Linear, quadratic, cubic, exclusion			Linear, quadratic, cubic, exclusion		
Sex	yes/no			yes/no		

Sources: NHS PROMs and HES data, 2009-2015; own calculations.* p<0.05; ** p<0.01; *** p<0.001. Details on how to calculate predictions are provided in Appendix D.

Table C.8: Associations between pre-operative OHS/OKS and costs in the 12 months leading up to TJR

	Hip arthroplasty			Knee arthroplasty		
Model	*gamma-GLM with log-link*			*gamma-GLM with log-link*		
**Variable**	**Coefficient**		**S.E.**	**Coefficient**		**S.E.**
			OHS/OKS			
Log-linear term	-3.485E-01	**	1.177E-01	-	-	-
Linear term	-	-	-	4.307E-02		7.466E-02
Quadratic term	-	-	-	-1.580E-03		1.741E-03
			Age			
Linear term	-8.699E-02		4.441E-02	-2.131E-02		1.430E-02
Quadratic term	5.482E-04		3.489E-04	-	-	-
Female sex	-	-	-	-	-	-
Constant term	1.033E+01	***	1.429E+00	8.027E+00	***	1.302E+00
N	441			278		
Data	COASt			COASt		
			Candidate models			
Model class	OLS, Tobit censored at zero, gamma-GLM with log-link, two-part (Logit + OLS/gamma-GLM with log-link/gamma-GLM with inverse link)			OLS, Tobit censored at zero, gamma-GLM with log-link, two-part (Logit + OLS/gamma-GLM with log-link/gamma-GLM with inverse link)		
OHS/OKS trend	Linear, quadratic, cubic, log-linear			Linear, quadratic, cubic		
Age trend	Linear, quadratic, cubic, 20-year bands, exclusion			Linear, quadratic, cubic, 20-year bands, exclusion		
Sex	yes/no			yes/no		

Sources: COASt data; own calculations.* p<0.05; ** p<0.01; *** p<0.001. Details on how to calculate predictions are provided in Appendix D. For observations with OHS=0, we set ln(OHS)=0.

Table C.9: Associations between pre-operative OHS/OKS and ambulatory costs within 12 months after primary arthroplasty

	Hip arthroplasty			Knee arthroplasty		
Model	*OLS Regression*			*gamma-GLM with log-link*		
**Variable**	**Coefficient**		**Robust S.E.**	**Coefficient**		**Robust S.E.**
			OHS/OKS			
Linear term	2.881E+01	**	8.642E+00	-1.552E-02	***	3.408E-03
Quadratic term	-1.403E+00	**	4.400E-01	-	-	-
Cubic term	1.827E-02	**	6.815E-03	-	-	-
			Age			
Linear term	-2.670E+00	*	1.128E+00	-2.908E-02	***	3.060E-03
Female sex	-	-	-	-	-	-
Constant term	1.371E+02		9.342E+01	8.170E+00	***	2.203E-01
N	548			1,841		
Data	COASt			KAT		
			Candidate models			
Model class	OLS, Tobit censored at zero, gamma-GLM with inverse link, gamma-GLM with log-link, Gaussian-GLM with log-link, two-part (Logit + OLS/gamma-GLM with inverse link/gamma-GLM with log-link/Gaussian-GLM with log-link)			OLS, Tobit censored at zero, gamma-GLM with inverse link, gamma-GLM with log-link, Gaussian-GLM with log-link, two-part (Logit + OLS/gamma-GLM with inverse link/gamma-GLM with log-link/Gaussian-GLM with log-link)		
OHS/OKS trend	Linear, quadratic, cubic, linear spline (20)			Linear, cubic, linear spline (25)		
Age trend	Linear, quadratic, cubic, exclusion			Linear, quadratic, cubic, log-linear, exclusion		
Sex	yes/no			yes/no		

Sources: COASt and KAT data; own calculations.* p<0.05; ** p<0.01; *** p<0.001. Details on how to calculate predictions are provided in Appendix D.

Table C.10: Associations between pre-operative OHS/OKS and the costs of hospital readmissions within 12 months after primary arthroplasty

	Hip arthroplasty			Knee arthroplasty		
Model	*Gaussian-GLM with log-link function*			*Gaussian-GLM with log-link function*		
**Variable**	**Coefficient**		**Clustered S.E.**	**Coefficient**		**Clustered S.E.**
			OHS/OKS			
Linear term	-1.727E-01	***	3.719E-02	-1.177E-01	***	1.342E-02
Quadratic term	1.325E-02	**	5.060E-03	3.638E-03	***	7.422E-04
Cubic term	-6.122E-04	*	2.955E-04	-4.350E-05	***	1.230E-05
Quartic term	1.370E-05		7.640E-06	-	-	-
Quintic term	-1.140E-07		7.160E-08	-	-	-
			Age			
Linear term	5.318E-03	**	1.671E-03	-8.114E-02	***	9.237E-03
Linear spline (age ≥70)	3.448E-02	***	3.360E-03	-	-	-
Quadratic term	-	-	-	7.103E-04	***	6.670E-05
Female sex	-1.785E-01	***	2.144E-02	-2.500E-01	***	2.070E-02
Constant term	6.788E+00	***	1.476E-01	9.687E+00	***	3.008E-01
N	236,514			255,194		
Data	PROMs-HES			PROMs-HES		
			Candidate models			
Model class	OLS, Tobit censored at zero, two-part (Logit + OLS/gamma-GLM with inverse link/gamma-GLM with log-link/Gaussian-GLM with log-link), gamma-GLM with inverse link, gamma-GLM with log-link, Gaussian-GLM with log-link			OLS, Tobit censored at zero, two-part (Logit + OLS/gamma-GLM with inverse link/gamma-GLM with log-link/Gaussian-GLM with log-link), gamma-GLM with inverse link, gamma-GLM with log-link, Gaussian-GLM with log-link		
OHS/OKS trend	Linear spline (10), quadratic, cubic, quartic, quintic			Linear spline (10), quadratic, cubic, quartic, quintic		
Age trend	Linear spline (70), linear, quadratic, cubic, exclusion			Linear spline (70), linear, quadratic, cubic, exclusion		
Sex	yes/no			yes/no		

Sources: NHS PROMs and HES data, 2009-2015; own calculations.* p<0.05; ** p<0.01; *** p<0.001. Details on how to calculate predictions are provided in Appendix D. The linear spline term is set to zero for patients aged below 70. For patients above 70, the term equals (age in years -70).

Table C.11: Associations between pre-operative OHS/OKS and annual costs of hospital readmissions between 1 and 6 years after primary arthroplasty

	Hip arthroplasty			Knee arthroplasty		
Model	*gamma-GLM with inverse link function*			*gamma-GLM with inverse link function*		
**Variable**	**Coefficient**		**Clustered S.E.**	**Coefficient**		**Clustered S.E.**
OHS/OKS						
Linear term	1.998E-04	***	1.660E-05	1.655E-04	***	8.150E-06
Current age						
Linear term	4.230E-05	***	9.000E-06	-	-	-
Age at primary arthroplasty						
Linear term	-	-	-	4.600E-05	***	5.300E-06
Time trend						
Linear term	-	-	-	1.227E-03	***	6.870E-05
Year 2 since primary surgery	-2.446E-03	***	2.263E-04	-	-	-
Female sex	-	-	-	4.290E-04	***	1.138E-04
Constant term	3.432E-03	***	7.112E-04	-3.965E-03	***	3.662E-04
N	476,514			514,047		
Data	PROMs-HES			PROMs-HES		
Candidate models						
Model class	OLS, Tobit censored at zero, two-part (Logit + OLS/gamma-GLM with inverse link/gamma-GLM with log-link/Gaussian-GLM with log-link), gamma-GLM with inverse link, gamma-GLM with log-link, Gaussian-GLM with log-link			OLS, Tobit censored at zero, two-part (Logit + OLS/gamma-GLM with inverse link/gamma-GLM with log-link/Gaussian-GLM with log-link), gamma-GLM with inverse link, gamma-GLM with log-link, Gaussian-GLM with log-link		
Year since primary surgery (time trend)	Linear, quadratic, binary indicator for year 2, binary indicators for all years, exclusion			Linear, quadratic, binary indicator for year 2, binary indicators for all years, exclusion		
OHS/OKS trend	Linear, quadratic, cubic, quartic, quintic, log-linear			Linear, quadratic, cubic, quartic, quintic, log-linear		
Age trend	Age at operation vs. current age; for current age: Linear, quadratic, cubic, five-year age bands, exclusion			Age at operation vs. current age; for age at operation: Linear, quadratic, cubic, quartic, quintic, five-year age bands, exclusion		
Sex	yes/no			yes/no		

Sources: NHS PROMs and HES data, 2009-2015; own calculations.* p<0.05; ** p<0.01; *** p<0.001. Details on how to calculate predictions are provided in Appendix D. "Year 2 since primary surgery" is a binary indicator. "Year 1" refers to the first 12 months after primary surgery.

Table C.12: Associations between pre-operative OHS/OKS and the costs of hospital readmissions during the year of revision surgery

	Hip arthroplasty			Knee arthroplasty		
Model	*Two-part model: Logit + gamma-GLM with inverse link*			*Two-part model: Logit + gamma-GLM with log-link*		
**Variable**	**Coefficient**		**Clustered S.E.**	**Coefficient**		**Clustered S.E.**
	**Part 1: Logit model**					
	OHS/OKS					
Linear term	8.407E-03		1.093E-02	3.790E-03		8.559E-03
	Age at primary arthroplasty					
Linear term	9.589E-02	*	4.418E-02	5.140E-04		6.716E-03
Quadratic term	-8.234E-04	*	3.622E-04	-	-	-
	Time trend					
Linear term	-	-	-	-2.488E+00		1.438E+00
Quadratic term	-	-	-	6.157E-01		3.933E-01
Cubic term	-	-	-	-4.586E-02		3.340E-02
Year 2 since primary surgery	3.153E-01		1.675E-01	-	-	-
Female sex	1.724E-01		1.624E-01	2.386E-01		1.235E-01
Constant term	-8.202E-01		1.353E+00	4.622E+00	**	1.699E+00
	**Part 2: gamma-GLM**					
	OHS/OKS					
Linear term	-1.340E-06		9.040E-07	-1.291E-02		7.915E-03
	Age at primary arthroplasty					
Linear term	-9.550E-07		5.400E-06	1.018E-02		5.999E-03
Quadratic term	-4.800E-09		4.080E-08	-	-	-
	Time trend					
Linear term	-	-	-	-3.169E-01		1.725E+00
Quadratic term	-	-	-	1.474E-01		4.819E-01
Cubic term	-	-	-	-1.556E-02		4.192E-02
Year 2 since primary surgery	4.800E-05	**	1.760E-05	-	-	-
Female sex	5.210E-05	*	2.040E-05	-9.075E-02		1.228E-01
Constant term	2.078E-04		1.764E-04	8.352E+00	***	1.982E+00
N	1,669			2,258		
Data	PROMs-HES			PROMs-HES		

...continued on next page

	Candidate models	
Model class	OLS, Tobit censored at zero, two-part (Logit + OLS/gamma-GLM with inverse link/gamma-GLM with log-link/Gaussian-GLM with log-link), gamma-GLM with inverse link, gamma-GLM with log-link, Gaussian-GLM with log-link	OLS, Tobit censored at zero, two-part (Logit + OLS/gamma-GLM with inverse link/gamma-GLM with log-link/Gaussian-GLM with log-link), gamma-GLM with inverse link, gamma-GLM with log-link, Gaussian-GLM with log-link
Year since primary surgery (time trend)	Binary indicator for year 2, exclusion	Linear, quadratic, cubic, exclusion
OHS/OKS trend	Linear, quadratic, cubic	Linear, quadratic, cubic
Age trend	Age at operation vs. current age; for age at operation: Linear, quadratic, cubic, five-year age bands, exclusion	Age at operation vs. current age; for age at operation: Linear, quadratic, cubic, five-year age bands, exclusion
Sex	yes/no	yes/no

Sources: NHS PROMs and HES data, 2009-2015; own calculations.* p<0.05; ** p<0.01; *** p<0.001. Details on how to calculate predictions are provided in Appendix D. "Year 2 since primary surgery" is a binary indicator. "Year 1" refers to the first 12 months after primary surgery.

Table C.13: Associations between pre-operative OHS/OKS and annual costs of hospital readmissions for patient with a previous revision

	Hip arthroplasty			Knee arthroplasty		
Model	*Tobit model censored at zero*			*Tobit model censored at zero*		
**Variable**	**Coefficient**		**Clustered S.E.**	**Coefficient**		**Clustered S.E.**
			OHS/OKS			
Linear term	-2.424E+02	*	1.074E+02	-1.972E+02	*	9.961E+01
			Current age			
Linear term	-	-	-	-1.172E+04	*	4.527E+03
Quadratic term	-	-	-	1.772E+02	*	7.058E+01
Cubic term	-	-	-	-8.911E-01	*	3.609E-01
			Time trend			
Linear term	-	-	-	-1.935E+03	***	4.973E+02
Constant term	-2.454E+04	***	3.657E+03	2.455E+05	**	9.386E+04
σ (Tobit term)	1.472E+04	-	1.859E+03	1.390E+04		2.139E+03
N	2,406			3,153		
Data	PROMs-HES			PROMs-HES		
			Candidate models			
Model class	OLS, Tobit censored at zero, two-part (Logit + OLS/gamma-GLM with inverse link/gamma-GLM with log-link/Gaussian-GLM with log-link), gamma-GLM with inverse link, gamma-GLM with log-link, Gaussian-GLM with log-link			OLS, Tobit censored at zero, two-part (Logit + OLS/gamma-GLM with inverse link/gamma-GLM with log-link/Gaussian-GLM with log-link), gamma-GLM with inverse link, gamma-GLM with log-link, Gaussian-GLM with log-link		
Year since primary surgery (time trend)	Linear, quadratic, exclusion			Linear, quadratic, cubic, exclusion		
OHS/OKS trend	Linear, quadratic, cubic			Linear, quadratic, cubic, quartic, quintic, log-linear		
Age trend	Age at operation vs. current age; for age at operation: linear, quadratic, cubic, five-year age bands, exclusion			Age at operation vs. current age; forcurrent age: linear, quadratic, cubic, exclusion		
Sex	yes/no			yes/no		

Sources: NHS PROMs and HES data, 2009-2015; own calculations.* p<0.05; ** p<0.01; *** p<0.001. Details on how to calculate predictions are provided in Appendix D.

Table C.14: Associations between pre-operative OKS and annual ambulatory costs between 1 and 12 years after TKR

	Knee arthroplasty		
Model	*Gaussian-GLM with log-link function*		
**Variable**	**Coefficient**		**Clustered S.E.**
	OKS		
Linear term	1.125E-03		9.113E-04
Quadratic term	-5.597E-01	*	2.420E-01
	Age at primary operation		
Linear term	3.672E-01		2.467E-01
Quadratic term	-7.167E-03		4.043E-03
Cubic term	4.130E-05		2.150E-05
	Year since primary surgery (time trend)		
Linear term	-5.597E-01	*	2.420E-01
Quadratic term	5.295E-02		4.104E-02
Cubic term	-1.810E-03		2.111E-03
Female sex	-3.197E-01	*	1.363E-01
Constant term	1.212E+00		4.801E+00
N	13,271		
Data	KAT		
	Candidate models		
Model class	OLS, Tobit censored at zero, two-part (Logit + OLS/gamma-GLM with log-link), gamma-GLM with inverse link, gamma-GLM with log-link, Gaussian-GLM with log-link		
Time trend	Linear, quadratic, cubic trend, binary indicator for year 2, binary indicator for year 2 and a linear trend, exclusion		
OKS trend	Linear, quadratic, cubic		
Age trend	Age at operation vs. current age; for age at operation: Linear, quadratic, cubic, exclusion		
Sex	yes/no		

Sources: KAT data; own calculations.* p<0.05; ** p<0.01; *** p<0.001. Details on how to calculate predictions are provided in Appendix D. The model was estimated on data in long-format (i.e., one observation per patient and year). The inclusion of the time trend was tested after selecting the model class and before choosing a trend for OKS. We did not estimate a model for hips due to a lack of data.

Table C.15: Associations between pre-operative OKS and ambulatory costs in the year of revision surgery

	Knee arthroplasty		
Model	*gamma-GLM with log-link function*		
**Variable**	**Coefficient**		**Clustered S.E.**
	OKS		
Linear term	-3.271E-02		1.805E-02
	Age at primary operation		
Linear term	2.062E-01	*	8.868E-02
Quadratic term	-1.777E-03	**	6.774E-04
Constant term	1.145E+00		2.861E+00
N	88		
Data	KAT		
	Candidate models		
Model class	OLS, Tobit censored at zero, two-part (Logit + OLS/gamma-GLM with log-link/gamma-GLM with inverse link/Gaussian-GLM with log-link), gamma-GLM with inverse link, gamma-GLM with log-link, Gaussian-GLM with log-link		
OKS trend	Linear, quadratic, cubic		
Age trend	Linear, quadratic, cubic, exclusion		
Sex	yes/no		

Sources: KAT data; own calculations.* p<0.05; ** p<0.01; *** p<0.001. Details on how to calculate predictions are provided in Appendix D. The model was estimated on data in long-format (i.e., one observation per patient and year). We did not estimate a model for hips due to a lack of data.

Table C.16: Associations between pre-operative OKS and annual ambulatory costs for patients with a previous revision

	Knee arthroplasty		
Model	*gamma-GLM with inverse link function*		
**Variable**	**Coefficient**		**Clustered S.E.**
	OKS		
Linear term	1.637E-03		9.185E-04
Quadratic term	-1.439E-04		7.930E-05
Cubic term	3.860E-06	*	1.890E-06
Female sex	1.726E-03		2.068E-03
Constant term	3.737E-04		1.859E-03
N	329		
Data	KAT		
	Candidate models		
Model class	OLS, Tobit censored at zero, two-part (Logit + OLS/gamma-GLM with log-link/gamma-GLM with inverse link/Gaussian-GLM with log-link), gamma-GLM with inverse link, gamma-GLM with log-link, Gaussian-GLM with log-link		
OKS trend	Linear, quadratic, cubic		
Age trend	Linear, quadratic, cubic, exclusion		
Sex	yes/no		

Sources: KAT data; own calculations.* p<0.05; ** p<0.01; *** p<0.001. Details on how to calculate predictions are provided in Appendix D. Notes: The model was estimated on data in long-format (i.e., one observation per patient and year). We did not estimate a model for hips due to a lack of data.

D. Note on calculating predictions

1) OLS models

The predicted value of the outcome variable, $\hat{y}$ is simply the linear predictor Xb, which is obtained by multiplying each variable with its estimated coefficient and then summing over all variables.

Example:

For a patient aged 60 and with an OHS of 18, the ambulatory costs in the first 12 months after primary THR (Table C.9) can be predicted as:

$$\begin{aligned}\cos t &= 28.81\times OHS - 1.40\times OHS^2 + 0.02\times OHS^3 - 2.67\times age + 137.10 \\ &= 28.81\times 18 - 1.40\times 324 + 0.02\times 5832 - 2.67\times 60 + 137.10 \\ &= 147.46\end{aligned}$$

2) Gamma-GLM with an inverse link function

Predicted values for the outcome variable can be obtained as $\hat{y} = \frac{1}{Xb}$.

Example:

For a female patient with OKS of 15, the annual ambulatory costs after revision TKR surgery (Table C.16) can be calculated as follows:

$$\begin{aligned}\cos t &= \frac{1}{0.000374 + 0.001726\times female + 0.001637\times OKS - 0.000144\times OKS^2 + 0.000004\times OKS^3} \\ &= \frac{1}{0.000374 + 0.001726\times 1 + 0.001637\times 15 - 0.000144\times 225 + 0.000004\times 3375^3} \\ &= \frac{1}{0.007305} \\ &= 136.90\end{aligned}$$

3) Gamma-GLM with a log-link function

Predicted values for the outcome variable can be obtained as $\hat{y} = e^{Xb}$.

Example: For a patient aged 60 at the time of her primary TKR operation and with an OKS of 10, the ambulatory cost in the year of revision surgery (Table C.15) can be calculated as follows:

$$\begin{aligned}\cos t &= e^{1.145-0.033\times OKS+0.206\times age-0.002\times age^2}\\ &= e^{1.145-0.033\times 10+0.206\times 60-0.002\times 3600}\\ &= e^{6.7927}\\ &= 2.7182^{6.7927}\\ &= 891.32\end{aligned}$$

4) Gaussian-GLM with a log-link functio

Predicted values for the outcome variable can be obtained as $\hat{y} = e^{Xb}$.

Example: For a female patient aged 70 and with an OKS of 70, the cost of hospital readmission in the first 12 months after primary TKR (Table C.10) can be calculated as follows:

$$\begin{aligned}\cos t &= e^{9.68700-0.25\times female-0.11770\times OKS+0.00364\times OKS^2-0.00004\times OKS^3-0.08114\times age+0.00071\times age^2}\\ &= e^{9.68700-0.25\times 1-0.11770\times 20+0.00364\times 400-0.00004\times 8000-0.08114\times 70+0.00071\times 4900}\\ &= e^{5.99087}\\ &= 2.7182^{5.99087}\\ &= 399.76\end{aligned}$$

5) Tobit models censored at zero

The predicted value is given by $\hat{y} = -\Phi\left(\frac{Xb}{\sigma}\right)Xb + \sigma\phi\left(\frac{Xb}{\sigma}\right)$

where $\Phi(\cdot)$ denotes the cumulative distribution function of the standard normal distribution, and $\phi(\cdot)$ denotes the probability density function of the standard normal distribution.

Example: For a patient with an OHS of 25, the annual cost of hospital readmissions after revision surgery (Table C.13) can be calculated as follows:

$$
\begin{aligned}
\cos t &= -\Phi\left(\frac{-24540-242.40\times OHS}{14720}\right)(-24540-242.40\times OHS)+14720\phi\left(\frac{-24540-242.40\times OHS}{14720}\right)\\
&= -\Phi\left(\frac{-24540-242.40\times 20}{14720}\right)(-24540-242.40\times 20)+14720\phi\left(\frac{-24540-242.40\times 20}{14720}\right)\\
&= -\Phi\left(-1.99647\right)(-29388)+14720\phi\left(-1.99647\right)\\
&= 0.022942\times(-29388)+14720\times 0.054373\\
&= 126.15
\end{aligned}
$$

6) Tobit models censored at -0.594 and 1

The predicted value is given by

$$
\hat{y} = -0.594*\Phi\left(\frac{-0.594-Xb}{\sigma}\right)+1*\Phi\left(-\frac{1-Xb}{\sigma}\right)
$$

$$
+\left(\Phi\left(\frac{1-Xb}{\sigma}\right)-\Phi\left(\frac{-0.594-Xb}{\sigma}\right)\right)*\left(Xb-\sigma*\frac{\phi\left(\frac{1-Xb}{\sigma}\right)-\phi\left(\frac{-0.594-Xb}{\sigma}\right)}{\Phi\left(\frac{1-Xb}{\sigma}\right)-\Phi\left(\frac{-0.594-Xb}{\sigma}\right)}\right)
$$

where $\Phi(\cdot)$ denotes the cumulative distribution function of the standard normal distribution, and $\phi(\cdot)$ denotes the probability density function of the standard normal distribution.

Example: For a patient aged 75 in the year of THR revision surgery and with a pre-operative (pre-primary) OHS of 8, the EQ-5D utility before revision surgery (Table C.4) can be calculated with the following steps.

First, the linear predictor (*Xb*) is calculated as:

$$
\begin{aligned}
Xb &= -0.1444+0.0021\times currentage+0.0267\times OHS-0.0003\times OHS^2\\
&= -0.1444+0.0021\times 75+0.0267\times 8-0.0003\times 64\\
&= 0.21
\end{aligned}
$$

Then, using this value and the formula above the predicted EQ-5D utility can be calculated as follows:

$$QoL = -0.594 \times \Phi\left(\frac{-0.594-0.21}{0.3178}\right) + 1 \times \Phi\left(-\frac{1-0.21}{0.3178}\right)$$

$$+\left(\Phi\left(\frac{1-0.21}{0.3178}\right) - \Phi\left(\frac{-0.594-0.21}{0.3178}\right)\right) \times \left(0.21 - 0.3178 \times \frac{\phi\left(\frac{1-0.21}{0.3178}\right) - \phi\left(\frac{-0.594-0.21}{0.3178}\right)}{\Phi\left(\frac{1-0.21}{0.3178}\right) - \Phi\left(\frac{-0.594-0.21}{0.3178}\right)}\right)$$

$$= -0.594 \times 0.005705 + 1 \times 0.006462 + (0.993538 - 0.005705) \times \left(0.21 - 0.3178 \times \frac{0.018158 - 0.016259}{0.993538 - 0.005705}\right)$$

$$= -0.00339 + 0.006462 + 0.987833 \times (0.21 - 0.000611)$$

$$= 0.209913$$

7) Two-part models

The predicted value from a two-part model is given by

$\hat{y} = \left(1 - P\left[y = 0 \mid X\right]\right) * E\left[y \mid y > 0, X\right]$.

$E\left[y \mid y > 0, X\right]$ is given by the predicted value in the second part of the model.

$P\left[y = 0 \mid X\right]$ is given as $P\left[y = 0 \mid X\right] = \frac{e^{Xb}}{1 + e^{Xb}}$ if the first part of the model is a logit model.

Example: For a female patient aged 65 in the year of her primary THR operation and with an OHS of 15, the costs of hospital readmissions in the year of revision surgery in year 3 since her primary operation (Table C.12) can be calculated with the following steps:

First, we calculate the linear predictor, *Xb*, of the first part of the model:

$$\begin{aligned} Xb_1 &= -0.820 + 0.172 \times female + 0.315 \times year2 + 0.008 \times OHS + 0.096 \times age - 0.001 \times age^2 \\ &= -0.820 + 0.172 \times 1 + 0.315 \times 0 + 0.008 \times 15 + 0.096 \times 65 - 0.001 \times 4225 \\ &= 2.23229 \end{aligned}$$

Then, the probability of zero cost can be calculated as follows:

$$\begin{aligned} P\left[\cos t = 0 \mid Xb\right] &= \frac{e^{Xb}}{1 + e^{Xb}} \\ &= \frac{e^{2.23229}}{1 + e^{2.23229}} \\ &= \frac{2.718281^{2.23229}}{1 + 2.718281^{2.23229}} \\ &= \frac{9.321187}{1 + 9.321187} \\ &= 0.903112 \end{aligned}$$

Next, the linear predictor of the second part of the model is calculated as:

$$Xb_2 = 0.0002078 + 0.0000521 \times female + 0.000048 \times year2 - 0.00000134 \times OHS$$
$$-0.000000955 \times age - -0.000000005 \times age^2$$
$$= 0.0002078 + 0.0000521 \times 1 + 0.000048 \times 0 - 0.00000134 \times 15$$
$$-0.000000955 \times 65 - -0.000000005 \times 4225$$
$$= 0.000157$$

Then, the predicted costs of the second part of the model can be calculated as:

$$E[\cos t \mid \cos t > 0, Xb] = \frac{1}{Xb}$$
$$= \frac{1}{0.000157}$$
$$= 6351.424$$

The formula used here is the prediction formula for a gamma-GLM with an inverse link function (see section 2), since this is the model used for the second part in Table C.12. The first part of the model predicts that for the patient described in this example the probability of incurring no cost for hospital readmissions in the year of revision surgery is 90.3%. The second part predicts that if the patient has a hospital admission, than the predicted annual costs for readmissions are £6351.4.

These two results can be combined to derive the predicted cost of hospital readmissions as follows:

$$\cos t = \left(1 - P[y = 0 \mid X]\right) * E[y \mid y > 0, X]$$
$$= (1 - 0.903112) * 6351.424$$
$$= 615.38$$

This values takes into account that there is a high probability that the cost are zero.
