# Supplementary material for: Associations between preoperative Oxford hip and knee scores and costs and quality of life of patients undergoing primary total joint replacement in the NHS England: an observational study
Source: BMJ Open. 2018 Apr 10;8(4):e019477. doi: 10.1136/bmjopen-2017-019477 (PMC5898298; doi:10.1136/bmjopen-2017-019477)
Supplement: Author's manuscript [file bmjopen-2017-019477.draft_revisions.pdf]

BMJ Open is committed to open peer review. As part of this commitment we make the peer review history of every article we publish publicly available.

When an article is published we post the peer reviewers' comments and the authors' responses online. We also post the versions of the paper that were used during peer review. These are the versions that the peer review comments apply to.

The versions of the paper that follow are the versions that were submitted during the peer review process. They are not the versions of record or the final published versions. They should not be cited or distributed as the published version of this manuscript.

BMJ Open is an open access journal and the full, final, typeset and author-corrected version of record of the manuscript is available on our site with no access controls, subscription charges or pay-per-view fees (http://bmjopen.bmj.com).

If you have any questions on BMJ Open's open peer review process please email info.bmjopen@bmj.com

BMJ Open

Association between pre-operative Oxford hip and knee scores and costs and quality-of-life of patients undergoing primary total joint replacement

Journal:	*BMJ Open*
Manuscript ID	bmjopen-2017-019477
Article Type:	Research
Date Submitted by the Author:	04-Sep-2017
Complete List of Authors:	Eibich, Peter; University of Oxford, Health Economics Research Centre, Nuffield Department of Population Health<br>Dakin, Helen; University of Oxford, Health Economics Research Centre, Nuffield Department of Population Health<br>Price, Andrew; University of Oxford, Nuffield Department of Orthopaedics, Rheumatology and Musculoskeletal Sciences<br>Beard, David; University of Oxford, Nuffield Dept of Orthopaedics, Rheumatology and Musculoskeletal Sciences<br>Arden, Nigel; Nuffield Department of Orthopaedics, ;<br>Gray, Alastair; University of Oxford, Nuffield Department of Population Health
**Primary Subject Heading**:	Rheumatology
Secondary Subject Heading:	Health policy
Keywords:	hip replacement, knee replacement, costs, quality-of-life, Oxford Hip Score, Oxford Knee Score

SCHOLARONE™
Manuscripts

Association between pre-operative Oxford hip and knee scores and costs and quality-of-life of patients undergoing primary total joint replacement

Peter Eibich[1], Helen Dakin[1], Andrew Price[2], David Beard[2], Nigel Arden[2,3] and Alastair M. Gray[1]

[1] Health Economics Research Centre, Nuffield Department of Population Health, University of Oxford, Oxford, UK

[2] Nuffield Department of Orthopaedics, Rheumatology and Musculoskeletal Sciences, University of Oxford, Oxford, UK

[3] Faculty of Medicine, University of Southampton, Southampton, UK

For peer review only

Running title: Associations between OHS/OKS and costs and quality-of-life

4723 words

2 tables

3 figures

Corresponding author:

Peter Eibich, Nuffield Department of Population Health, University of Oxford, Old Road Campus, Headington, OX3 7LF Oxford, UK. Tel.: +44 1865 289272. E-mail: peter.eibich@dph.ox.ac.uk

For peer review only - http://bmjopen.bmj.com/site/about/guidelines.xhtml

Abstract

Objectives: To assess how costs and quality-of-life before and after total hip and knee replacement (THR/TKR) vary with age, gender and pre-operative Oxford hip and knee scores (OHS/OKS).

Design: Regression analyses using prospectively collected data from clinical trials, cohort studies and administrative data bases.

Setting: UK secondary care.

Participants: Men and women undergoing primary THR or TKR. The Hospital Episode Statistics data linked to patient reported outcome measures included 602,176 patients undergoing hip or knee replacement who were followed up for up to six years. The Knee Arthroplasty Trial included 2,217 TKR patients who were followed up for 10 years. The Clinical Outcomes in Arthroplasty Study cohort included 806 THR and 484 TKR patients who were observed for one year.

Outcome measures: EQ-5D-3L quality-of-life before and after surgery, costs of primary arthroplasty, costs of revision arthroplasty and the costs of hospital readmissions and ambulatory costs in the year before and up to 12 years after joint replacement.

Results: The relationship between pre-operative OHS/OKS and post-operative EQ-5D utility was non-linear, rising more slowly with OHS/OKS for scores above 10. The difference between post- and pre-operative EQ-5D utility was therefore highest for patients with pre-operative OHS/OKS lower than 10. However, post-operative EQ-5D utility was higher than pre-operative utility (indicating that EQ-5D utility has improved following arthroplasty) for all patients with OHS ≤46 and those with OKS ≤44. In contrast, the cost of primary arthroplasty and hospital readmissions were higher for patients with low preoperative OHS/OKS than those with high OHS/OKS. Age and gender were less marked than associations with OHS or OKS.

Conclusions: Our findings suggest that the costs and quality-of-life changes associated with total joint replacement vary with symptoms and (to a lesser extent) with age and sex.

287 words

Keywords: Total joint replacement, hip replacement, knee replacement, costs, quality-of-life, Oxford Hip Score, Oxford Knee Score, regression model.

For peer review only - http://bmjopen.bmj.com/site/about/guidelines.xhtml

Strengths and limitations of this study

- This study used data from different sources, including clinical trials, cohort studies and administrative databases.
- The sample sizes are considerably larger than those used in previous studies.
- The optimal regression models were identified in a systematic model selection process designed to maximise predictive accuracy.
- Our largest dataset contained no information on potentially important variables such as body mass index.

For peer review only

For peer review only - http://bmjopen.bmj.com/site/about/guidelines.xhtml

INTRODUCTION

Total joint replacement (TJR) has been shown to greatly improve the quality-of-life of patients with osteoarthritis.[1–5] Moreover, previous studies have demonstrated that both total knee replacement (TKR) and total hip replacement (THR) are highly cost-effective.[3,4] Nevertheless, in the UK access to TJR surgery is frequently rationed based on patient characteristics (such as age and functional status),[6] since it is a non-urgent procedure with a high impact on healthcare budgets. In particular, the Oxford hip and knee scores (OHS/OKS) are commonly used to define threshold values (varying from 19 to 30) above which patients are not eligible for TJR.[7–10] This is based on the argument that patients with lower pre-operative functional status benefit more from TJR, but the evidence to support this is limited, and very few studies have examined how outcomes or costs of TJR differ by pre-operative patient characteristics.[1–5,11–16]

Most of these studies focused on demographic characteristics such as age, with only five studies exploring the impact of pre-operative function.[3,4,13,14,16] Cushnaghan et al.[13] examined associations between baseline physical functioning (measured by SF-36) and the long-term outcome of TKR (measured by the change in SF-36). Judge et al.[16] investigated associations between pre-operative OHS and post-operative OHS and concluded that all patients benefitted from substantially from THR. Only Dakin et al.[4] and Fordham et al.[3] investigated the impact of OKS and OHS on costs and outcomes of TKR and TJR, respectively, and both studies concluded that while health benefits and costs do vary with pre-operative OHS/OKS score, TJR improves quality-of-life for almost all patients.

One recent US study by Ferket et al.[14] concluded that TKR results in only minimal health gains for patients with good physical function (measured by the SF-12 physical score), and that, on economic grounds, access to TKR should be restricted to patients with severe symptoms; however this study was based on only 382 TKR patients. Other studies in this area have also relied on small datasets (e.g., 2,138 TKR[4] or 938 THR patients[3] followed for five years), which are likely to contain very few patients with low or high scores. The results from these studies are therefore subject to considerable uncertainty.

This study aimed to estimate the relationship between pre-operative patient characteristics (age, sex and OHS/OKS) and the costs and quality-of-life of patients undergoing TJR in England. We used administrative data from the NHS patient reported outcome measures (PROMs) programme linked to Hospital Episode Statistics (HES) data, alongside smaller datasets from the Knee Arthroplasty Trial (KAT) and the Clinical Outcomes in Arthroplasty Study (COASt) cohort study to obtain reliable estimates on a wide range of outcomes and patients, including for patients with very high OHS/OKS.

DATA

Datasets

The analyses presented in this study are based on patient-level data from three sources –the KAT trial,[17] the COASt cohort study,[18] and data from the NHS PROMs programme linked to HES data.[19] These datasets were chosen from a wider range of available

For peer review only - http://bmjopen.bmj.com/site/about/guidelines.xhtml

candidate datasets[3,20–22] based on the availability of relevant outcome and baseline data as well as sample size considerations.

The NHS PROMs programme routinely collects data on all patients undergoing hip and knee replacement on the NHS in England.[23] Data are collected via questionnaires at baseline (before surgery) and six months post-surgery. The dataset used in this study covered the period from April 2009 to October 2015. We linked these data to Admitted Patient Care data from HES,[19] which contains routine data on all patients treated in hospitals in the NHS England, with a particular focus on diagnoses and procedures. This yielded a sample of up to 602,176 patients who were followed up for up to six years. The data include patients undergoing primary joint replacement surgery other than TJR (e.g., hip resurfacing), since we were not able to reliably identify the type of operation from the available data.

KAT is a randomised controlled trial comparing different types of knee prosthesis.[17] We used data on EQ-5D utility and ambulatory costs following 2217 UK patients, who were followed up annually for up to 12 years after TKR.

COASt[18] is a prospective, dual-centre longitudinal cohort study recruiting patients across two hospitals: University Hospital Southampton NHS Foundation Trust and Nuffield Orthopaedic Centre (part of Oxford University Hospitals NHS Trust). COASt was established in 2010 and recruited patients placed on the waiting list for knee or hip replacement surgery. The datasets extracted for this study contained observations for 810 hip surgery patients and 858 patients undergoing knee surgery. After excluding patients who underwent procedures other than THR/TKR (e.g., partial knee replacement or hip resurfacing), the datasets contained 806 THR patients and 484 TKR patients. Data are collected prior to surgery as well as at six weeks and then annually for five years thereafter, although only the first year of data were used in this study.

Table 1 below provides an overview over all outcomes for which regression models were developed as well as the dataset used for the analysis, the final sample size and where the results are shown. For brevity, we discuss the findings for selected outcomes in this paper; the models for all other outcomes are provided in the appendix.

For peer review only

Table 1: Data sources and sample sizes

	THR			TKR				
**Outcome**	**Data**	**Final sample size**	**Missing covariates**	**Data**	**Final sample size**	**Missing covariates**	**Table**	**Figure**
Post-operative EQ-5D utility	PROMs-HES	208,344	0.96%	PROMs-HES	223,836	0.96%	2	1.A
Pre-operative EQ-5D utility	PROMs-HES	271,045	0.83%	PROMs-HES	290,983	0.98%	C.1	1.A
Costs of primary arthroplasty	PROMs-HES	286,507	1.12%	PROMs-HES	308,638	1.21%	C.2	1.B
Costs of revision arthroplasty	PROMs-HES	2,359	1.17%	PROMs-HES	3,416	1.13%	C.3	1.C
Costs in the 12 months before arthroplasty	COASt	441	16.00%	COASt	278	10.03%	C.4	2.A
Ambulatory costs <12 months after primary arthroplasty	COASt	548	16.72%	KAT	1,841	3.21%	C.5	2.B
Readmissions <12 months after primary arthroplasty	PROMs-HES	236,514	1.48%	PROMs-HES	255,194	1.22%	C.6	2.C
Readmissions >12 months after primary arthroplasty*	PROMs-HES	476,514	0.98%	PROMs-HES	514,047	1.18%	C.7	3.A
Readmissions in the year of revision surgery	PROMs-HES	1,669	1.07%	PROMs-HES	2,258	1.05%	C.8	3.B
Readmissions > 12 months after revision surgery*	PROMs-HES	2,406	0.87%	PROMs-HES	3,153	1.10%	C.9	3.C
EQ-5D utility > 12 months after primary arthroplasty*	-	-	-	KAT	15,414	0.00%	C.10	-
EQ-5D utility before revision surgery	PROMs-HES	1,391	1.21%	PROMs-HES	2,227	1.07%	C.11	-
EQ-5D utility after revision surgery	PROMs-HES	884	0.90%	PROMs-HES	1,398	0.99%	C.12	-
Ambulatory costs >12 months after primary arthroplasty*	-	-	-	KAT	13,271	4.13%	C.13	-
Ambulatory costs in the year of revision surgery	-	-	-	KAT	88	4.35%	C.14	-
Ambulatory costs for patients after revision surgery*	-	-	-	KAT	329	3.24%	C.15	-

Notes: Tables C.1-C.15 are shown in the online supplementary file. "Missing covariates" shows the fraction of observations that were excluded from the final sample due to missing data on

For peer review only - http://bmjopen.bmj.com/site/about/guidelines.xhtml

For peer review only

one of the covariates (incl. OHS/OKS) included into the model. For models estimated on longitudinal data, the "final sample size" shows the total number of observations, including repeated observations on the same patient.
* Model estimated on longitudinal data.

For peer review only

For peer review only - http://bmjopen.bmj.com/site/about/guidelines.xhtml

Outcomes

Quality-of-life

Pre- and post-operative quality-of-life was measured using EQ-5D-3L, valued using the UK time trade-off tariff.[24] EQ-5D-3L health utility takes values between -0.594 and 1, with higher values representing higher quality-of-life. We chose to focus on EQ-5D, since it is the preferred measure of quality-of-life for economic evaluations submitted to the National Institute for Health and Care Excellence (NICE).

Linked NHS PROMs/HES data were used to estimate quality-of-life before surgery and six months after THR or TKR. Data from KAT were used to estimate annual changes in quality-of-life between six months and 12 years after TKR.

Costs

Primary and revision arthroplasty

We estimated the impact of OHS/OKS on the costs of primary and revision TJR using linked NHS PROMs/HES data. We identified HES observations related to primary or revision arthroplasty by linking NHS PROMs data to the HES database, and then excluded all observations marked as revisions in the corresponding NHS PROMs questionnaire. If a patient was observed to have the same procedure (THR or TKR) carried out at the same joint (right- or left-side) more than once, we assumed that the earliest observed procedure was a primary surgery episode, while all subsequent procedures were revision episodes. Similarly, we identified revisions as those episodes that were either marked as a revision in the NHS PROMs data, or where we observed an earlier procedure on the same joint in the HES data. This implies that for patients undergoing primary joint replacement other than TJR first and TJR on the same joint later, the first procedure would be coded as primary surgery and the second procedure would be coded as a revision, since we were not able to reliably identify the type of operation based on the available data.

Readmissions after primary and revision arthroplasty

We derived the costs of hospital readmissions after primary arthroplasty using PROMs/HES data. Our HES data extract included all hospital admissions for patients contained in the NHS PROMs data (i.e., who underwent primary or revision arthroplasty between April 2009 and October 2015). In a first step, we split the data into two datasets, one consisting of all “index” (i.e., primary or revision arthroplasty) episodes and one containing all other episodes. Then we matched the non-index admissions to the index episodes. If a hospital admission could be matched to more than one index episode, we matched the admission to the closest arthroplasty episode that occurred before the hospital admission. However, we excluded arthroplasty episodes from the match if the hospital admission referred to a different joint or side of the body than the arthroplasty episode.

To determine whether a matched hospital admission can be attributed to primary or revision arthroplasty, we applied the following set of criteria:

For peer review only - http://bmjopen.bmj.com/site/about/guidelines.xhtml

- The admission occurred within 30 days after primary arthroplasty.
- The admission had a primary diagnosis for hip or knee arthritis.
- The admission had a procedure code referring to the hip or knee joint.
- The admission had a diagnosis code associated with infections of the skin, the joint or the prosthesis.

These criteria were developed based on discussions with a clinical registrar and a clinical coding manager. The detailed diagnosis and procedure codes are available in the online supplementary file. Any admission that met at least one of the criteria was retained as a relevant episode.

Finally, we aggregated all readmissions by year from primary arthroplasty to create an annual measure of readmissions for each observed primary arthroplasty procedure. We assumed that readmissions were zero for those patients known to be alive with no recorded admissions in any given year. The resulting dataset contains observations for all patients (including those without hospital readmissions) and all years for up to six years following primary arthroplasty. Patients were considered to be observed either until the end of the study period (October 2015), or until their death if they died in hospital. Deaths outside of the hospital were not observed in the data. We analysed readmission costs in the first 12 months after primary arthroplasty separately from readmission costs more than 12 months after surgery. We also conducted a separate analysis of readmission costs in the year of revision surgery and readmission costs after revision surgery.

Ambulatory costs after primary arthroplasty and costs before primary arthroplasty

Ambulatory costs included outpatient and community costs related to primary arthroplasty, e.g., GP visits, nurse visits, physiotherapy, outpatient appointments at a hospital or visits to the "Accidents & Emergency" department. We excluded costs such as medication, nursing home care, personal care, and equipment or home modifications, due to a lack of data.

For THR, data on ambulatory costs in the first 12 months after primary arthroplasty were obtained from the COASt study, but ambulatory costs more than 12 months after primary hip arthroplasty could not be analysed due to a lack of data. For TKR, we used data on ambulatory costs from the KAT trial for up to 12 years following primary knee arthroplasty, and derived separate estimates for costs in the first 12 months after TKR and costs more than 12 months after TKR. Data on nurse visits and visits to the "Accidents & Emergency" department were not available from KAT, which means that ambulatory costs after TKR might be slightly underestimated. For TKR, we also conducted a separate analysis of ambulatory costs in the year of revision surgery and in subsequent years.

Finally, we analysed costs in the year before primary arthroplasty based on data from COASt. We included both ambulatory costs (as above) and hospital admissions.

Costing

We chose 2014 as the reference year for costs, since this is the most recent year which is completely observed in our data extract.

For peer review only - http://bmjopen.bmj.com/site/about/guidelines.xhtml

Hospital admissions in the HES data were valued using the NHS Local Payment Grouper 2014/15[25] and prices from the 2014/15 National Schedule.[26] For readmissions, we distinguished between elective and non-elective admissions and used the respective prices. For those hospital readmissions that could not be costed (e.g., due to errors in the grouping process or missing data on length of stay) we imputed costs by using either the mean cost per bed-day for admissions in the same category (if data on length of stay was available), or (if data on length of stay was missing) by using the mean cost per admission that meet the same criteria as a relevant readmission episode. The imputed values are provided in Table B.1 of the supplementary file. We did not impute costs for primary and revision arthroplasty episodes and omitted observations that could not be costed.

Costs for ambulatory visits and hospital admissions in KAT and COASt were derived by attaching the relevant unit costs for 2014 (see Supplementary file Table B.1) to the number of visits/appointments.

For peer review only

Covariates

The OHS and OKS are PROMs, each consisting of 12 questions about pain and functional limitations specific to the joint. The total score varies between 0 and 48, with higher values indicating less pain and functional limitations.[27,28]

We also included age and sex in the regression models. When studying the impact of OHS/OKS on costs of primary arthroplasty, quality-of-life, costs before arthroplasty and costs in the first 12 months following arthroplasty, we included age at operation in the regression models. When studying the impact of OHS/OKS on costs more than 12 months after primary arthroplasty, we also considered including current age instead of age at operation as well as a time trend into the model. While the inclusion of further covariates (e.g., body mass index or the American Society of Anesthesiologists (ASA) physical status classification) would be desirable, this information was unfortunately not available in the NHS PROMs and HES datasets.

STATISTICAL METHODS

The regression models presented in this study focus on predictive accuracy rather than causal inference. To this end, we developed a model selection procedure to systematically identify the best performing model from a range of candidate models. We began by conducting an exploratory data analysis for each outcome of interest to identify a range of candidate models. We visually inspected the distribution of each outcome as well as plots of the outcome against OHS/OKS and age. Based on these figures (available upon request), we identified candidates for *(i)* the statistical model class (e.g., ordinary least squares (OLS) or Tobit regression), *(ii)* the functional form for OHS/OKS (e.g., linear or quadratic trends), *(iii)* the functional form of the age trend, and *(iv)* functional forms for the time trend (where applicable). In principle, any combination of these four components identifies a valid

For peer review only - http://bmjopen.bmj.com/site/about/guidelines.xhtml

candidate model. However, in practice it was not feasible to test all possible candidate models against each other. Therefore, we selected our final model in four steps:

- In the first step, we selected the most appropriate model class from among the candidate models. All models included a linear trend for OHS/OKS, a linear trend for age and an indicator for sex of the patient and models on longitudinal data also included a linear trend for time since arthroplasty. Depending on the exploratory data analysis, we considered linear regression models, generalized linear models (GLM), two-part and Tobit regression models for censored data.
- Where applicable, we then chose the functional form for the time trend using the model class identified in the first step.
- In the second step, we chose the functional form for OHS/OKS. We used the model class identified in the first step and included a linear trend for age as well as an indicator for sex of the patient. We considered polynomials (e.g., linear, quadratic or cubic polynomials) as well as linear splines, where the spline points were identified in the exploratory data analysis.
- The third step consisted of choosing the functional form for age. We used the model class identified in the first step and the functional form for OHS/OKS from the second step. We considered polynomial trends, linear splines as well as age categories, depending on the exploratory data analysis. We also considered whether excluding age would improve prediction accuracy. When modelling costs more than 12 months after primary arthroplasty, we first examined whether age at operation or current age resulted in higher predictive accuracy using linear trends before choosing the functional form for the chosen age variable.
- In the fourth and last step, we evaluated an alternative model selected on the third step that omitted the indicator for sex of the patient, to assess whether this improved prediction accuracy.

We used mean squared error (MSE) as our criterion for model selection and used a 10-fold cross-validation process to avoid overfitting. We randomly divided our sample into 10 slices. For each of the steps outlined above, we estimated the candidate models using nine of these data slices, then predicted the outcomes for the slice left out of the estimation, and finally calculated the mean squared error of the prediction. This was repeated 10 times, so that each data slice was used once for prediction (i.e., internal validation) and the squared error was calculated for each candidate model and for every observation in the sample. Then, we averaged across all patients (in all 10 slices) to estimate the MSE for each candidate model, and chose the model with the lowest MSE. Finally, we re-estimated the model using the whole dataset to obtain our final estimates.

Further details on the candidate models considered for each outcome are provided alongside the results. All regression models were estimated using Stata version 14 (StataCorp, College Station TX). We conducted a complete-case analysis, omitting observations with missing data on any of the variables of interest. For the model selection procedure, we omitted all observations with missing data on outcome, OHS or OKS, age as well as sex to ensure that all candidate models were evaluated based on the same dataset. For the final estimation, we

For peer review only - http://bmjopen.bmj.com/site/about/guidelines.xhtml

only omitted observations with missing data on any of the included variables. With the exception of the models estimated on COASt data, very few observations were excluded due to missing information on covariates (Table 1).

RESULTS

Quality-of-life

Table 2 shows the estimated model for the association between OHS/OKS and EQ-5D utility six months after primary arthroplasty. The large sample size allowed us to estimate a very flexible model using a non-linear functional form and high-order polynomials.

Panel A and B in Figure 1 below show the relationship between OHS/OKS and pre-operative as well as post-operative EQ-5D utility. The lines show the fitted values, while the grey areas represent 95% confidence intervals. It should be noted that the fitted values are derived from the estimation sample and therefore are averaged across the age and sex distribution in the sample. This implies that for some of the smaller samples shown in subsequent figures, the depicted relationship may not be smooth despite the use of continuous trends due to, e.g., correlations between OHS/OKS score and age.

Both pre-operative and post-operative quality-of-life were higher for patients with high OHS/OKS scores (Figure 1A-B). However, the relationship is clearly non-linear. Pre-operative quality-of-life increased considerably with OHS/OKS for scores between 0 and 25, beyond which the increase in quality-of-life by OHS/OKS was much smaller. In contrast, post-operative quality-of-life increased strongly with OHS/OKS for scores between 0 and 10, after which there were only small differences in quality-of-life by OHS/OKS. As a consequence, the health gain from TJR (i.e., the difference between the two curves) was largest for patients with low scores. However, the models predicted that post-operative EQ-5D utility was higher than pre-operative utility for all patients with OHS $\leq$46 and those with OKS $\leq$44, which indicates that all patients with lower scores benefit from TJR.

There was some variation in post-operative EQ-5D by age and sex: post-operative utility was highest for THR patients aged 60 to 69, and increased with age for patients aged 30 to 60 and then decreased with age for patients aged above 70 (Table 2). Post-operative utility for TKR patients increased with age for all patients. Compared to men, women had higher pre-operative utility before TKR ($p<0.001$), but lower post-operative utility after THR ($p<0.001$). Sex was excluded in the models for utility before THR and utility after TKR.

For peer review only - http://bmjopen.bmj.com/site/about/guidelines.xhtml

Table 2: The impact of OHS/OKS on EQ-5D-3L six months after TJR

	Hip arthroplasty			Knee arthroplasty		
Model	*Tobit model censored at -0.594 and 1*			*Tobit model censored at -0.594 and 1*		
**Variable**	**Coefficient**		**Robust S.E.**	**Coefficient**		**Robust S.E.**
OHS/OKS						
Linear term	5.839E-02	***	4.337E-03	5.725E-02	***	2.482E-03
Quadratic term	-3.312E-03	***	5.400E-04	-2.356E-03	***	2.008E-04
Cubic term	1.182E-04	***	2.990E-05	5.390E-05	***	6.600E-06
Quartic term	-2.110E-06	**	7.520E-07	-4.660E-07	***	7.530E-08
Quintic term	1.460E-08	*	6.980E-09	-	-	-
Age (reference category: 50-54)						
Age <30	-9.039E-02	***	1.717E-02	-1.467E-01	*	6.181E-02
Age 30-39	-4.423E-02	***	1.047E-02	-4.303E-02		2.371E-02
Age 40-49	-1.240E-02	*	6.128E-03	-2.316E-02	**	6.787E-03
Age 55-59	3.204E-03		5.012E-03	1.138E-02	*	4.470E-03
Age 60-64	2.120E-02	***	4.592E-03	4.654E-02	***	4.094E-03
Age 65-69	3.235E-02	***	4.422E-03	8.938E-02	***	3.980E-03
Age 70-74	3.831E-03		4.377E-03	8.991E-02	***	3.966E-03
Age 75-79	-2.073E-02	***	4.400E-03	8.561E-02	***	4.000E-03
Age 80-84	-4.607E-02	***	4.629E-03	8.467E-02	***	4.208E-03
Age 85-89	-6.786E-02	***	5.558E-03	7.706E-02	***	5.099E-03
Age 90-94	-6.984E-02	***	1.027E-02	7.987E-02	***	1.066E-02
Age >=95	-1.072E-01	**	4.130E-02	9.356E-02		4.858E-02
Female sex	-3.099E-02	***	1.691E-03	-	-	-
Constant term	4.451E-01	***	1.297E-02	1.741E-01	***	1.105E-02
σ (Tobit term)	3.442E-01	-	8.904E-04	2.997E-01	-	7.109E-04
N	208,344			223,836		
Data	PROMs-HES			PROMs-HES		
Candidate models						
Model class	OLS, Tobit, two-part (Logit-OLS)			OLS, Tobit, two-part (Logit-OLS)		
OHS/OKS trend	Quadratic, cubic, quartic, quintic, log-linear			Quadratic, cubic, quartic, quintic, log-linear		
Age trend	Linear, quadratic, cubic, quartic, quintic, five-year bands, exclusion			Linear, quadratic, cubic, quartic, quintic, linear spline (65), five-year bands, exclusion		
Sex	yes/no			yes/no		

Sources: NHS PROMs and HES data, 2009-2015; own calculations.* p<0.05; ** p<0.01; *** p<0.001. Details on how to calculate predictions are provided in the online supplementary file.

For peer review only

For peer review only - http://bmjopen.bmj.com/site/about/guidelines.xhtml

Costs of primary and revision arthroplasty

The cost of primary arthroplasty decreased with OHS/OKS for scores between 0 and 10 (Figure 1C and 1D). For scores between 10 and 40, the costs remained approximately constant. For scores above 40, the predicted costs changed only slightly, while the uncertainty around these estimates increased considerably, since less than 0.4% of the patients in the sample have a score above 40. Costs for primary THR were higher for women than for men ($p<0.001$), while the costs of TKR were lower for women ($p=0.026$).

Panels E and F in Figure 1 show the relationship between the cost of revision arthroplasty and pre-operative OHS/OKS score. Due to the smaller number of revisions, the uncertainty around these estimates is larger than for the costs of primary surgery, but the estimates suggest that the costs of THR revision surgery increased with OHS ($p=0.016$), while the costs of TKR revision surgery remained constant ($p=0.837$ on the linear trend). The cost of revision arthroplasty was lower for older patients. Costs for THR revision surgery were lower for women than for men ($p<0.001$).

Costs in the year before TJR and in the first 12 months after surgery

Costs in the year before THR decreased significantly with increasing OHS ($p=0.003$), while costs before TKR varied little between OKS values of 0 and 20, before decreasing thereafter (Figure 2A and 2B) However, for both THR and TKR the curves are relatively flat. Costs decreased (albeit insignificantly) with age for both THR and TKR. Since there was only one patient with an OHS of 33, who was relatively young, the age effect produced an apparent spike in costs for an OHS score of 33.

Panels C and D of Figure 2 show the estimated association between OHS/OKS and ambulatory costs in the first 12 months after TJR. Overall, ambulatory costs after THR were considerably lower than costs after TKR. A sensitivity analysis estimating ambulatory costs for TKR patients in COASt confirmed this finding, suggesting that this trend reflects differences between joints rather than between datasets. Ambulatory costs decreased with increasing OHS/OKS scores. While the graph for THR seems to suggest that costs are lower for patients with very low and very high scores, it should be noted that these estimates are based on smaller numbers and are subject to considerable uncertainty.

Costs of hospital readmissions

In line with the findings for the costs of primary surgery, the cost of hospital readmissions in the first 12 months after TJR decreased steeply with OHS/OKS for scores between 0 and 10, while for scores above 10 the curve was almost flat (Figure 2E and 2F). Both for THR and TKR the costs were lower for women than for men ($p<0.001$ for both models).

Figure 3 shows the relationship between OHS/OKS and the costs of hospital readmissions more than 12 months after primary arthroplasty. The costs of hospital readmissions per year for patients without revision surgery are shown in Panels A and B. For both THR and TKR the costs decreased with OHS/OKS ($p<0.001$). For THR, the costs in year 2 (i.e., between 12

For peer review only - http://bmjopen.bmj.com/site/about/guidelines.xhtml

and 24 months after THR) were significantly (p<0.001) higher than in subsequent years. For TKR, the time trend indicates that readmission costs decreased over time (p<0.001).

Hospital readmission costs in the year of revision surgery did not have a statistically significant association with OHS/OKS scores measured before primary arthroplasty (Figures 2C and 2D).

Finally, the costs of hospital readmissions per year for patients who had undergone revision surgery >12 months previously (Panels E and F) were higher for patients who had low OHS/OKS scores before primary arthroplasty (p<0.05). However, the difference between patients with very low and very high scores was relatively small.

DISCUSSION

This study estimated the impact of pre-operative OHS and OKS on EQ-5D-3L utility and costs for patients undergoing THR or TKR. The results showed that there is considerable heterogeneity between patient groups, and that pre-operative OHS and OKS scores are systematically associated with both costs and quality-of-life before and after TJR. In particular, we found that while quality-of-life increases after TJR for patients at almost all scores, the improvements were larger for patients with lower scores. However, costs also tended to be higher for patients with low pre-operative scores. Therefore, delaying TJR surgery until patients' symptoms deteriorate may not be appropriate. While the increase in quality-of-life is greater for patients with worse symptoms, such patients also continue to have lower EQ-5D utility after TJR than patients with fewer symptoms. Moreover, delaying TJR surgery will incur considerable costs, since patients incur significant costs before surgery and TJR surgery is more costly for patients with low scores.

We also found that the difference between pre- and post-operative quality-of-life reduced as OHS/OKS increased above 10 or 20. This suggests that there could be a threshold score beyond which TJR might not be considered beneficial. However, identifying this threshold is beyond the scope of this paper and left to future research.

Two previous studies on the cost-effectiveness of TKR and THR, respectively, found that while OHS/OKS were systematically associated with both costs and outcomes of TJR, both procedures were nonetheless cost-effective for almost all patients.[3,4] Similarly, our results indicate that quality-of-life improved for almost all patients undergoing TKR and THR. While the improvement was largest for patients with low scores, we also found that costs tended to be higher for patients with low scores.

A recent study concluded that, in the US, access to TKR could be restricted to patients with more severe symptoms.[14] Similar to our findings, they reported that changes in quality-of-life were lower for patients with fewer pre-operative symptoms (measured by SF-12 physical score). However, in their study TKR had minimal effects on quality-of-life on average (SF-6D utility increased by 0.008). Our findings contrast strongly with this, indicating that at least in the UK, TJR is associated with sizable increases in EQ-5D-3L utility (average change of

For peer review only - http://bmjopen.bmj.com/site/about/guidelines.xhtml

0.32 for TKR and 0.45 for THR) for almost all patients currently undergoing surgery. This discrepancy may be due to differences between EQ-5D-3L and SF-6D, or differences between our sample covering almost all UK knee replacement procedures and a small US sample of early osteoarthritis patients.

Since OHS and OKS are already being used to determine eligibility for TJR,[6] evidence on their impact on the cost, efficacy and cost-effectiveness of primary arthroplasty is required to inform guidelines. The results presented in this study provide evidence on the association between preoperative scores and important measures of costs and quality-of-life. Moreover, the models presented here can be used as inputs in future model-based studies assessing the cost, efficacy and/or cost-effectiveness of THR and TKR, and in particular to explore heterogeneity between patient subgroups.

In this study, we used data from a range of different sources, including clinical trials and comprehensive administrative databases. The sample sizes in this study are up to 1000 times larger than those used in previous studies.[3,4,14] Crucially, this reduces the uncertainty around our estimates for patients with very high scores – i.e., patients who are most likely to be affected by rationing. Our data also had a longer follow-up duration for TKR than previous studies (up to 12 years compared with five years in previous studies[4,14]). Moreover, we employed a systematic model selection procedure to select the most appropriate statistical model from a range of different candidate models.

Nevertheless, there are several limitations to our study. KAT and COASt are markedly smaller than NHS PROMs-HES, and consequently models estimated on those datasets have more uncertainty and are relatively parsimonious. On the other hand, these datasets allowed us to estimate models for a larger range of relevant outcomes. Using data from different sources also makes it more difficult to draw conclusions on total costs or cost-effectiveness. This requires long-term modelling work, for which the models presented in this study could serve as parameters. We conducted a complete-case analysis, since the use of multiple imputation would have further increased the complexity of our analysis. However, there is little reason to expect that missing data would be systematically related to the shape of the relationship between OHS/OKS and costs or quality-of-life. While we studied a wide range of outcome variables, we were not able to examine revision rates or long-term outcomes for THR due to a lack of appropriate data. We also focused on a limited range of covariates (OHS/OKS, age and sex). We did not investigate the impact of other covariates that may affect our outcomes and are used in clinical decision-making (e.g., body mass index) due to a lack of data. While we tested a wide range of candidate models, our model selection was done sequentially, and therefore it is possible that there are interactions between model components that were not considered in this study. Our model selection process was developed to assess prediction accuracy, and therefore we did not consider statistical significance or confounding when selecting our models.

In summary, our results show that pre-operative OHS and OKS are strongly associated with the costs and quality-of-life of patients undergoing TJR. However, further research is needed

For peer review only - http://bmjopen.bmj.com/site/about/guidelines.xhtml

to estimate cost-effectiveness, and to determine the impact on revision rates and long-term outcomes for THR.

Acknowledgements

We would like to thank the ACHE investigators, user group and steering group for their role in the project.

We thank the KAT study group for providing access to their data for use in this project, as well as all of the researchers and participants involved in that study. We would like to thank all the participants of the COASt Study, Professor Nigel Arden and the COASt team for their time and dedication, and the NIHR for their funding support.

KAT was funded by the NIHR as an HTA (No. 95/10/01). COASt was funded by the National Institute for Health Research (NIHR) under its Programme Grants for Applied Research Programme (Reference Number RP-PG-0407-10064).

Data on NHS PROMs linked to HES APC data were reused with the permission of NHS Digital, copyright 2015, with all rights reserved.

The views and opinions expressed in this paper are those of the authors and do not necessarily reflect those of the HTA programme, NIHR, NHS, NHS Digital or the Department of Health.

We would also like to thank Chaudhry Shah and Joaquim Soares do Brito for their help with identifying the relevant codes for hospital readmissions used in the HES data.

Funding

This study was conducted as part of the project "Introducing Standardised And Evidence-Based Thresholds for Hip and Knee Replacement Surgery – The Arthroplasty Candidacy Help Engine (The ACHE Tool)", which was funded under the UK National Institute of Health Research (NIHR) Health Technologies Assessment (HTA) Programme (HTA 11/63/01). Visit the HTA programme website (www.hta.ac.uk) for further project information. The funder had no involvement in: the collection, analysis, and interpretation of data; in the writing of the report; and in the decision to submit the article for publication. The authors had full access to the data (including statistical reports and tables), can take responsibility for the integrity of the data and the accuracy of the data analysis and are responsible for submitting this paper.

Competing interest statement

All authors have completed the Unified Competing Interest form at www.icmje.org/coi_disclosure.pdf (available on request from the corresponding author). All authors received a grant from NIHR funding the current work. HD reports personal fees from Halyard Health, outside the submitted work. NA reports grants from Bioiberica, personal fees from Bioventus, personal fees from ESCEO, personal fees from Flexion, personal fees from Freshfields Bruckhaus Deringer, personal fees from Merck, personal fees from Regeneron, outside the submitted work. The other authors declare that there are no relevant conflicts of interests.

For peer review only - http://bmjopen.bmj.com/site/about/guidelines.xhtml

Copyright/licence

The Corresponding Author has the right to grant on behalf of all authors and does grant on behalf of all authors, a worldwide licence to the Publishers and its licensees in perpetuity, in all forms, formats and media (whether known now or created in the future), to i) publish, reproduce, distribute, display and store the Contribution, ii) translate the Contribution into other languages, create adaptations, reprints, include within collections and create summaries, extracts and/or, abstracts of the Contribution, iii) create any other derivative work(s) based on the Contribution, iv) to exploit all subsidiary rights in the Contribution, v) the inclusion of electronic links from the Contribution to third party material where-ever it may be located; and, vi) licence any third party to do any or all of the above.

Authorship statement

AP, DB, HD, AG and NA conceived, designed and conducted the ACHE study. PE, HD and AG designed and conducted the regression analysis reported in this manuscript. PE and HD analysed the data. PE drafted the manuscript. All authors edited the manuscript for important intellectual content and approved the final version.

Transparency declaration

The manuscript's guarantor (PE) affirms that the manuscript is an honest, accurate, and transparent account of the study being reported; that no important aspects of the study have been omitted; and that any discrepancies from the study as planned (and, if relevant, registered) have been explained.

Data sharing

The statistical code as well as variance-covariance matrices for the analyses reported in this study are available from the authors upon request. PROMs/HES data are available from NHS Digital (http://content.digital.nhs.uk/dars); enquiries about access to the KAT data should be directed to kat@abdn.ac.uk; enquiries about access to the COASt data should be directed to coast.study@ndorms.ox.ac.uk.

For peer review only

References

1 Gandhi R, Dhotar H, Davey JR, *et al.* Predicting the Longer-term Outcomes of Total Hip Replacement. *J Rheumatol* 2010;**37**:2573–7. doi:10.3899/jrheum.100149

2 Judge A, Batra RN, Thomas GE, *et al.* Body mass index is not a clinically meaningful predictor of patient reported outcomes of primary hip replacement surgery: prospective cohort study. *Osteoarthritis Cartilage* 2014;**22**:431–9. doi:10.1016/j.joca.2013.12.018

3 Fordham R, Skinner J, Wang X, *et al.* The economic benefit of hip replacement: a 5-year follow-up of costs and outcomes in the Exeter Primary Outcomes Study. *BMJ Open* 2012;**2**:e000752. doi:10.1136/bmjopen-2011-000752

4 Dakin H, Gray A, Fitzpatrick R, *et al.* Rationing of total knee replacement: a cost-effectiveness analysis on a large trial data set. *BMJ Open* 2012;**2**:e000332. doi:10.1136/bmjopen-2011-000332

5 Gandhi R, Dhotar H, Razak F, *et al.* Predicting the longer term outcomes of total knee arthroplasty. *The Knee* 2010;**17**:15–8. doi:10.1016/j.knee.2009.06.003

6 The Royal College of Surgeons of England. Is access to surgery a postcode lottery? Published Online First: 2014.https://www.rcseng.ac.uk/-/media/files/rcs/news-and-events/media-centre/is-access-to-surgery-a-postcode-lottery.pdf?la=en (accessed 8 Nov 2016).

7 Coastal West Sussex Clinical Commissioning Group. Low Priority Procedures (LPPs) and Procedures with Restrictions (OPRs), Commissioning for Clinical Effectiveness, NICE Interventional Procedures, version 2.10. Published Online First: 2015.https://www.coastalwestsussexccg.nhs.uk/domains/coastal-west-sussex-ccg.org.uk/local/media/documents/misc/Version_2.10_Low_Priority_Procedures_and_Other_Procedures_with_Restrictions_2015.pdf (accessed 8 Nov 2016).

8 Harrogate and Rural District Clinical Commissioning Group. Clinical thresholds: Hip and knee arthroplasty for osteoarthritis (only). Published Online First: 2014.http://www.harrogateandruraldistrictccg.nhs.uk/data/uploads/rss2/hip-and-knee-arthroplasty.pdf (accessed 8 Nov 2016).

9 Scarborough and Ryedale Clinical Commissioning Group. Hip replacement pathway. Published Online First: 2015.http://www.scarboroughryedaleccg.nhs.uk/data/uploads/rss2/orthopaedics/hip-replacement-march-2015.pdf (accessed 8 Nov 2016).

10 Worcestershire NHS. Commissioning Policy for Musculoskeletal Surgical Interventions. Published Online First: 2010.www.worcestershire.nhs.uk/EasySiteWeb/GatewayLink.aspx?alId=5553 (accessed 8 Nov 2016).

11 Losina E, Walensky RP, Kessler CL, *et al.* Cost-effectiveness of Total Knee Arthroplasty in the United States. *Arch Intern Med* 2009;**169**:1113–22. doi:10.1001/archinternmed.2009.136

For peer review only - http://bmjopen.bmj.com/site/about/guidelines.xhtml

12 Rissanen P, Aro S, Sintonen H, *et al.* Costs and cost-effectiveness in hip and knee replacements. A prospective study. *Int J Technol Assess Health Care* 1997;**13**:575–88.

13 Cushnaghan J, Bennett J, Reading I, *et al.* Long-term outcome following total knee arthroplasty: a controlled longitudinal study. *Ann Rheum Dis* 2009;**68**:642–7. doi:10.1136/ard.2008.093229

14 Ferket BS, Feldman Z, Zhou J, *et al.* Impact of total knee replacement practice: cost effectiveness analysis of data from the Osteoarthritis Initiative. *BMJ* 2017;:j1131. doi:10.1136/bmj.j1131

15 Williams DP, Price AJ, Beard DJ, *et al.* The effects of age on patient-reported outcome measures in total knee replacements. *Bone Jt J* 2013;**95–B**:38–44. doi:10.1302/0301-620X.95B1.28061

16 Judge A, Arden NK, Batra RN, *et al.* The association of patient characteristics and surgical variables on symptoms of pain and function over 5 years following primary hip-replacement surgery: a prospective cohort study. *BMJ Open* 2013;**3**:e002453. doi:10.1136/bmjopen-2012-002453

17 Murray DW, MacLennan GS, Breeman S, *et al.* A randomised controlled trial of the clinical effectiveness and cost-effectiveness of different knee prostheses: the Knee Arthroplasty Trial (KAT). *Health Technol Assess Winch Engl* 2014;**18**:1–235, vii–viii. doi:10.3310/hta18190

18 Arden N, Altman D, Beard D, *et al.* Lower limb arthroplasty: can we predict outcome and failure and is it cost effective? *Health Technol Assess* 2016;**(in press)**.

19 Herbert A, Wijlaars L, Zylbersztejn A, *et al.* Data Resource Profile: Hospital Episode Statistics Admitted Patient Care (HES APC). *Int J Epidemiol* doi:10.1093/ije/dyx015

20 Judge A, Cooper C, Williams S, *et al.* Patient-reported outcomes one year after primary hip replacement in a European Collaborative Cohort. *Arthritis Care Res* 2010;**62**:480–8. doi:10.1002/acr.20038

21 Marques EM, Blom AW, Lenguerrand E, *et al.* Local anaesthetic wound infiltration in addition to standard anaesthetic regimen in total hip and knee replacement: long-term cost-effectiveness analyses alongside the APEX randomised controlled trials. *BMC Med* 2015;**13**:151. doi:10.1186/s12916-015-0389-1

22 Wylde V, Blom AW, Bolink S, *et al.* Assessing function in patients undergoing joint replacement: a study protocol for a cohort study. *BMC Musculoskelet Disord* 2012;**13**:220. doi:10.1186/1471-2474-13-220

23 Feng Y, Parkin D, Devlin NJ. Assessing the performance of the EQ-VAS in the NHS PROMs programme. *Qual Life Res* 2014;**23**:977–89. doi:10.1007/s11136-013-0537-z

24 Dolan P. Modeling Valuations for EuroQol Health States. *Med Care* 1997;**35**:1095–108.

25 The National Casemix Office. HRG4 Grouper Reference Manual Payment 14/15. content.digital.nhs.uk/article/3938/HRG4-201415-Payment-Grouper?tabid=1 (accessed 10 Jun 2015).

For peer review only - http://bmjopen.bmj.com/site/about/guidelines.xhtml

26 Department of Health. Payment by Results in the NHS: tariff for 2014 to 2015: 2014- 15 tariff information spreadsheet. Published Online First: 2014.https://www.gov.uk/government/uploads/system/uploads/attachment_data/file/300551/Annex _5A_National_Prices.xlsx (accessed 19 Oct 2015).

27 Dawson J, Fitzpatrick R, Carr A, *et al.* Questionnaire on the Perceptions of Patients About Total Hip Replacement. *J Bone Jt Surg Br* 1996;**78–B**:185–90.

28 Dawson J, Fitzpatrick R, Murray D, *et al.* Questionnaire on the perceptions of patients about total knee replacement. *J Bone Jt Surg Br* 1998;**80–B**:63–9.

For peer review only

Figure legends

Figure 1

Sources: NHS PROMs and HES data, 2009-2015; own calculations. Costs are measured in 2014 Pound Sterling (£). Lines show predicted values averaged over all observations in the sample with a given OHS/OKS. The grey areas show 95% confidence intervals obtained from 1,000 bootstrap replications.

Figure 2

Sources: NHS PROMs, HES, KAT and COASt data, 2009-2015; own calculations. Costs are measured in 2014 Pound Sterling (£). Lines show predicted values averaged over all observations in the sample with a given OHS/OKS. The grey areas show 95% confidence intervals obtained from 1,000 bootstrap replications.

Figure 3

Sources: NHS PROMs and HES data, 2009-2015; own calculations. Costs are measured in 2014 Pound Sterling (£). Lines show predicted values averaged over all observations in the sample with a given OHS/OKS. The grey areas show 95% confidence intervals obtained from 1,000 bootstrap replications.

For peer review only

For peer review only - http://bmjopen.bmj.com/site/about/guidelines.xhtml

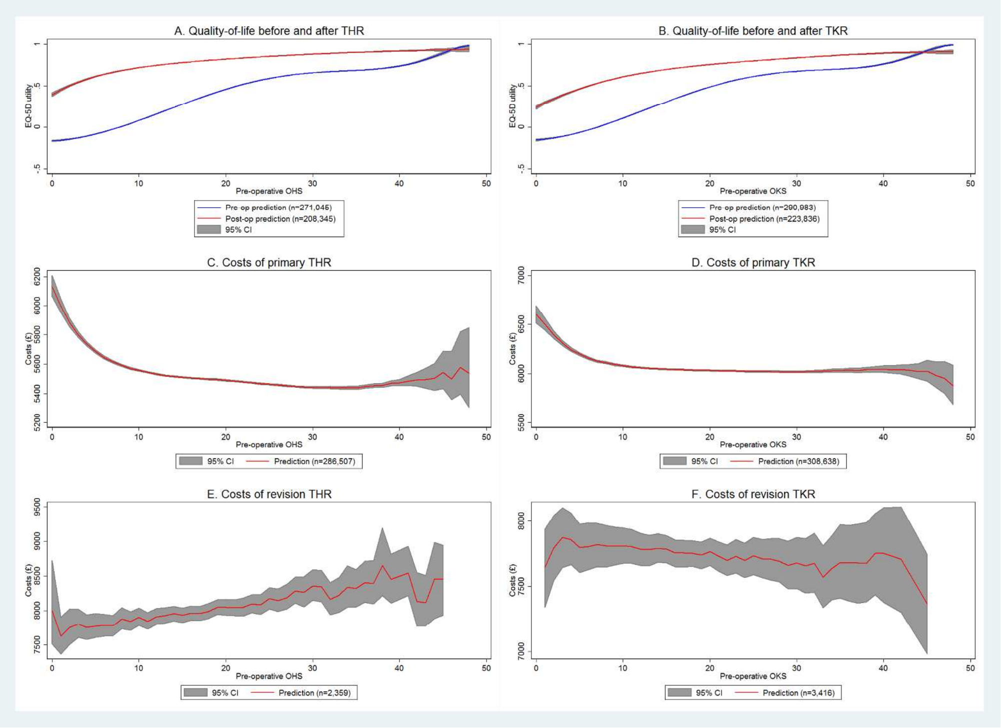

Sources: NHS PROMs and HES data, 2009-2015; own calculations. Costs are measured in 2014 Pound Sterling (£). Lines show predicted values averaged over all observations in the sample with a given OHS/OKS. The grey areas show 95% confidence intervals obtained from 1,000 bootstrap replications.

846x615mm (72 x 72 DPI)

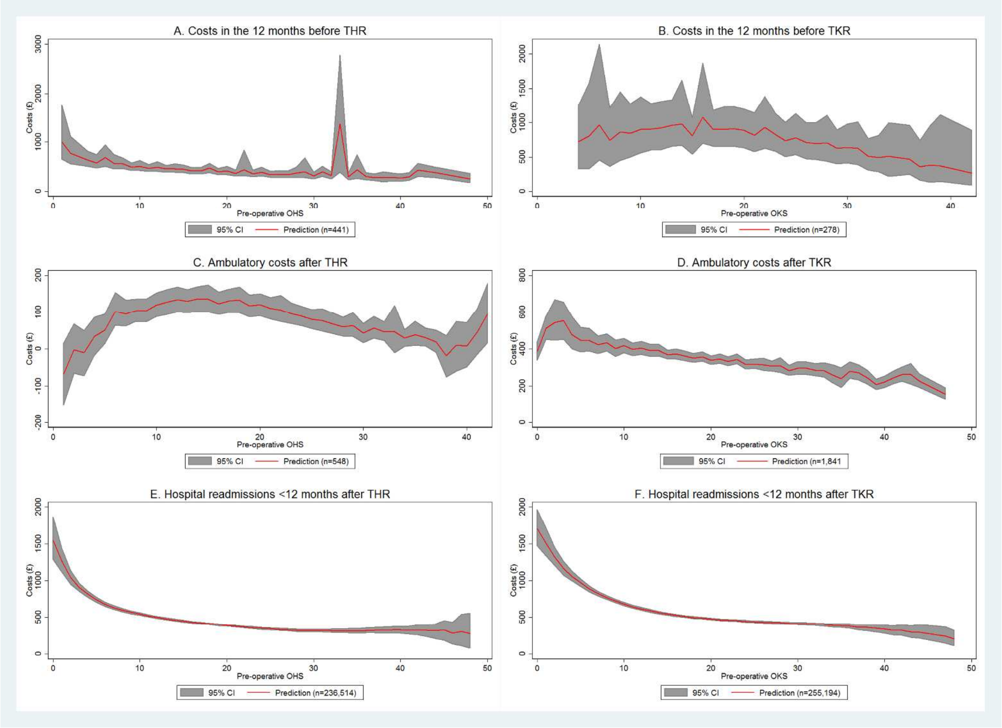

Sources: NHS PROMs, HES, KAT and COASt data, 2009-2015; own calculations. Costs are measured in 2014 Pound Sterling (£). Lines show predicted values averaged over all observations in the sample with a given OHS/OKS. The grey areas show 95% confidence intervals obtained from 1,000 bootstrap replications.

846x615mm (72 x 72 DPI)

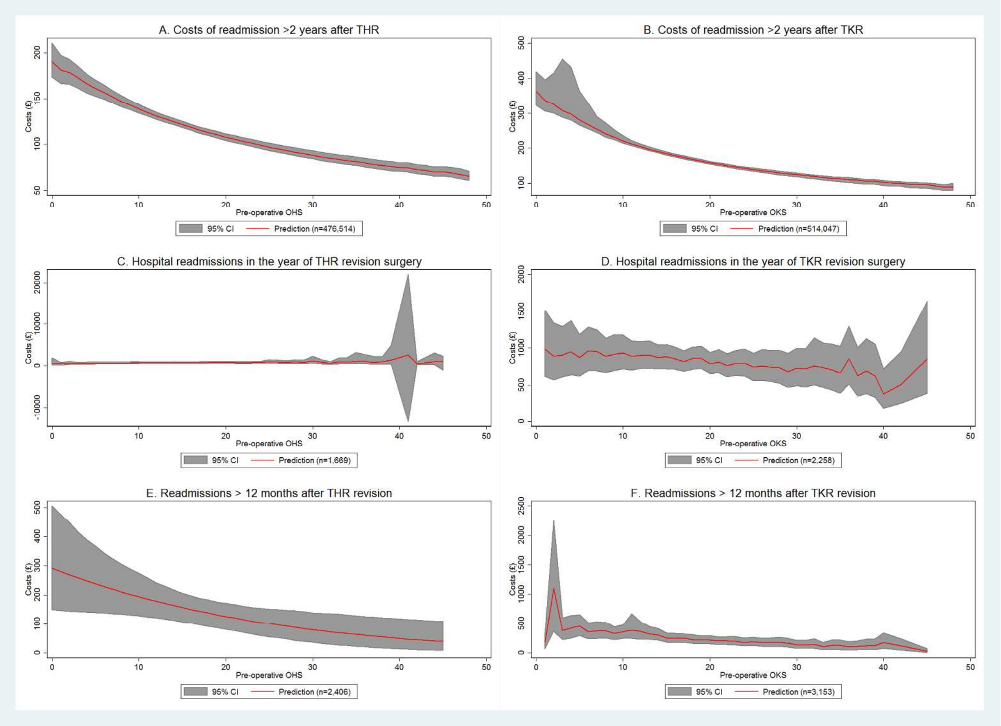

Sources: NHS PROMs and HES data, 2009-2015; own calculations. Costs are measured in 2014 Pound Sterling (£). Lines show predicted values averaged over all observations in the sample with a given OHS/OKS. The grey areas show 95% confidence intervals obtained from 1,000 bootstrap replications.

846x615mm (72 x 72 DPI)

Appendix

A. Diagnoses and procedures codes used to define hospital readmissions

We used ICD-10 diagnosis codes and OPCS 4 procedure codes to determine whether a hospital admission was related to arthroplasty. Specifically, we used the following criteria:

- A hospital admission occurred within 30 days after the primary arthroplasty episode. This criterion was applied regardless of any specific diagnosis or procedure codes.
- The primary diagnosis (i.e., the first recorded ICD-10 diagnosis code) was M17.X (knee arthritis) for a knee replacement episode, or M16.X (hip arthritis) for a hip replacement episode.
- One of the recorded procedure codes referred to the hip or knee joint. The OPCS 4 codes Z843, Z761 or Z756 were used for hip replacement episodes, and the codes Z846, Z765, Z845. Z844, Z774 or Z787 were used for knee replacement episodes.
- The admission had a diagnosis code associated with infections of the skin, the joint or the prosthesis. We used the following ICD-10 codes for this purpose:
    - M25.X with Z96.6 – "Other joint disorder, not elsewhere classified" and "Presence of orthopedic joint implant"
    - T81.X with either Z96.6 or Y83.1 – "Complications of procedures, not elsewhere classified" and "Presence of orthopedic joint implant" or "Surgical operation with implant of artificial internal device […]"
    - T84.X – "Complications of internal orthopedic prosthetic devices, implants and grafts"
    - M96.X – "Intraoperative and postprocedural complications and disorders of musculoskeletal system, not elsewhere classified"
    - M00.X with Z96.6 – "Pyogenic arthritis" and "Presence of orthopedic joint implant"
    - M86.X with Z96.6 – "Osteomyelitis" and "Presence of orthopedic joint implant".

For peer review only

B. Unit costs

Table B.1 Unit costs applied to all primary datasets providing resource use data

Healthcare resource	Units	Unit cost	Reference and details
GP surgery visit	Per visit	£46	PSSRU 2014 page 195[1]: Cost of a surgery patient contact lasting 11.7 minutes was £46 including direct care staff costs and qualifications. A clinic patient contact lasting 17.2 minutes costs £67 including direct care staff costs and qualifications.
GP Practice nurse visit	Per visit	£13.69	PSSRU 2014 page 192: £53 per hour of face-to-face contact including qualifications (£44/hour excluding qualifications). Average surgery consultation for a GP practice nurse lasts 15.5 minutes, giving a consultation cost of £14
Community physiotherapist at clinic/GP surgery	Per visit	£51	NHS reference costs – PSSRU 2014 page 179 states that the mean cost for a one-to-one contact in physiotherapy services in 2013/2014 was £51. PSSRU estimates the unit cost per hour in 2013/2014 to be £32 without qualifications or £36 with qualifications (page 179)
Community physiotherapist – at home	Per visit	£51	For simplicity, applied the same cost as physiotherapy at GP surgery/clinic
Hospital physiotherapist	Per visit	£34	NHS reference costs – PSSRU 2014 page 235 states that the mean average cost for a non-consultant led (not admitted) follow-up physiotherapy attendance in 2013/14 was £34 (IQR £28-£38). PSSRU 2014 estimates the hourly cost of a hospital physiotherapist to be £37/hour including qualifications and £33 excluding qualifications
Physiotherapy – average	Per visit	£42.50	We assumed that 50% of physiotherapy visits are in hospital and 0% are in the community.

For peer review only

**Healthcare resource**	**Units**	**Unit cost**	**Reference and details**
Outpatient visit – orthopaedics – Follow-up visit	Per visit	£71	Payment by results tariff [2] Tariff information spreadsheet 2013-14: outpatient attendances- WF01A Follow Up Attendance - Single Professional Or WF02A Follow Up Attendance - Multi-Professional (both of these codes are the same price). Follow-up attendance is used here, since patients are assumed to have already attended clinic for their joint replacement
A&E attendance – not admitted	Per visit	£81.26	National schedule of reference costs [3] weighted average of all non-admitted accident and emergency attendances , weighted by the number of attendances.
Admission to hospital related to hip arthroplasty (No data on length of stay)	Per admission	£2,662	Department of Health reference costs 2013-14. [3] This was calculated by taking a weighted average cost of all HRGs with "hip" within the currency description on the EL & NEL worksheets, weighting by the number of FCEs.
Admission to hospital related to knee arthroplasty (No data on length of stay)	Per admission	£3,907	Department of Health reference costs 2013-14 [3]. This was calculated by taking a weighted average cost of all HRGs with "knee" within the currency description on the EL & NEL worksheets, weighting by the number of FCEs.
Readmission to hospital within 30 days of hip or knee arthroplasty	Per bed-day	£395.83	Average costs per bed-day for readmissions in PROMs/HES within 30 days of hip or knee arthroplasty
Readmission to hospital within 30 days of hip or knee arthroplasty (No data on length of stay)	Per admission	£1,255	Average costs for readmissions in PROMs/HES within 30 days of hip or knee arthroplasty
Readmission to hospital with a diagnosis of hip/knee arthritis after hip/knee arthroplasty	Per bed-day	£1,107.25	Average costs per bed-day for readmissions in PROMs/HES with a diagnosis of hip/knee arthritis after hip/knee arthroplasty
Readmission to hospital with a diagnosis of	Per	£5,220	Average costs for readmissions in PROMs/HES with a diagnosis of

For peer review only

Healthcare resource	Units	Unit cost	Reference and details
hip/knee arthritis after hip/knee arthroplasty (No data on length of stay)	admission		hip/knee arthritis after hip/knee arthroplasty
Readmission to hospital for a hip/knee-specific procedure following hip/knee arthroplasty	Per bed-day	£551.33	Average costs per bed-day for readmissions in PROMs/HES for a hip/knee-specific procedure following hip/knee arthroplasty
Readmission to hospital for a hip/knee-specific procedure following hip/knee arthroplasty (No data on length of stay)	Per admission	£1,654	Average costs for readmissions in PROMs/HES for a hip/knee-specific procedure following hip/knee arthroplasty
Readmission to hospital with a diagnosis for infection following hip/knee arthroplasty	Per bed-day	£694.8	Average costs per bed-day for readmissions in PROMs/HES with a diagnosis for infection following hip/knee arthroplasty
Readmission to hospital with a diagnosis for infection following hip/knee arthroplasty (No data on length of stay)	Per admission	£1,876	Average costs for readmissions in PROMs/HES diagnosis for infection following hip/knee arthroplasty

References

[1] PSSRU | Unit Costs of Health and Social Care 2014 n.d. http://www.pssru.ac.uk/project-pages/unit-costs/2014/ (accessed June 14, 2017).
[2] Department of Health. Payment by Results in the NHS: tariff for 2014 to 2015: 2014- 15 tariff information spreadsheet. 2014.
[3] Department of Health. NHS reference costs 2013 to 2014 2014. https://www.gov.uk/government/publications/nhs-reference-costs-2013-to-2014 (accessed June 14, 2017).

For peer review only

C. Regression results

Table C.1: The impact of OHS/OKS on EQ-5D-3L before TJR

	**Hip arthroplasty**			**Knee arthroplasty**		
Model	*Tobit model censored at -0.594 and 1*			*Tobit model censored at -0.594 and 1*		
**Variable**	**Coefficient**		**Robust S.E.**	**Coefficient**		**Robust S.E.**
OHS/OKS						
Linear term	5.719E-03	**	2.040E-03	1.001E-02	***	2.403E-03
Quadratic term	2.385E-03	***	2.641E-04	2.150E-03	***	2.997E-04
Cubic term	-3.800E-05	*	1.500E-05	-3.140E-05		1.660E-05
Quartic term	-1.480E-06	***	3.850E-07	-1.650E-06	***	4.190E-07
Quintic term	3.010E-08	***	3.650E-09	3.240E-08	***	3.920E-09
Age						
Linear term	-8.742E-03	***	3.866E-03	-2.943E-02	**	1.046E-02
Quadratic term	3.742E-04		1.062E-04	7.750E-04	**	2.541E-04
Cubic term	-5.190E-06	***	1.240E-06	-8.360E-06	**	2.700E-06
Quartic term	2.340E-08		5.290E-09	3.270E-08	**	1.060E-08
Female sex	-	-	-	1.040E-02	***	8.351E-04
Constant term	-1.649E-01	**	5.079E-02	1.791E-01		1.592E-01
σ (Tobit term)	2.128E-01	-	3.022E-04	2.177E-01	-	2.895E-04
N	271,045			290,983		
Data	PROMs-HES			PROMs-HES		
Candidate models						
Model class	OLS, Tobit, two-part (Logit-OLS)			OLS, Tobit, two-part (Logit-OLS)		
OHS/OKS trend	Quadratic, cubic, quartic, quintic, log-linear			Quadratic, cubic, quartic, quintic, log-linear		
Age trend	Linear, quadratic, cubic, quartic, quintic, five-year bands, exclusion			Linear, quadratic, cubic, quartic, quintic, five-year bands, exclusion		
Sex	yes/no			yes/no		

Sources: NHS PROMs and HES data, 2009-2015; own calculations.* p<0.05; ** p<0.01; *** p<0.001. Details on how to calculate predictions are provided in Appendix D.

For peer review only

For peer review only - http://bmjopen.bmj.com/site/about/guidelines.xhtml

Table C.2: The impact of OHS/OKS on the costs of primary arthroplasty

	Hip arthroplasty			Knee arthroplasty		
Model	*gamma-GLM with inverse link function*			*gamma-GLM with inverse link function*		
**Variable**	**Coefficient**		**S.E.**	**Coefficient**		**SE**
OHS/OKS						
Linear term	3.100E-06	***	1.970E-07	2.670E-06	***	2.920E-07
Quadratic term	-2.580E-07	***	2.500E-08	-2.260E-07	***	3.580E-08
Cubic term	1.070E-08	***	1.400E-09	9.700E-09	***	1.960E-09
Quartic term	-2.080E-10	***	3.500E-11	-2.020E-10	***	4.870E-11
Quintic term	1.500E-12	***	3.220E-13	1.600E-12	***	4.470E-13
Age						
Linear term	-8.280E-07		1.220E-06	7.640E-07	***	6.090E-08
Quadratic term	4.310E-08		4.730E-08	-7.760E-09	***	4.460E-10
Cubic term	-8.870E-10		8.730E-10	-	-	-
Quartic term	8.530E-12		7.740E-12	-	-	-
Quintic term	-3.510E-14		2.650E-14	-	-	-
Female sex	7.440E-07	***	9.180E-08	-2.470E-07	*	1.110E-07
Constant term	1.717E-04	***	1.190E-05	1.377E-04	***	2.210E-06
N	286,507			308,638		
Data	PROMs-HES			PROMs-HES		
Candidate models						
Model class	OLS, gamma-GLM with inverse link, gamma-GLM with log-link, Gaussian-GLM with log-link			OLS, gamma-GLM with inverse link, gamma-GLM with log-link, Gaussian-GLM with log-link		
OHS/OKS trend	Linear, cubic, quartic, quintic, sextic, log-linear			Linear, cubic, quartic, quintic, sextic, log-linear		
Age trend	Linear, quadratic, cubic, quartic, quintic, five-year bands, exclusion			Linear, quadratic, cubic, five-year bands, ten-year bands, exclusion		
Sex	yes/no			yes/no		

Sources: NHS PROMs and HES data, 2009-2015; own calculations.* p<0.05; ** p<0.01; *** p<0.001. Details on how to calculate predictions are provided in Appendix D.

For peer review only

Table C.3: The impact of OHS/OKS on the costs of revision arthroplasty

	Hip arthroplasty			Knee arthroplasty		
Model	*gamma-GLM with inverse link function*			*OLS*		
**Variable**	**Coefficient**		**Clustered S.E.**	**Coefficient**		**Clustered S.E.**
			OHS/OKS			
Linear term	-2.370E-07	*	9.790E-08	-1.404E+00		6.816E+00
			Age			
Linear term	9.060E-07	*	3.500E-07	-2.101E+01	***	4.846E+00
Quadratic term	-6.620E-09	*	3.010E-09	-	-	-
Female sex	6.700E-06	***	1.570E-06	7.458E+01		9.551E+01
Constant term	9.570E-05	***	1.000E-05	9.111E+03	***	3.275E+02
N	2,359			3,416		
Data	PROMs-HES			PROMs-HES		
			Candidate models			
Model class	OLS, gamma-GLM with inverse link, gamma-GLM with log-link, Gaussian-GLM with log-link			OLS, gamma-GLM with inverse link, gamma-GLM with log-link, Gaussian-GLM with log-link		
OHS/OKS trend	Linear, cubic, log-linear			Linear, cubic, quartic, log-linear		
Age trend	Linear, quadratic, cubic, exclusion			Linear, quadratic, cubic, exclusion		
Sex	yes/no			yes/no		

Sources: NHS PROMs and HES data, 2009-2015; own calculations.* p<0.05; ** p<0.01; *** p<0.001. Details on how to calculate predictions are provided in Appendix D.

For peer review only

Table C.4: The impact of OHS/OKS on costs in the 12 months leading up to TJR

	Hip arthroplasty			Knee arthroplasty		
Model	*gamma-GLM with log-link*			*gamma-GLM with log-link*		
**Variable**	**Coefficient**		**S.E.**	**Coefficient**		**S.E.**
OHS/OKS						
Log-linear term	-3.485E-01	**	1.177E-01	-	-	-
Linear term	-	-	-	4.307E-02		7.466E-02
Quadratic term	-	-	-	-1.580E-03		1.741E-03
Age						
Linear term	-8.699E-02		4.441E-02	-2.131E-02		1.430E-02
Quadratic term	5.482E-04		3.489E-04	-	-	-
Female sex	-	-	-	-	-	-
Constant term	1.033E+01	***	1.429E+00	8.027E+00	***	1.302E+00
N	441			278		
Data	COASt			COASt		
Candidate models						
Model class	OLS, Tobit censored at zero, gamma-GLM with log-link, two-part (Logit + OLS/gamma-GLM with log-link/gamma-GLM with inverse link)			OLS, Tobit censored at zero, gamma-GLM with log-link, two-part (Logit + OLS/gamma-GLM with log-link/gamma-GLM with inverse link)		
OHS/OKS trend	Linear, quadratic, cubic, log-linear			Linear, quadratic, cubic		
Age trend	Linear, quadratic, cubic, 20-year bands, exclusion			Linear, quadratic, cubic, 20-year bands, exclusion		
Sex	yes/no			yes/no		

Sources: COASt data; own calculations.* p<0.05; ** p<0.01; *** p<0.001. Details on how to calculate predictions are provided in Appendix D. For observations with OHS=0, we set ln(OHS)=0.

For peer review only

Table C.5: The impact of OHS/OKS on ambulatory costs within 12 months after primary arthroplasty

	Hip arthroplasty			Knee arthroplasty		
Model	*OLS Regression*			*gamma-GLM with log-link*		
**Variable**	**Coefficient**		**Robust S.E.**	**Coefficient**		**Robust S.E.**
	OHS/OKS					
Linear term	2.881E+01	**	8.642E+00	-1.552E-02	***	3.408E-03
Quadratic term	-1.403E+00	**	4.400E-01	-	-	-
Cubic term	1.827E-02	**	6.815E-03	-	-	-
	Age					
Linear term	-2.670E+00	*	1.128E+00	-2.908E-02	***	3.060E-03
Female sex	-	-	-	-	-	-
Constant term	1.371E+02		9.342E+01	8.170E+00	***	2.203E-01
N	548			1,841		
Data	COASt			KAT		
	Candidate models					
Model class	OLS, Tobit censored at zero, gamma-GLM with inverse link, gamma-GLM with log-link, Gaussian-GLM with log-link, two-part (Logit + OLS/gamma-GLM with inverse link/gamma-GLM with log-link/Gaussian-GLM with log-link)			OLS, Tobit censored at zero, gamma-GLM with inverse link, gamma-GLM with log-link, Gaussian-GLM with log-link, two-part (Logit + OLS/gamma-GLM with inverse link/gamma-GLM with log-link/Gaussian-GLM with log-link)		
OHS/OKS trend	Linear, quadratic, cubic, linear spline (20)			Linear, cubic, linear spline (25)		
Age trend	Linear, quadratic, cubic, exclusion			Linear, quadratic, cubic, log-linear, exclusion		
Sex	yes/no			yes/no		

Sources: COASt and KAT data; own calculations.* p<0.05; ** p<0.01; *** p<0.001. Details on how to calculate predictions are provided in Appendix D.

For peer review only

Table C.6: The impact of OHS/OKS on the costs of hospital readmissions within 12 months after primary arthroplasty

	Hip arthroplasty			Knee arthroplasty		
Model	*Gaussian-GLM with log-link function*			*Gaussian-GLM with log-link function*		
**Variable**	**Coefficient**		**Clustered S.E.**	**Coefficient**		**Clustered S.E.**
OHS/OKS						
Linear term	-1.727E-01	***	3.719E-02	-1.177E-01	***	1.342E-02
Quadratic term	1.325E-02	**	5.060E-03	3.638E-03	***	7.422E-04
Cubic term	-6.122E-04	*	2.955E-04	-4.350E-05	***	1.230E-05
Quartic term	1.370E-05		7.640E-06	-	-	-
Quintic term	-1.140E-07		7.160E-08	-	-	-
Age						
Linear term	5.318E-03	**	1.671E-03	-8.114E-02	***	9.237E-03
Linear spline (age ≥70)	3.448E-02	***	3.360E-03	-	-	-
Quadratic term	-	-	-	7.103E-04	***	6.670E-05
Female sex	-1.785E-01	***	2.144E-02	-2.500E-01	***	2.070E-02
Constant term	6.788E+00	***	1.476E-01	9.687E+00	***	3.008E-01
N	236,514			255,194		
Data	PROMs-HES			PROMs-HES		
Candidate models						
Model class	OLS, Tobit censored at zero, two-part (Logit + OLS/gamma-GLM with inverse link/gamma-GLM with log-link/Gaussian-GLM with log-link), gamma-GLM with inverse link, gamma-GLM with log-link, Gaussian-GLM with log-link			OLS, Tobit censored at zero, two-part (Logit + OLS/gamma-GLM with inverse link/gamma-GLM with log-link/Gaussian-GLM with log-link), gamma-GLM with inverse link, gamma-GLM with log-link, Gaussian-GLM with log-link		
OHS/OKS trend	Linear spline (10), quadratic, cubic, quartic, quintic			Linear spline (10), quadratic, cubic, quartic, quintic		
Age trend	Linear spline (70), linear, quadratic, cubic, exclusion			Linear spline (70), linear, quadratic, cubic, exclusion		
Sex	yes/no			yes/no		

Sources: NHS PROMs and HES data, 2009-2015; own calculations.* p<0.05; ** p<0.01; *** p<0.001. Details on how to calculate predictions are provided in Appendix D. The linear spline term is set to zero for patients aged below 70. For patients above 70, the term equals (age in years -70).

For peer review only

Table C.7: The impact of OHS/OKS on the costs of hospital readmissions more than 12 months after primary arthroplasty

	Hip arthroplasty			Knee arthroplasty		
Model	*gamma-GLM with inverse link function*			*gamma-GLM with inverse link function*		
**Variable**	**Coefficient**		**Clustered S.E.**	**Coefficient**		**Clustered S.E.**
	OHS/OKS					
Linear term	1.998E-04	***	1.660E-05	1.655E-04	***	8.150E-06
	Current age					
Linear term	4.230E-05	***	9.000E-06	-	-	-
	Age at primary arthroplasty					
Linear term	-	-	-	4.600E-05	***	5.300E-06
	Time trend					
Linear term	-	-	-	1.227E-03	***	6.870E-05
Year 2 since primary surgery	-2.446E-03	***	2.263E-04	-	-	-
Female sex	-	-	-	4.290E-04	***	1.138E-04
Constant term	3.432E-03	***	7.112E-04	-3.965E-03	***	3.662E-04
N	476,514			514,047		
Data	PROMs-HES			PROMs-HES		
	Candidate models					
Model class	OLS, Tobit censored at zero, two-part (Logit + OLS/gamma-GLM with inverse link/gamma-GLM with log-link/Gaussian-GLM with log-link), gamma-GLM with inverse link, gamma-GLM with log-link, Gaussian-GLM with log-link			OLS, Tobit censored at zero, two-part (Logit + OLS/gamma-GLM with inverse link/gamma-GLM with log-link/Gaussian-GLM with log-link), gamma-GLM with inverse link, gamma-GLM with log-link, Gaussian-GLM with log-link		
Year since primary surgery (time trend)	Linear, quadratic, binary indicator for year 2, binary indicators for all years, exclusion			Linear, quadratic, binary indicator for year 2, binary indicators for all years, exclusion		
OHS/OKS trend	Linear, quadratic, cubic, quartic, quintic, log-linear			Linear, quadratic, cubic, quartic, quintic, log-linear		
Age trend	Age at operation vs. current age; for current age: Linear, quadratic, cubic, five-year age bands, exclusion			Age at operation vs. current age; for age at operation: Linear, quadratic, cubic, quartic, quintic, five-year age bands, exclusion		
Sex	yes/no			yes/no		

Sources: NHS PROMs and HES data, 2009-2015; own calculations.* p<0.05; ** p<0.01; *** p<0.001. Details on how to calculate predictions are provided in Appendix D. "Year 2 since primary surgery" is a binary indicator. "Year 1" refers to the first 12 months after primary surgery.

For peer review only

Table C.8: The impact of OHS/OKS on the costs of hospital readmissions during the year of revision surgery

	Hip arthroplasty			Knee arthroplasty		
Model	*Two-part model: Logit + gamma-GLM with inverse link*			*Two-part model: Logit + gamma-GLM with log-link*		
**Variable**	**Coefficient**		**Clustered S.E.**	**Coefficient**		**Clustered S.E.**
**Part 1: Logit model**						
OHS/OKS						
Linear term	8.407E-03		1.093E-02	3.790E-03		8.559E-03
Age at primary arthroplasty						
Linear term	9.589E-02	*	4.418E-02	5.140E-04		6.716E-03
Quadratic term	-8.234E-04	*	3.622E-04	-	-	-
Time trend						
Linear term	-	-	-	-2.488E+00		1.438E+00
Quadratic term	-	-	-	6.157E-01		3.933E-01
Cubic term	-	-	-	-4.586E-02		3.340E-02
Year 2 since primary surgery	3.153E-01		1.675E-01	-	-	-
Female sex	1.724E-01		1.624E-01	2.386E-01		1.235E-01
Constant term	-8.202E-01		1.353E+00	4.622E+00	**	1.699E+00
**Part 2: gamma-GLM**						
OHS/OKS						
Linear term	-1.340E-06		9.040E-07	-1.291E-02		7.915E-03
Age at primary arthroplasty						
Linear term	-9.550E-07		5.400E-06	1.018E-02		5.999E-03
Quadratic term	-4.800E-09		4.080E-08	-	-	-
Time trend						
Linear term	-	-	-	-3.169E-01		1.725E+00
Quadratic term	-	-	-	1.474E-01		4.819E-01
Cubic term	-	-	-	-1.556E-02		4.192E-02
Year 2 since primary surgery	4.800E-05	**	1.760E-05	-	-	-
Female sex	5.210E-05	*	2.040E-05	-9.075E-02		1.228E-01
Constant term	2.078E-04		1.764E-04	8.352E+00	***	1.982E+00
N	1,669			2,258		
Data	PROMs-HES			PROMs-HES		

...continued on next page

For peer review only

	Candidate models	
Model class	OLS, Tobit censored at zero, two-part (Logit + OLS/gamma-GLM with inverse link/gamma-GLM with log-link/Gaussian-GLM with log-link), gamma-GLM with inverse link, gamma-GLM with log-link, Gaussian-GLM with log-link	OLS, Tobit censored at zero, two-part (Logit + OLS/gamma-GLM with inverse link/gamma-GLM with log-link/Gaussian-GLM with log-link), gamma-GLM with inverse link, gamma-GLM with log-link, Gaussian-GLM with log-link
Year since primary surgery (time trend)	Binary indicator for year 2, exclusion	Linear, quadratic, cubic, exclusion
OHS/OKS trend	Linear, quadratic, cubic	Linear, quadratic, cubic
Age trend	Age at operation vs. current age; for age at operation: Linear, quadratic, cubic, five-year age bands, exclusion	Age at operation vs. current age; for age at operation: Linear, quadratic, cubic, five-year age bands, exclusion
Sex	yes/no	yes/no

Sources: NHS PROMs and HES data, 2009-2015; own calculations.* p<0.05; ** p<0.01; *** p<0.001. Details on how to calculate predictions are provided in Appendix D. "Year 2 since primary surgery" is a binary indicator. "Year 1" refers to the first 12 months after primary surgery.

For peer review only

Table C.9: The impact of OHS/OKS on the costs of hospital readmissions for patient with a previous revision

	Hip arthroplasty			Knee arthroplasty		
Model	*Tobit model censored at zero*			*Tobit model censored at zero*		
**Variable**	**Coefficient**		**Clustered S.E.**	**Coefficient**		**Clustered S.E.**
			OHS/OKS			
Linear term	-2.424E+02	*	1.074E+02	-1.972E+02	*	9.961E+01
			Current age			
Linear term	-	-	-	-1.172E+04	*	4.527E+03
Quadratic term	-	-	-	1.772E+02	*	7.058E+01
Cubic term	-	-	-	-8.911E-01	*	3.609E-01
			Time trend			
Linear term	-	-	-	-1.935E+03	***	4.973E+02
Constant term	-2.454E+04	***	3.657E+03	2.455E+05	**	9.386E+04
σ (Tobit term)	1.472E+04	-	1.859E+03	1.390E+04		2.139E+03
N	2,406			3,153		
Data	PROMs-HES			PROMs-HES		
			Candidate models			
Model class	OLS, Tobit censored at zero, two-part (Logit + OLS/gamma-GLM with inverse link/gamma-GLM with log-link/Gaussian-GLM with log-link), gamma-GLM with inverse link, gamma-GLM with log-link, Gaussian-GLM with log-link			OLS, Tobit censored at zero, two-part (Logit + OLS/gamma-GLM with inverse link/gamma-GLM with log-link/Gaussian-GLM with log-link), gamma-GLM with inverse link, gamma-GLM with log-link, Gaussian-GLM with log-link		
Year since primary surgery (time trend)	Linear, quadratic, exclusion			Linear, quadratic, cubic, exclusion		
OHS/OKS trend	Linear, quadratic, cubic			Linear, quadratic, cubic, quartic, quintic, log-linear		
Age trend	Age at operation vs. current age; for age at operation: linear, quadratic, cubic, five-year age bands, exclusion			Age at operation vs. current age; forcurrent age: linear, quadratic, cubic, exclusion		
Sex	yes/no			yes/no		

Sources: NHS PROMs and HES data, 2009-2015; own calculations.* p<0.05; ** p<0.01; *** p<0.001. Details on how to calculate predictions are provided in Appendix D.

For peer review only

Table C.10: The impact of OKS on EQ-5D-3L more than 12 months after TKR

	Knee arthroplasty		
Model	*OLS*		
**Variable**	**Coefficient**		**Clustered S.E.**
	OKS		
Log-linear term	1.776E-01	***	1.111E-02
	Current age		
Linear term	7.168E-03	***	1.541E-03
Linear spline term (70)	-8.851E-03	**	2.551E-03
Year since operation	-6.154E-03	***	1.417E-03
Interaction between year and linear spline term	-3.729E-04	*	1.669E-04
Previously revised	-1.598E-01	***	3.239E-02
Constant term	-2.372E-01	*	1.031E-01
N	15,414 (2,004 patients)		
Data	KAT		
Candidate models			
Model class	OLS with clustered standard errors, linear mixed models with random intercept and fixed slopes		
Time trend	Linear, quadratic		
OKS trend	Linear, quadratic, cubic, log-linear		
Age trend	For both age at operation and current age: Linear, quadratic, cubic, linear spline (70), exclusion		
Sex	yes/no		

Sources: KAT data; own calculations.* p<0.05; ** p<0.01; *** p<0.001. Details on how to calculate predictions are provided in Appendix D. The model was estimated on data in long-format (i.e., one observation per patient and year). The inclusion of the time trend was tested after selecting the model class and before choosing a trend for OKS. The linear spline term equals "(current age - 70)" for patients above 70, and "0" for those below. The interaction term is derived by multiplying the linear spline term with the year variable. We did not estimate a model for hips due to a lack of data.

For peer review only

Table C.11: The impact of OHS/OKS on EQ-5D-3L before revision surgery

	Hip arthroplasty			Knee arthroplasty		
Model	*Tobit model censored at -0.594 and 1*			*Tobit model censored at -0.594 and 1*		
**Variable**	**Coefficient**		**Clustered S.E.**	**Coefficient**		**Clustered S.E.**
	OHS/OKS					
Linear term	2.665E-02	***	4.188E-03	2.631E-02	**	8.271E-03
Quadratic term	-2.867E-04	**	1.041E-04	2.356E-04		4.623E-04
Cubic term	-	-	-	-1.510E-05		7.740E-06
	Age at primary operation					
Linear term	-	-	-	4.472E-03	***	9.990E-04
Linear spline term (70)	-	-	-	-5.607E-03		2.879E-03
	Age at revision surgery					
Linear term	2.123E-03	**	7.587E-04	-	-	-
Logarithm of year of revision	-	-	-	-3.601E-02		1.882E-02
Male sex	-	-	-	-2.358E-02		1.368E-02
Constant term	-1.444E-01	*	6.087E-02	-3.498E-01	***	7.534E-02
σ (Tobit term)	3.178E-01	-	4.755E-03	2.999E-01	-	3.676E-03
N	1,391 (1,331 patients)			2,227 (2,073 patients)		
Data	PROMs-HES			PROMs-HES		
	Candidate models					
Model class	OLS, Tobit			OLS, Tobit		
Year of revision (time trend)	Linear, exclusion			Linear, quadratic, cubic, log-linear, exclusion		
OHS/OKS trend	Linear, quadratic, cubic, log-linear			Linear, quadratic, cubic, quartic, log-linear, linear spline (30)		
Age trend	Age at operation vs. age at revision surgery; for age at revision surgery: linear, quadratic, cubic, exclusion			Age at operation vs. age at revision surgery; for age at operation: linear, quadratic, cubic, linear spline (70), exclusion		
Sex	yes/no			yes/no		

Sources: NHS PROMs and HES data, 2009-2015; own calculations.* p<0.05; ** p<0.01; *** p<0.001. Details on how to calculate predictions are provided in Appendix D. The linear spline term equals "(age at operation - 70)" for patients above 70, and "0" for those below.

For peer review only

Table C.12: The impact of OHS/OKS on EQ-5D-3L after revision surgery

Model	Hip arthroplasty: *Tobit model censored at -0.594 and 1*			Knee arthroplasty: *Tobit model censored at -0.594 and 1*		
**Variable**	**Coefficient**		**Clustered S.E.**	**Coefficient**		**Clustered S.E.**
	OHS/OKS					
Log-linear term	2.201E-01	***	2.580E-02	2.556E-01	***	1.950E-02
	Age at revision surgery					
Linear term	-8.181E-02		6.954E-02	1.352E-02	***	2.570E-03
Quadratic term	1.755E-03		1.099E-03	-	-	-
Cubic term	-1.120E-05		5.680E-06	-	-	-
Linear spline term (65)	-	-	-	-1.533E-02	***	3.747E-03
Male sex	-5.907E-02	*	2.696E-02	-8.874E-02	***	1.982E-02
Constant term	1.120E+00		1.445E+00	-9.426E-01	***	1.623E-01
σ (Tobit term)	3.633E-01	-	1.184E-02	3.441E-01	-	7.576E-03
N	884 (860 patients)			1,398 (1,331 patients)		
Data	PROMs-HES			PROMs-HES		
	Candidate models					
Model class	OLS, Tobit, two-part (Logit-OLS)			OLS, Tobit, two-part (Logit-OLS)		
Year of revision (time trend)	Linear, exclusion			Linear, quadratic, cubic, log-linear, exclusion		
OHS/OKS trend	Linear, quadratic, cubic, log-linear			Linear, quadratic, cubic, quartic, log-linear		
Age trend	Age at operation vs. age at revision surgery; for age at revision surgery: linear, quadratic, cubic, exclusion			Age at operation vs. age at revision surgery; for age at revision surgery: linear, quadratic, cubic, linear spline (65), exclusion		
Sex	yes/no			yes/no		

Sources: NHS PROMs and HES data, 2009-2015; own calculations.* p<0.05; ** p<0.01; *** p<0.001. Details on how to calculate predictions are provided in Appendix D. The linear spline term equals "(age at revision surgery - 65)" for patients above 65, and "0" for those below.

For peer review only

Table C.13: The impact of OKS on ambulatory costs more than 12 months after TKR

	Knee arthroplasty		
Model	*Gaussian-GLM with log-link function*		
**Variable**	**Coefficient**		**Clustered S.E.**
	OKS		
Linear term	1.125E-03		9.113E-04
Quadratic term	-5.597E-01	*	2.420E-01
	Age at primary operation		
Linear term	3.672E-01		2.467E-01
Quadratic term	-7.167E-03		4.043E-03
Cubic term	4.130E-05		2.150E-05
	Year since primary surgery (time trend)		
Linear term	-5.597E-01	*	2.420E-01
Quadratic term	5.295E-02		4.104E-02
Cubic term	-1.810E-03		2.111E-03
Female sex	-3.197E-01	*	1.363E-01
Constant term	1.212E+00		4.801E+00
N	13,271		
Data	KAT		
	Candidate models		
Model class	OLS, Tobit censored at zero, two-part (Logit + OLS/gamma-GLM with log-link), gamma-GLM with inverse link, gamma-GLM with log-link, Gaussian-GLM with log-link		
Time trend	Linear, quadratic, cubic trend, binary indicator for year 2, binary indicator for year 2 and a linear trend, exclusion		
OKS trend	Linear, quadratic, cubic		
Age trend	Age at operation vs. current age; for age at operation: Linear, quadratic, cubic, exclusion		
Sex	yes/no		

Sources: KAT data; own calculations.* p<0.05; ** p<0.01; *** p<0.001. Details on how to calculate predictions are provided in Appendix D. The model was estimated on data in long-format (i.e., one observation per patient and year). The inclusion of the time trend was tested after selecting the model class and before choosing a trend for OKS. We did not estimate a model for hips due to a lack of data.

For peer review only

Table C.14: The impact of OKS on ambulatory costs for patients in the year of revision surgery

	Knee arthroplasty		
Model	*gamma-GLM with log-link function*		
**Variable**	**Coefficient**		**Clustered S.E.**
	OKS		
Linear term	-3.271E-02		1.805E-02
	Age at primary operation		
Linear term	2.062E-01	*	8.868E-02
Quadratic term	-1.777E-03	**	6.774E-04
Constant term	1.145E+00		2.861E+00
N	88		
Data	KAT		
	Candidate models		
Model class	OLS, Tobit censored at zero, two-part (Logit + OLS/gamma-GLM with log-link/gamma-GLM with inverse link/Gaussian-GLM with log-link), gamma-GLM with inverse link, gamma-GLM with log-link, Gaussian-GLM with log-link		
OKS trend	Linear, quadratic, cubic		
Age trend	Linear, quadratic, cubic, exclusion		
Sex	yes/no		

Sources: KAT data; own calculations.* p<0.05; ** p<0.01; *** p<0.001. Details on how to calculate predictions are provided in Appendix D. The model was estimated on data in long-format (i.e., one observation per patient and year). We did not estimate a model for hips due to a lack of data.

For peer review only

Table C.15: The impact of OKS on ambulatory costs for patients with a previous revision

	Knee arthroplasty		
Model	*gamma-GLM with inverse link function*		
**Variable**	**Coefficient**		**Clustered S.E.**
	OKS		
Linear term	1.637E-03		9.185E-04
Quadratic term	-1.439E-04		7.930E-05
Cubic term	3.860E-06	*	1.890E-06
Female sex	1.726E-03		2.068E-03
Constant term	3.737E-04		1.859E-03
N	329		
Data	KAT		
	Candidate models		
Model class	OLS, Tobit censored at zero, two-part (Logit + OLS/gamma-GLM with log-link/gamma-GLM with inverse link/Gaussian-GLM with log-link), gamma-GLM with inverse link, gamma-GLM with log-link, Gaussian-GLM with log-link		
OKS trend	Linear, quadratic, cubic		
Age trend	Linear, quadratic, cubic, exclusion		
Sex	yes/no		

Sources: KAT data; own calculations.* p<0.05; ** p<0.01; *** p<0.001. Details on how to calculate predictions are provided in Appendix D. Notes: The model was estimated on data in long-format (i.e., one observation per patient and year). We did not estimate a model for hips due to a lack of data.

For peer review only

D. Note on calculating predictions

1) OLS models

The predicted value of the outcome variable, $\hat{y}$ is simply the linear predictor Xb, which is obtained by multiplying each variable with its estimated coefficient and then summing over all variables.

2) Gamma-GLM with an inverse link function

Predicted values for the outcome variable can be obtained as $\hat{y} = \frac{1}{Xb}$.

3) Gamma-GLM with a log-link function

Predicted values for the outcome variable can be obtained as $\hat{y} = e^{Xb}$.

4) Gaussian-GLM with a log-link functio

Predicted values for the outcome variable can be obtained as $\hat{y} = e^{Xb}$.

5) Tobit models censored at zero

The predicted value is given by $\hat{y} = -\Phi\left(\frac{Xb}{\sigma}\right)Xb + \sigma\phi\left(\frac{Xb}{\sigma}\right)$

where $\Phi(\cdot)$ denotes the cumulative distribution function of the standard normal distribution, and $\phi(\cdot)$ denotes the probability density function of the standard normal distribution.

6) Tobit models censored at -0.594 and 1

The predicted value is given by

$$\hat{y} = -0.594 * \Phi\left(\frac{-0.594 - Xb}{\sigma}\right) + 1 * \Phi\left(-\frac{1 - Xb}{\sigma}\right)$$

$$+\left(\Phi\left(\frac{1-Xb}{\sigma}\right) - \Phi\left(\frac{-0.594 - Xb}{\sigma}\right)\right) * \left(Xb - \sigma * \frac{\phi\left(\frac{1-Xb}{\sigma}\right) - \phi\left(\frac{-0.594-Xb}{\sigma}\right)}{\Phi\left(\frac{1-Xb}{\sigma}\right) - \Phi\left(\frac{-0.594-Xb}{\sigma}\right)}\right)$$

where $\Phi(\cdot)$ denotes the cumulative distribution function of the standard normal distribution, and $\phi(\cdot)$ denotes the probability density function of the standard normal distribution.

For peer review only

7) Two-part models

The predicted value from a two-part model is given by

$\hat{y} = \left(1 - P\left[y = 0 \mid X\right]\right) * E\left[y \mid y > 0, X\right]$.

$E\left[y \mid y > 0, X\right]$ is given by the predicted value in the second part of the model.

$P\left[y = 0 \mid X\right]$ is given as $P\left[y = 0 \mid X\right] = \frac{e^{Xb}}{1 + e^{Xb}}$ if the first part of the model is a logit model.

For peer review only

STROBE 2007 (v4) checklist of items to be included in reports of observational studies in epidemiology*
Checklist for cohort, case-control, and cross-sectional studies (combined)

Section/Topic	Item #	Recommendation	Reported on page #
**Title and abstract**	1	(*a*) Indicate the study's design with a commonly used term in the title or the abstract	p.2
		(*b*) Provide in the abstract an informative and balanced summary of what was done and what was found	p.2
**Introduction**			
Background/rationale	2	Explain the scientific background and rationale for the investigation being reported	p.3
Objectives	3	State specific objectives, including any pre-specified hypotheses	p.3
**Methods**			
Study design	4	Present key elements of study design early in the paper	pp. 4-12
Setting	5	Describe the setting, locations, and relevant dates, including periods of recruitment, exposure, follow-up, and data collection	p.4/5
Participants	6	(*a*) *Cohort study*—Give the eligibility criteria, and the sources and methods of selection of participants. Describe methods of follow-up<br>*Case-control study*—Give the eligibility criteria, and the sources and methods of case ascertainment and control selection. Give the rationale for the choice of cases and controls<br>*Cross-sectional study*—Give the eligibility criteria, and the sources and methods of selection of participants	p.5
		(*b*) *Cohort study*—For matched studies, give matching criteria and number of exposed and unexposed<br>*Case-control study*—For matched studies, give matching criteria and the number of controls per case	
Variables	7	Clearly define all outcomes, exposures, predictors, potential confounders, and effect modifiers. Give diagnostic criteria, if applicable	pp.8-10
Data sources/ measurement	8*	For each variable of interest, give sources of data and details of methods of assessment (measurement). Describe comparability of assessment methods if there is more than one group	*pp.8-10*
Bias	9	Describe any efforts to address potential sources of bias	p. 10/11
Study size	10	Explain how the study size was arrived at	NA
Quantitative variables	11	Explain how quantitative variables were handled in the analyses. If applicable, describe which groupings were chosen and why	pp.8-10
Statistical methods	12	(*a*) Describe all statistical methods, including those used to control for confounding	p.10/11
		(*b*) Describe any methods used to examine subgroups and interactions	p.10/11
		(*c*) Explain how missing data were addressed	p.10/11
		(*d*) *Cohort study*—If applicable, explain how loss to follow-up was addressed<br>*Case-control study*—If applicable, explain how matching of cases and controls was addressed	NA

For peer review only

		Cross-sectional study—If applicable, describe analytical methods taking account of sampling strategy	
		(*e*) Describe any sensitivity analyses	p.10/11
**Results**			
Participants	13*	(a) Report numbers of individuals at each stage of study—eg numbers potentially eligible, examined for eligibility, confirmed eligible, included in the study, completing follow-up, and analysed	p.4/5, Table 1 on [.6
		(b) Give reasons for non-participation at each stage	NA
		(c) Consider use of a flow diagram	NA
Descriptive data	14*	(a) Give characteristics of study participants (eg demographic, clinical, social) and information on exposures and potential confounders	NA
		(b) Indicate number of participants with missing data for each variable of interest	Table 1 on p.6
		(c) *Cohort study*—Summarise follow-up time (eg, average and total amount)	NA
Outcome data	15*	*Cohort study*—Report numbers of outcome events or summary measures over time	
		Case-control study—Report numbers in each exposure category, or summary measures of exposure	
		Cross-sectional study—Report numbers of outcome events or summary measures	NA
Main results	16	(*a*) Give unadjusted estimates and, if applicable, confounder-adjusted estimates and their precision (eg, 95% confidence interval). Make clear which confounders were adjusted for and why they were included	Table 2 on p.13 and supplementary material
		(*b*) Report category boundaries when continuous variables were categorized	Table 2 on p.13 and supplementary material
		(*c*) If relevant, consider translating estimates of relative risk into absolute risk for a meaningful time period	NA
Other analyses	17	Report other analyses done—eg analyses of subgroups and interactions, and sensitivity analyses	
**Discussion**			
Key results	18	Summarise key results with reference to study objectives	p. 15
Limitations	19	Discuss limitations of the study, taking into account sources of potential bias or imprecision. Discuss both direction and magnitude of any potential bias	p. 16
Interpretation	20	Give a cautious overall interpretation of results considering objectives, limitations, multiplicity of analyses, results from similar studies, and other relevant evidence	p. 15/16
Generalisability	21	Discuss the generalisability (external validity) of the study results	p. 15
**Other information**			
Funding	22	Give the source of funding and the role of the funders for the present study and, if applicable, for the original study on which the present article is based	p. 17

For peer review only

*Give information separately for cases and controls in case-control studies and, if applicable, for exposed and unexposed groups in cohort and cross-sectional studies.

Note: An Explanation and Elaboration article discusses each checklist item and gives methodological background and published examples of transparent reporting. The STROBE checklist is best used in conjunction with this article (freely available on the Web sites of PLoS Medicine at http://www.plosmedicine.org/, Annals of Internal Medicine at http://www.annals.org/, and Epidemiology at http://www.epidem.com/). Information on the STROBE Initiative is available at www.strobe-statement.org.

For peer review only

BMJ Open

Association between pre-operative Oxford hip and knee scores and costs and quality-of-life of patients undergoing primary total joint replacement in the NHS England: An observational study

Journal:	*BMJ Open*
Manuscript ID	bmjopen-2017-019477.R1
Article Type:	Research
Date Submitted by the Author:	07-Dec-2017
Complete List of Authors:	Eibich, Peter; University of Oxford, Health Economics Research Centre, Nuffield Department of Population Health; Max Planck Institute for Demographic Research<br>Dakin, Helen; University of Oxford, Health Economics Research Centre, Nuffield Department of Population Health<br>Price, Andrew; University of Oxford, Nuffield Department of Orthopaedics, Rheumatology and Musculoskeletal Sciences<br>Beard, David; University of Oxford, Nuffield Dept of Orthopaedics, Rheumatology and Musculoskeletal Sciences<br>Arden, Nigel; Nuffield Department of Orthopaedics, ;<br>Gray, Alastair; University of Oxford, Nuffield Department of Population Health
**Primary Subject Heading**:	Rheumatology
Secondary Subject Heading:	Health policy
Keywords:	hip replacement, knee replacement, costs, quality-of-life, Oxford Hip Score, Oxford Knee Score

SCHOLARONE™
Manuscripts

Association between pre-operative Oxford hip and knee scores and costs and quality-of-life of patients undergoing primary total joint replacement in the NHS England: An observational study

Peter Eibich[1,2], Helen Dakin[1], Andrew Price[3], David Beard[3], Nigel Arden[3,4] and Alastair M. Gray[1]

[1] Health Economics Research Centre, Nuffield Department of Population Health, University of Oxford, Oxford, UK

[2] Max Planck Institute for Demographic Research, Rostock, Germany

[3] Nuffield Department of Orthopaedics, Rheumatology and Musculoskeletal Sciences, University of Oxford, Oxford, UK

[4] Faculty of Medicine, University of Southampton, Southampton, UK

Running title: Associations between OHS/OKS and costs and quality-of-life

5702 words

3 tables

3 figures

Corresponding author:

Peter Eibich, Max Planck Institute for Demographic Research, Konrad-Zuse-Str. 1, 18057 Rostock, Germany. Tel.: +49 (0)381 2081-220. E-Mail: eibich@demogr.mpg.de.

For peer review only

For peer review only - http://bmjopen.bmj.com/site/about/guidelines.xhtml

Abstract

Objectives: To assess how costs and quality-of-life (measured by EQ-5D) before and after total hip and knee replacement (THR/TKR) vary with age, gender and pre-operative Oxford hip and knee scores (OHS/OKS).

Design: Regression analyses using prospectively collected data from clinical trials, cohort studies and administrative data bases.

Setting: UK secondary care.

Participants: Men and women undergoing primary THR or TKR. The Hospital Episode Statistics data linked to patient reported outcome measures included 602,176 patients undergoing hip or knee replacement who were followed up for up to six years. The Knee Arthroplasty Trial included 2,217 TKR patients who were followed up for 12 years. The Clinical Outcomes in Arthroplasty Study cohort included 806 THR and 484 TKR patients who were observed for one year.

Outcome measures: EQ-5D-3L quality-of-life before and after surgery, costs of primary arthroplasty, costs of revision arthroplasty and the costs of hospital readmissions and ambulatory costs in the year before and up to 12 years after joint replacement.

Results: Average post-operative utility for patients at the 5th percentile of the OHS/OKS distribution was 0.61/0.5 for THR/TKR, and 0.89/0.85 for patients at the 95th percentile. The difference between post- and pre-operative EQ-5D utility was highest for patients with pre-operative OHS/OKS lower than 10. However, post-operative EQ-5D utility was higher than pre-operative utility for all patients with OHS ≤46 and those with OKS ≤44. In contrast, costs were generally higher for patients with low pre-operative OHS/OKS than those with high OHS/OKS. For example, costs of hospital readmissions within 12 months after primary THR/TKR were £740/£888 for patients at the 5th percentile compared to £314/£404 at the 95th percentile of the OHS/OKS distribution.

Conclusions: Our findings suggest that the costs and quality-of-life changes associated with total joint replacement vary with symptoms and (to a lesser extent) with age and sex.

299 words

Keywords: Total joint replacement, hip replacement, knee replacement, costs, quality-of-life, Oxford Hip Score, Oxford Knee Score, regression model.

For peer review only - http://bmjopen.bmj.com/site/about/guidelines.xhtml

Strengths and limitations of this study

- This study used data from different sources, including clinical trials, cohort studies and administrative databases.
- The sample sizes are considerably larger than those used in previous studies.
- The optimal regression models were identified in a systematic model selection process designed to maximise predictive accuracy.
- Our largest dataset contained no information on potentially important variables such as body mass index.

For peer review only

For peer review only - http://bmjopen.bmj.com/site/about/guidelines.xhtml

INTRODUCTION

Total joint replacement (TJR) has been shown to greatly improve the quality-of-life of patients with osteoarthritis.[1–5] Moreover, previous studies have demonstrated that both total knee replacement (TKR) and total hip replacement (THR) are highly cost-effective.[3,4] Nevertheless, in the UK access to TJR surgery is frequently rationed based on patient characteristics (such as age and functional status),[6] since it is a non-urgent procedure with a high impact on healthcare budgets. In particular, the Oxford hip and knee scores (OHS/OKS) were used in certain regions to define threshold values (varying from 19 to 30) above which patients are not eligible for TJR.[7–10] This is based on the argument that patients with lower pre-operative functional status benefit more from TJR, but the evidence to support this is limited, and very few studies have examined how outcomes or costs of TJR differ by pre-operative patient characteristics.[1–5,11–16]

Most of these studies focused on demographic characteristics such as age, with only five studies exploring the impact of pre-operative function.[3,4,13,14,16] Cushnaghan et al.[13] examined associations between baseline physical functioning (measured by SF-36) and the long-term outcome of TKR (measured by the change in SF-36). Judge et al.[16] investigated associations between pre-operative OHS and post-operative OHS and concluded that all patients benefitted from substantially from THR. Only Dakin et al.[4] and Fordham et al.[3] investigated the impact of OKS and OHS on costs and outcomes of TKR and TJR, respectively, and both studies concluded that while health benefits and costs do vary with pre-operative OHS/OKS score, TJR improves quality-of-life for almost all patients.

One recent US study by Ferket et al.[14] concluded that TKR results in only minimal health gains for patients with good physical function (measured by the SF-12 physical score), and that, on economic grounds, access to TKR should be restricted to patients with severe symptoms; however this study was based on only 382 TKR patients. Other studies in this area have also relied on small datasets (e.g., 2,138 TKR[4] or 938 THR patients[3] followed for five years), which are likely to contain very few patients with low or high scores. The results from these studies are therefore subject to considerable uncertainty.

This study aimed to estimate the relationship between pre-operative patient characteristics (age, sex and OHS/OKS) and the costs and quality-of-life of patients undergoing TJR in England. We used administrative data from the NHS patient reported outcome measures (PROMs) programme linked to Hospital Episode Statistics (HES) data, alongside smaller datasets from the Knee Arthroplasty Trial (KAT) and the Clinical Outcomes in Arthroplasty Study (COASt) cohort study to obtain reliable estimates on a wide range of outcomes and patients, including for patients with very high OHS/OKS.

METHODS

Datasets

The study focused on patients undergoing elective primary hip or knee arthroplasty from three data sources –the KAT trial,[17] the COASt cohort study,[18] and data from the NHS PROMs programme linked to HES data.[19] These datasets were chosen from a wider range

For peer review only - http://bmjopen.bmj.com/site/about/guidelines.xhtml

of available candidate datasets[3,20–22] based on the availability of relevant outcome and baseline data as well as sample size considerations.

The NHS PROMs programme routinely collects data on all patients undergoing hip and knee replacement on the NHS in England.[23] Data are collected via questionnaires at baseline (typically the day of admission for surgery) and at minimum six months post-surgery. The dataset used in this study covered the period from April 2009 to October 2015. We linked these data to Admitted Patient Care data from HES,[19] which contains routine data on all patients treated in hospitals in the NHS England, with a particular focus on diagnoses and procedures. This yielded a sample of up to 602,176 patients who were followed up for up to six years. The data include patients undergoing primary joint replacement surgery other than TJR (e.g., hip resurfacing), since we were not able to reliably identify the type of operation from the available data.

KAT is a randomised controlled trial comparing different types of knee prosthesis.[17] We used data on EQ-5D utility and ambulatory costs following 2217 UK patients, who were followed up annually for up to 12 years after TKR.

COASt[18] is a prospective, dual-centre longitudinal cohort study recruiting patients across two hospitals: University Hospital Southampton NHS Foundation Trust and Nuffield Orthopaedic Centre (part of Oxford University Hospitals NHS Trust). COASt was established in 2010 and recruited patients placed on the waiting list for knee or hip replacement surgery. The datasets extracted for this study contained observations for 810 hip surgery patients and 858 patients undergoing knee surgery. After excluding patients who underwent procedures other than THR/TKR (e.g., partial knee replacement or hip resurfacing), the datasets contained 806 THR patients and 484 TKR patients. Data are collected prior to surgery as well as at six weeks and then annually for five years thereafter, although only the first year of data were used in this study.

Outcomes

Quality-of-life

Pre- and post-operative quality-of-life was measured using EQ-5D-3L, valued using the UK time trade-off tariff.[24] EQ-5D-3L health utility takes values between -0.594 and 1, with higher values representing higher quality-of-life. We chose to focus on EQ-5D, since it is the preferred measure of quality-of-life for economic evaluations submitted to the National Institute for Health and Care Excellence (NICE).

Linked NHS PROMs/HES data were used to estimate quality-of-life before surgery and six months after THR or TKR. Data from KAT were used to estimate annual changes in quality-of-life between six months and 12 years after TKR.

Costs

Primary and revision arthroplasty

For peer review only - http://bmjopen.bmj.com/site/about/guidelines.xhtml

We estimated the impact of OHS/OKS on the costs of primary and revision TJR using linked NHS PROMs/HES data. We identified HES observations related to primary or revision arthroplasty by linking NHS PROMs data to the HES database, and then excluded all observations marked as revisions in the corresponding NHS PROMs questionnaire. If a patient was observed to have the same procedure (THR or TKR) carried out at the same joint (right- or left-side) more than once, we assumed that the earliest observed procedure was a primary surgery episode, while all subsequent procedures were revision episodes. Similarly, we identified revisions as those episodes that were either marked as a revision in the NHS PROMs data, or where we observed an earlier procedure on the same joint in the HES data. This implies that for patients undergoing primary joint replacement other than TJR first and TJR on the same joint later, the first procedure would be coded as primary surgery and the second procedure would be coded as a revision, since we were not able to reliably identify the type of operation based on the available data.

Readmissions after primary and revision arthroplasty

We derived the costs of hospital readmissions after primary arthroplasty using PROMs/HES data. Our HES data extract included all hospital admissions for patients contained in the NHS PROMs data (i.e., who underwent primary or revision arthroplasty between April 2009 and October 2015). In a first step, we split the data into two datasets, one consisting of all “index” (i.e., primary or revision arthroplasty) episodes and one containing all other episodes. Then we matched the non-index admissions to the index episodes. If a hospital admission could be matched to more than one index episode, we matched the admission to the closest arthroplasty episode that occurred before the hospital admission. However, we excluded arthroplasty episodes from the match if the hospital admission referred to a different joint or side of the body than the arthroplasty episode.

To determine whether a matched hospital admission can be attributed to primary or revision arthroplasty, we applied the following set of criteria:

- The admission occurred within 30 days after primary arthroplasty.
- The admission had a primary diagnosis for hip or knee arthritis.
- The admission had a procedure code referring to the hip or knee joint.
- The admission had a diagnosis code associated with infections of the skin, the joint or the prosthesis.

These criteria were developed based on discussions with a clinical registrar and a clinical coding manager. The detailed diagnosis and procedure codes are available in the online supplementary file (section A). Any admission that met at least one of the criteria was retained as a relevant episode.

Finally, we aggregated all readmissions by year from primary arthroplasty to create an annual measure of readmissions for each observed primary arthroplasty procedure. We assumed that readmissions were zero for those patients known to be alive with no recorded admissions in any given year. The resulting dataset contains observations for all patients (including those without hospital readmissions) and all years for up to six years following primary

For peer review only - http://bmjopen.bmj.com/site/about/guidelines.xhtml

arthroplasty. Patients were considered to be observed either until the end of the study period (October 2015), or until their death if they died in hospital. Deaths outside of the hospital were not observed in the data. We analysed readmission costs in the first 12 months after primary arthroplasty separately from annual readmission costs between one and six years after surgery. We also conducted a separate analysis of readmission costs in the year of revision surgery and annual readmission costs after revision surgery.

Ambulatory costs after primary arthroplasty and costs before primary arthroplasty

Ambulatory costs included outpatient and community costs related to primary arthroplasty, e.g., GP visits, nurse visits, physiotherapy, outpatient appointments at a hospital or visits to the "Accidents & Emergency" department. We excluded costs such as medication, nursing home care, personal care, and equipment or home modifications, due to a lack of data.

For THR, data on ambulatory costs in the first 12 months after primary arthroplasty were obtained from the COASt study, but annual ambulatory costs more than 12 months after primary hip arthroplasty could not be analysed due to a lack of data. For TKR, we used data on annual ambulatory costs from the KAT trial for up to 12 years following primary knee arthroplasty, and derived separate estimates for costs in the first 12 months after TKR and annual costs between one and 12 years after TKR. Data on nurse visits and visits to the "Accidents & Emergency" department were not available from KAT, which means that ambulatory costs after TKR might be slightly underestimated. For TKR, we also conducted a separate analysis of ambulatory costs in the year of revision surgery and in subsequent years.

Finally, we analysed costs in the year before primary arthroplasty based on data from COASt. We included both ambulatory costs (as above) and hospital admissions.

Costing

We chose 2014 as the reference year for costs, since this is the most recent year which is completely observed in our data extract.

Hospital admissions in the HES data were valued using the NHS Local Payment Grouper 2014/15[25] and prices from the 2014/15 National Schedule.[26] For readmissions, we distinguished between elective and non-elective admissions and used the respective prices. For those hospital readmissions that could not be costed (e.g., due to errors in the grouping process or missing data on length of stay) we imputed costs by using either the mean cost per bed-day for admissions in the same category (if data on length of stay was available), or (if data on length of stay was missing) by using the mean cost per admission that meet the same criteria as a relevant readmission episode. The imputed values are provided in Table B.1 of the supplementary file. We did not impute costs for primary and revision arthroplasty episodes and omitted observations that could not be costed.

Costs for ambulatory visits and hospital admissions in KAT and COASt were derived by attaching the relevant unit costs for 2014 (see supplementary file Table B.1) to the number of visits/appointments.

For peer review only - http://bmjopen.bmj.com/site/about/guidelines.xhtml

Covariates

The OHS and OKS are PROMs, each consisting of 12 questions about pain and functional limitations specific to the joint. The total score varies between 0 and 48, with higher values indicating less pain and functional limitations.[27,28] We used OHS/OKS scores measured before primary arthroplasty in all models.

We also included age and sex in the regression models. When studying the impact of OHS/OKS on costs of primary arthroplasty, quality-of-life, costs before arthroplasty and costs in the first 12 months following arthroplasty, we included age at operation in the regression models. When studying the impact of OHS/OKS on costs more than 12 months after primary arthroplasty, we also considered including current age instead of age at operation as well as a time trend into the model. While the inclusion of further covariates (e.g., body mass index or the American Society of Anesthesiologists (ASA) physical status classification) would be desirable, this information was unfortunately not available in the NHS PROMs and HES datasets.

Descriptive statistics

Table 1 below provides an overview over all outcomes for which regression models were developed. Average post-operative EQ-5D utility was considerably higher than average pre-operative utility. Interestingly, average EQ-5D utility before revision surgery was similar to or even lower than average pre-operative utility. Average utility after revision was higher than average utility values before revision, however, it was lower than the average post-operative utility. On average, primary hip arthroplasty costed £5,522 and primary knee arthroplasty costed £6,053. Average cost in the 12 months before arthroplasty were £444 for THR and £836 for TKR. In the first 12 months after THR, average costs were £101 for ambulatory services and £455 for hospital readmissions. For TKR, average costs in the first 12 months after surgery were £361 for ambulatory services and £550 for hospital readmissions. Average pre-operative OHS scores varied between 16.8 and 18.7 (due to the different samples), while average pre-operative OKS scores varied between 16.9 and 19.4. For the sake of brevity, we discuss the modelling results for selected outcomes in this paper; the models for all other outcomes are provided in the appendix.

For peer review only - http://bmjopen.bmj.com/site/about/guidelines.xhtml

Table 1: Data sources and sample sizes

Variable of interest	Dataset	Mean ± Standard deviation (dependent variable)	Mean OHS	Final sample size	Missing covariates	Table	Figure
**A. Quality-of-life for THR**							
Post-operative EQ-5D utility	PROMs-HES	0.784 ± 0.247	18.1	208,345	0.96%	C.1	1.A
Pre-operative EQ-5D utility	PROMs-HES	0.334 ± 0.324	17.6	271,045	0.83%	C.2	1.A
EQ-5D utility before revision surgery	PROMs-HES	0.348 ± 0.341	17.2	1,391	1.21%	C.4	-
EQ-5D utility after revision surgery	PROMs-HES	0.648 ± 0.316	17.7	884	0.90%	C.5	-
**B. Costs (in £) for THR**							
Costs of primary arthroplasty	PROMs-HES	5,522 ± 744	17.5	286,507	1.12%	C.6	1.C
Costs of revision arthroplasty	PROMs-HES	7,994 ± 2,362	16.9	2,359	1.17%	C.7	1.E
Costs in the 12 months before arthroplasty	COASt	444 ± 679	18.7	441	16.00%	C.8	2.A
Ambulatory costs <12 months after primary arthroplasty	COASt	101 ± 244	18.5	548	16.72%	C.9	2.E
Readmissions <12 months after primary arthroplasty	PROMs-HES	455 ± 2,150	17.5	236,514	1.48%	C.10	2.C
Annual cost of readmissions between 1 and 6 years after primary arthrolasty	PROMs-HES	118 + 1,137	17.6	476,514	0.98%	C.11	3.A
Readmissions in the year of revision surgery	PROMs-HES	780 ± 3,507	16.8	1,669	1.07%	C.12	3.C
Annual cost of readmissions after revision surgery	PROMs-HES	148 ± 1,309	17.0	2,406	0.87%	C.13	3.E
**C. Quality-of-life for TKR**							
Post-operative EQ-5D utility	PROMs-HES	0.723 ± 0.259	19.0	223,836	0.96%	C.1	1.B
Pre-operative EQ-5D utility	PROMs-HES	0.391 ± 0.317	18.4	290,983	0.98%	C.2	1.B
EQ-5D utility between 1 and 12 years after primary arthroplasty	KAT	0.693 ± 0.276	18.4	15,414	0.00%	C.3	-
EQ-5D utility before revision surgery	PROMs-HES	0.291 ± 0.331	17.0	2,227	1.07%	C.4	-
EQ-5D utility after revision surgery	PROMs-HES	0.544 ± 0.340	17.7	1,398	0.99%	C.5	-
**D. Costs (in £) for TKR**							
Costs of primary arthroplasty	PROMs-HES	6,053 ± 1,097	18.4	308,638	1.21%	C.6	1.D

For peer review only - http://bmjopen.bmj.com/site/about/guidelines.xhtml

Costs of revision arthroplasty	PROMs-HES	7,759 ± 2,777	16.9	3,416	1.13%	C.7	1.F
Costs in the 12 months before arthroplasty	COASt	836 ± 2,131	19.4	278	10.03%	C.8	2.B
Ambulatory costs <12 months after primary arthroplasty	KAT	361 ± 400	18.3	1,841	3.21%	C.9	2.F
Readmissions <12 months after primary arthroplasty	PROMs-HES	550 ± 2,472	18.4	255,194	1.22%	C.10	2.C
Annual cost of readmissions between 1 and 6 years after primary arthrolasty	PROMs-HES	176 ± 1,311	18.4	514,047	1.18%	C.11	3.B
Readmissions in the year of revision surgery	PROMs-HES	844 ± 3,361	16.9	2,258	1.05%	C.12	3.D
Annual cost of readmissions after revision surgery	PROMs-HES	246 ± 1,877	16.9	3,153	1.10%	C.13	3.F
Annual ambulatory costs between 1 and 12 years after primary arthroplasty	KAT	29 ± 104	18.4	13,271	4.13%	C.14	-
Ambulatory costs in the year of revision surgery	KAT	578 ± 718	17.8	88	4.35%	C.15	-
Annual ambulatory costs after revision surgery	KAT	129 ± 235	18.1	329	3.24%	C.16	-

Notes: Tables C.1-C.16 are shown in the online supplementary file. "Missing covariates" shows the fraction of observations that were excluded from the final sample due to missing data on one of the covariates (incl. OHS/OKS) included into the model.
*: Model estimated on longitudinal data.

Statistical methods

The regression models presented in this study focus on predictive accuracy rather than causal inference. To this end, we developed a model selection procedure to systematically identify the best performing model from a range of candidate models. We began by conducting an exploratory data analysis for each outcome of interest to identify a range of candidate models. We visually inspected the distribution of each outcome as well as plots of the outcome against OHS/OKS and age. Based on these figures (available upon request), we identified candidates for *(i)* the statistical model class (e.g., ordinary least squares (OLS) or Tobit regression), *(ii)* the functional form for OHS/OKS (e.g., linear or quadratic trends), *(iii)* the functional form of the age trend, and *(iv)* functional forms for the time trend (where applicable). In principle, any combination of these four components identifies a valid candidate model. However, in practice it was not feasible to test all possible candidate models against each other. Therefore, we selected our final model in four steps:

- In the first step, we selected the most appropriate model class from among the candidate models. All models included a linear trend for OHS/OKS, a linear trend for age and an indicator for sex of the patient and models on longitudinal data also

For peer review only - http://bmjopen.bmj.com/site/about/guidelines.xhtml

included a linear trend for time since arthroplasty. Depending on the exploratory data analysis, we considered linear regression models, generalized linear models (GLM), two-part and Tobit regression models for censored data.

- Where applicable, we then chose the functional form for the time trend using the model class identified in the first step.
- In the second step, we chose the functional form for OHS/OKS. We used the model class identified in the first step and included a linear trend for age as well as an indicator for sex of the patient. We considered polynomials (e.g., linear, quadratic or cubic polynomials) as well as linear splines, where the spline points were identified in the exploratory data analysis.
- The third step consisted of choosing the functional form for age. We used the model class identified in the first step and the functional form for OHS/OKS from the second step. We considered polynomial trends, linear splines as well as age categories, depending on the exploratory data analysis. We also considered whether excluding age would improve prediction accuracy. When modelling costs more than 12 months after primary arthroplasty, we first examined whether age at operation or current age resulted in higher predictive accuracy using linear trends before choosing the functional form for the chosen age variable.
- In the fourth and last step, we evaluated an alternative model selected on the third step that omitted the indicator for sex of the patient, to assess whether this improved prediction accuracy.

We used mean squared error (MSE) as our criterion for model selection and used a 10-fold cross-validation process to avoid overfitting. We randomly divided our sample into 10 slices. For each of the steps outlined above, we estimated the candidate models using nine of these data slices, then predicted the outcomes for the slice left out of the estimation, and finally calculated the mean squared error of the prediction. This was repeated 10 times, so that each data slice was used once for prediction (i.e., internal validation) and the squared error was calculated for each candidate model and for every observation in the sample. Then, we averaged across all patients (in all 10 slices) to estimate the MSE for each candidate model, and chose the model with the lowest MSE. Finally, we re-estimated the model using the whole dataset to obtain our final estimates.

All regression models were estimated using Stata version 14 (StataCorp, College Station TX). We conducted a complete-case analysis, omitting observations with missing data on any of the variables of interest. For the model selection procedure, we omitted all observations with missing data on outcome, OHS or OKS, age as well as sex to ensure that all candidate models were evaluated based on the same dataset. For the final estimation, we only omitted observations with missing data on any of the included variables. With the exception of the models estimated on COASt data, very few observations were excluded due to missing information on covariates (Table 1). We visualise the estimated associations between OHS/OKS and quality-of-life as well as costs by plotting predicted costs and quality-of-life against OHS/OKS scores. We generated predictions using the estimation sample, i.e., for each OHS/OKS score we averaged predictions over the sample distributions of the other covariates. The full estimation results as well as the candidate models considered for each outcome are provided in section C of the supplementary file. Section D of the supplementary file provides details and examples for calculating predictions from the different models used in this study.

For peer review only - http://bmjopen.bmj.com/site/about/guidelines.xhtml

RESULTS

Quality-of-life

Panel A and B in Figure 1 below show the relationship between OHS/OKS and pre-operative as well as post-operative EQ-5D utility. The lines show the fitted values, while the grey areas represent 95% confidence intervals. Both pre-operative and post-operative quality-of-life were higher for patients with high OHS/OKS scores (Figure 1A-B). However, the relationship is clearly non-linear. Pre-operative quality-of-life increased considerably from -0.17/-0.16 for OHS/OKS of 0 to 0.59/0.61 for OHS/OKS scores of 25, beyond which the increase in quality-of-life by OHS/OKS was much smaller. In contrast, post-operative quality-of-life increased strongly from 0.39/0.24 for OHS/OKS of 0 to 0.72/0.61 for scores of 10, after which there were only small differences in quality-of-life by OHS/OKS. As a consequence, the health gain associated with TJR (i.e., the difference between the two curves) was largest for patients with low scores (0.69 for OHS of 5 and 0.53 for OKS of 6). While the models predicted that post-operative EQ-5D utility was higher than pre-operative utility for all patients with OHS ≤46 and those with OKS ≤44, the differences between post- and pre-operative utility were marginal (0.005 for OHS of 46 and 0.02 for OKS of 44).

There was some variation in post-operative EQ-5D by age and sex: post-operative utility was highest for THR patients aged 60 to 69, and increased with age for patients aged 30 to 60 and then decreased with age for patients aged above 70. Post-operative utility for TKR patients increased with age for all patients. Compared to men, women had higher pre-operative utility before TKR (p<0.001), but lower post-operative utility after THR (p<0.001). Sex was excluded in the models for utility before THR and utility after TKR.

Costs of primary and revision arthroplasty

The cost of primary arthroplasty decreased with OHS/OKS for scores between 0 (£6,132 for THR and £6,603 for TKR on average) and 10 (£5,559 for THR and £6,080 for TKR on average) (Figure 1C and 1D). For scores between 10 and 40, the costs remained approximately constant. For scores above 40, the predicted costs changed only slightly, while the uncertainty around these estimates increased considerably, since less than 0.4% of the patients in the sample have a score above 40. Costs for primary THR were lower (£22 on average) for women than for men (p<0.001), while the costs of TKR were higher (£9 on average) for women (p=0.026).

Figures 1E and 1F show the relationship between the cost of revision arthroplasty and pre-operative OHS/OKS score. Due to the smaller number of revisions, the uncertainty around these estimates is larger than for the costs of primary surgery, but the estimates suggest that the costs of THR revision surgery increased on average by £15 for a one-point increase in OHS (p=0.016), while the costs of TKR revision surgery remained constant (p=0.837 on the linear trend). The cost of revision arthroplasty was lower for older patients. Costs for THR revision surgery were on average £429 lower for women than for men (p<0.001).

Costs in the year before TJR and in the first 12 months after surgery

Costs in the year before THR decreased significantly with increasing OHS (e.g., £579 at OHS of 5 and £320 at OHS of 32; p=0.003), while costs before TKR varied little between OKS values of 0 and 20 (£897 at OKS of 10, £886 at OKS of 20), before decreasing thereafter (£513 at OKS of 32) (Figure 2A and 2B) However, for both THR and TKR the curves are relatively flat. Costs decreased (albeit insignificantly) with age for both THR and TKR. Since

For peer review only - http://bmjopen.bmj.com/site/about/guidelines.xhtml

there was only one patient with an OHS of 33, who was relatively young, the age effect produced an apparent spike in costs for an OHS score of 33.

Figures 2C and 2D show the estimated association between OHS/OKS and ambulatory costs in the first 12 months after TJR. Overall, ambulatory costs after THR were considerably lower than costs after TKR. A sensitivity analysis estimating ambulatory costs for TKR patients in COASt confirmed this finding, suggesting that this trend reflects differences between joints rather than between datasets. Ambulatory costs decreased with increasing OHS/OKS scores from £51/£446 at OHS/OKS scores of 5 and 6, respectively, to £46/£286 at OHS/OKS scores of 32. The negative predicted costs in Figure 2C are an artefact of the simple linear regression model, which does not restrict the outcome to be non-negative. It should be noted that the estimates for Figures 2A-D are based on substantially smaller numbers than previous figures and are subject to considerable uncertainty.

Costs of hospital readmissions

In line with the findings for the costs of primary surgery, the cost of hospital readmissions in the first 12 months after TJR decreased steeply with OHS/OKS for scores between 0 (£1,543 for THR, £1,700 for TKR) and 10 (£545 for THR, £684 for TKR), while for scores above 10 the curve was almost flat (Figure 2E and 2F). Both for THR and TKR the costs were lower for women than for men (on average £78 for THR and £133 for TKR; $p<0.001$ for both models).

Figure 3 shows the relationship between OHS/OKS and the annual costs of hospital readmissions between 1 and 6 years after primary arthroplasty. The costs of hospital readmissions per year for patients without revision surgery are shown in Figures 3A and 3B. For both THR and TKR the costs decreased with OHS/OKS (£161/£266 for OHS/OKS scores of 5 and 6, respectively, £85/£120 for OHS/OKS scores of 32; $p<0.001$). For THR, the costs in year 2 (i.e., between 12 and 24 months after THR) were significantly (on average £32; $p<0.001$) higher than in subsequent years. For TKR, the time trend indicates that readmission costs decreased over time (on average £31 per year; $p<0.001$).

Hospital readmission costs in the year of revision surgery did not have a statistically significant association with OHS/OKS scores measured before primary arthroplasty (Figures 3C and 3D).

Finally, the costs of hospital readmissions per year for patients who had undergone revision surgery >12 months previously (Figures 3E and 3F) were higher for patients who had low OHS/OKS scores before primary arthroplasty ($p<0.05$). However, the difference between patients with very low and very high scores was relatively small.

Table 2 summarizes the predicted quality-of-life and costs for patients with selected OHS/OKS scores averaged over the sample distribution of age and sex. Differences in quality-of-life between patients with very high scores (95th percentile) and very low scores (5th percentile) were very large (0.75/0.72 point difference in pre-operative EQ-5D utility and 0.28/0.35 point difference in post-operative utility for THR/TKR). Differences in costs were moderate in comparison. The costs of primary arthroplasty differed by £245 and £136 for THR and TKR, respectively. Costs in the year before surgery differed by £259 and £450, respectively. The cost of hospital readmissions in the first year differed by £427 and £484£ for THR and TKR, while annual costs for hospital readmissions in years 1 to 6 after primary arthroplasty differed by £76 and £146 for THR and TKR patients.

For peer review only - http://bmjopen.bmj.com/site/about/guidelines.xhtml

Table 3 shows predicted average quality-of-life and costs for patients in different age groups. Associations between quality-of-life and age were weak overall, the largest difference between patients aged 80 and above and patients below age 60 was predicted for utility after revision TKR revision surgery (0.1 point difference). Differences in costs were more pronounced, with differences in primary arthroplasty costs of £343 and £316 for THR and TKR. However, in contrast to Table 2 the differences were not systematic. For example, costs of primary arthroplasty, readmission costs in the first 12 months and readmission costs in the year of revision surgery were higher for older patients, while other costs (e.g., ambulatory costs in the first 12 months) were lower for older patients.

For peer review only

For peer review only - http://bmjopen.bmj.com/site/about/guidelines.xhtml

Table 2: Predicted quality-of-life and costs by pre-operative OHS/OKS

	THR			TKR		
**Outcome**	**OHS 5 (5th pct)**	**OHS 17 (50th pct)**	**OHS 32 (95th pct)**	**OKS 6 (5th pct)**	**OKS 18 (50th pct)**	**OKS 32 (95th pct)**
**A. Quality-of-life**						
Post-operative EQ-5D utility	0.612	0.798	0.890	0.498	0.735	0.851
Pre-operative EQ-5D utility	-0.082	0.354	0.671	-0.033	0.421	0.687
EQ-5D utility > 12 months after primary arthroplasty	-	-	-	0.501	0.700	0.813
EQ-5D utility before revision surgery	0.120	0.363	0.544	0.054	0.355	0.474
EQ-5D utility after revision surgery	0.437	0.673	0.783	0.293	0.572	0.684
**B. Costs (in £)**						
Costs of primary arthroplasty	5,687	5,502	5,442	6,158	6,036	6,021
Costs of revision arthroplasty	7,768	7,957	8,160	7,804	7,751	7,678
Costs in the 12 months before arthroplasty	579	416	320	963	898	513
Readmissions <12 months after primary arthroplasty	740	422	314	888	499	404
Ambulatory costs <12 months after primary arthroplasty	52	131	47	446	352	286
Readmissions >12 months after primary arthrolasty	161	115	85	266	169	120
Readmissions in the year of revision surgery	675	747	621	959	860	758
Readmissions > 12 months after revision surgery	238	144	73	360	225	150
Ambulatory costs >12 months after primary arthroplasty	-	-	-	48	27	19
Ambulatory costs in the year of revision surgery	-	-	-	816	533	351
Ambulatory costs for patients after revision surgery	-	-	-	128	155	36

Notes: The columns show average predicted quality-of-life and costs for patients with different pre-operative OHS and OKS scores. Predictions are averaged across the estimation sample. Column 1 and 4 show values for patients at the 5th percentile of the OHS/OKS distribution. Columns 2 and 4 show outcomes for patients at the median of OHS/OKS distributions, and columns 3 and 6 show outcomes for patients at the 95th percentile of the OHS/OKS distribution.

For peer review only

Table 3: Predicted quality-of-life and costs by age at operation

	THR			TKR		
**Outcome**	**Age <60**	**Age 60-79**	**Age ≥80**	**Age <60**	**Age 60-79**	**Age ≥80**
**A. Quality-of-life**						
Post-operative EQ-5D utility	0.778	0.794	0.729	0.637	0.731	0.722
Pre-operative EQ-5D utility	0.316	0.348	0.281	0.310	0.404	0.397
EQ-5D utility > 12 months after primary arthroplasty	-	-	-	0.616	0.689	0.645
EQ-5D utility before revision surgery	0.301	0.356	0.386	0.225	0.329	0.312
EQ-5D utility after revision surgery	0.572	0.670	0.562	0.418	0.558	0.521
**B. Costs (in £)**						
Costs of primary arthroplasty	5,420	5,504	5,763	5,939	6,039	6,255
Costs of revision arthroplasty	8,108	7,938	7,991	8,020	7,685	7,390
Costs in the 12 months before arthroplasty	626	394	398	1,150	804	634
Readmissions <12 months after primary arthroplasty	370	430	726	504	519	762
Ambulatory costs <12 months after primary arthroplasty	151	96	62	561	353	243
Readmissions >12 months after primary arthrolasty	135	117	110	218	171	156
Readmissions in the year of revision surgery	633	761	1,344	763	857	941
Readmissions > 12 months after revision surgery	159	151	139	428	242	105
Ambulatory costs >12 months after primary arthroplasty	-	-	-	50	27	27
Ambulatory costs in the year of revision surgery	-	-	-	711	556	160
Ambulatory costs for patients after revision surgery	-	-	-	145	124	118

Notes: The columns show average predicted quality-of-life and costs for patients in different age groups at primary operation. Predictions are averaged across the estimation sample.

DISCUSSION

OHS and OKS were developed as outcome measures reported by patients undergoing THR and TKR. Previous studies found that OHS and OKS are correlated with both clinical scores (such as the Charnley score or the American Knee Society score) and patient-reported general health measures (such as SF-36).[27,28] Thus, it is not surprising that we found that higher scores were systematically associated with higher quality-of-life. However, the difference between post-operative and pre-operative EQ-5D utility decreased with OHS/OKS. Patients with low scores have worse symptoms and more functional limitations due to osteoarthritis. Therefore, they have more potential to benefit from primary arthroplasty.

For peer review only - http://bmjopen.bmj.com/site/about/guidelines.xhtml

We also found that costs are moderately higher for patients with low pre-operative scores. Patients with worse symptoms due to osteoarthritis might require longer recovery times. In addition, they are likely to have comorbidities or complications, which also increase the costs of primary arthroplasty. While the differences in costs are moderate compared to differences in quality-of-life (between £100 and £500 for most outcomes), it is worth noting that some of these costs are incurred annually, and therefore even moderate differences can be economically important in the long-run.

Our findings do not allow us to draw conclusions about the cost-effectiveness of TJR for patients with different pre-operative scores without further assumptions about the costs and quality-of-life for patients without TJR. Nevertheless, based on these findings it appears promising to investigate differences in cost-effectiveness as well as the impact of rationing policies. In particular, the finding that the associations between pre-operative score and costs as well as quality-of-life are nonlinear suggests that there could be a threshold score beyond which TJR might not be considered beneficial or cost-effective. However, identifying this threshold is beyond the scope of this paper and left to future research.

Two previous studies on the cost-effectiveness of TKR and THR, respectively, found that while OHS/OKS were systematically associated with both costs and outcomes of TJR, both procedures were nonetheless cost-effective for almost all patients.[3,4] Similarly, our results indicate that quality-of-life improved for almost all patients undergoing TKR and THR. While the improvement was largest for patients with low scores, we also found that costs tended to be higher for patients with low scores.

A recent study concluded that, in the US, access to TKR could be restricted to patients with more severe symptoms.[14] Similar to our findings, they reported that changes in quality-of-life were lower for patients with fewer pre-operative symptoms (measured by SF-12 physical score). However, in their study TKR had minimal effects on quality-of-life on average (SF-6D utility increased by 0.008). Our findings contrast strongly with this, indicating that at least in the UK, TJR is associated with sizable increases in EQ-5D-3L utility (average change of 0.32 for TKR and 0.45 for THR) for almost all patients currently undergoing surgery. This discrepancy may be due to differences between EQ-5D-3L and SF-6D, differences between our sample covering almost all UK knee replacement procedures and a small US sample of early osteoarthritis patients, or methodological differences between the regression models selected for predictive accuracy in this study and marginal structural models used in the previous study.

Since OHS and OKS are already being used to determine eligibility for TJR,[6] evidence on their impact on the cost, efficacy and cost-effectiveness of primary arthroplasty is required to inform guidelines. The results presented in this study provide evidence on the association between pre-operative scores and important measures of costs and quality-of-life. Moreover, the models presented here can be used as inputs in future model-based studies assessing the cost, efficacy and/or cost-effectiveness of THR and TKR, and in particular to explore heterogeneity between patient subgroups.

In this study, we used data from a range of different sources, including clinical trials and comprehensive administrative databases. The sample sizes in this study are up to 1000 times larger than those used in previous studies.[3,4,14] Crucially, this reduces the uncertainty around our estimates for patients with very high scores – i.e., patients who are most likely to be affected by rationing. Our data also had a longer follow-up duration for TKR than previous studies (up to 12 years compared with five years in previous studies[4,14]).

For peer review only - http://bmjopen.bmj.com/site/about/guidelines.xhtml

Moreover, we employed a systematic model selection procedure to select the most appropriate statistical model from a range of different candidate models.

Nevertheless, there are several limitations to our study. KAT and COASt are markedly smaller than NHS PROMs-HES, and consequently models estimated on those datasets have more uncertainty and are relatively parsimonious. On the other hand, these datasets allowed us to estimate models for a larger range of relevant outcomes. While NHS PROMs-HES data include information on (nearly) all elective TJR procedures carried out in the NHS England, it nevertheless is a selected sample. In particular, pre-operative scores are already being used in some regions to restrict access to TJR, which affects the distribution of pre-operative scores in the data. Moreover, the findings of this study might not hold for patients that are currently denied access to TJR if those patients differ systematically from those observed in the data. Unfortunately, it is not possible to determine this without further information on the triage process. We were not able to draw conclusions on total costs or cost-effectiveness based on the findings of this study. This requires long-term modelling work, for which the models presented in this study could serve as parameters. The costs derived in this study could be affected by coding and classification errors. For example, we identified primary arthroplasty episodes in NHS PROMs-HES data based on links between NHS PROMs questionnaires and HES episodes instead of narrowly defined procedure and diagnosis codes. We were also not able to distinguish between one-stage and two-stage revisions. Consequently, our estimates of the costs could be affected by miscoded diagnoses, procedures or data linkage. We used specific criteria to determine whether hospital readmissions were related to primary arthroplasty. Similarly, in COASt and KAT patients were asked to report health services used for problems related to their joint and their primary arthroplasty operation. Therefore, ambulatory and hospital readmission costs might be affected by classification errors. While coding and classification errors might bias the costs reported in this study, they are unlikely to affect the estimated associations between OHS/OKS and costs, since it does not seem plausible that the frequency of such errors is related to patient’s pre-operative score. The costs were derived from the National Tariff as well as published reference costs. In some cases these costs might not reflect the true costs to the hospital, although there is no reason to suspect that they would differ systematically.

We conducted a complete-case analysis, since the use of multiple imputation would have further increased the complexity of our analysis. However, there is little reason to expect that missing data would be systematically related to the shape of the relationship between OHS/OKS and costs or quality-of-life. While we studied a wide range of outcome variables, we were not able to examine revision rates or long-term outcomes for THR due to a lack of appropriate data. We also focused on a limited range of covariates (OHS/OKS, age and sex). We did not investigate the impact of other covariates that may affect our outcomes and are used in clinical decision-making (e.g., body mass index) due to a lack of data. While we tested a wide range of candidate models, our model selection was done sequentially, and therefore it is possible that there are interactions between model components that were not considered in this study. Our model selection process was developed to assess prediction accuracy, and therefore we did not consider statistical significance or confounding when selecting our models. Finally, our model selection process did not take into account whether differences in model fit were statistically significant and it did not penalize model complexity.

In summary, our results show that pre-operative OHS and OKS are systematically associated with the costs and quality-of-life of patients undergoing TJR. However, further research is

For peer review only - http://bmjopen.bmj.com/site/about/guidelines.xhtml

needed to estimate cost-effectiveness, and to determine the impact on revision rates and long-term outcomes for THR.

Acknowledgements

We would like to thank the ACHE investigators, user group and steering group for their role in the project.

We thank the KAT study group for providing access to their data for use in this project, as well as all of the researchers and participants involved in that study. We would like to thank all the participants of the COASt Study, Professor Nigel Arden and the COASt team for their time and dedication, and the NIHR for their funding support.

KAT was funded by the NIHR as an HTA (No. 95/10/01). COASt was funded by the National Institute for Health Research (NIHR) under its Programme Grants for Applied Research Programme (Reference Number RP-PG-0407-10064).

Data on NHS PROMs linked to HES APC data were reused with the permission of NHS Digital, copyright 2015, with all rights reserved.

The views and opinions expressed in this paper are those of the authors and do not necessarily reflect those of the HTA programme, NIHR, NHS, NHS Digital or the Department of Health.

We would also like to thank Chaudhry Shah and Joaquim Soares do Brito for their help with identifying the relevant codes for hospital readmissions used in the HES data.

Funding

This study was conducted as part of the project "Introducing Standardised And Evidence-Based Thresholds for Hip and Knee Replacement Surgery – The Arthroplasty Candidacy Help Engine (The ACHE Tool)", which was funded under the UK National Institute of Health Research (NIHR) Health Technologies Assessment (HTA) Programme (HTA 11/63/01). Visit the HTA programme website (www.hta.ac.uk) for further project information. The funder had no involvement in: the collection, analysis, and interpretation of data; in the writing of the report; and in the decision to submit the article for publication. The authors had full access to the data (including statistical reports and tables), can take responsibility for the integrity of the data and the accuracy of the data analysis and are responsible for submitting this paper.

Competing interest statement

All authors have completed the Unified Competing Interest form at www.icmje.org/coi_disclosure.pdf (available on request from the corresponding author). All authors received a grant from NIHR funding the current work. HD reports personal fees from Halyard Health, outside the submitted work. NA reports grants from Bioiberica, personal fees from Bioventus, personal fees from ESCEO, personal fees from Flexion, personal fees from Freshfields Bruckhaus Deringer, personal fees from Merck, personal fees from Regeneron, outside the submitted work. The other authors declare that there are no relevant conflicts of interests.

For peer review only - http://bmjopen.bmj.com/site/about/guidelines.xhtml

Ethical Approval
No primary data was collected for this study, and therefore ethical approval was not required. The COASt study obtained ethical approval from Oxford research Ethics Committee (REC Ref: 10/H0604/91) and written consent was obtained from each participant. The KAT trial was approved by the Multi Centre Research Ethics Committee for Scotland in November 1998 (research protocol MREC/98/0/100) and was approved by the Local Research Ethics Committees in each study centre recruiting trial participants.

Copyright/licence
The Corresponding Author has the right to grant on behalf of all authors and does grant on behalf of all authors, a worldwide licence to the Publishers and its licensees in perpetuity, in all forms, formats and media (whether known now or created in the future), to i) publish, reproduce, distribute, display and store the Contribution, ii) translate the Contribution into other languages, create adaptations, reprints, include within collections and create summaries, extracts and/or, abstracts of the Contribution, iii) create any other derivative work(s) based on the Contribution, iv) to exploit all subsidiary rights in the Contribution, v) the inclusion of electronic links from the Contribution to third party material where-ever it may be located; and, vi) licence any third party to do any or all of the above.

Authorship statement

AP, DB, HD, AG and NA conceived, designed and conducted the ACHE study. PE, HD and AG designed and conducted the regression analysis reported in this manuscript. PE and HD analysed the data. PE drafted the manuscript. All authors edited the manuscript for important intellectual content and approved the final version.

Transparency declaration
The manuscript's guarantor (PE) affirms that the manuscript is an honest, accurate, and transparent account of the study being reported; that no important aspects of the study have been omitted; and that any discrepancies from the study as planned (and, if relevant, registered) have been explained.

Data sharing
The statistical code as well as variance-covariance matrices for the analyses reported in this study are available from the authors upon request. PROMs/HES data are available from NHS Digital (http://content.digital.nhs.uk/dars); enquiries about access to the KAT data should be directed to kat@abdn.ac.uk; enquiries about access to the COASt data should be directed to coast.study@ndorms.ox.ac.uk.

For peer review only - http://bmjopen.bmj.com/site/about/guidelines.xhtml

References

1 Gandhi R, Dhotar H, Davey JR, *et al.* Predicting the Longer-term Outcomes of Total Hip Replacement. *J Rheumatol* 2010;**37**:2573–7. doi:10.3899/jrheum.100149

2 Judge A, Batra RN, Thomas GE, *et al.* Body mass index is not a clinically meaningful predictor of patient reported outcomes of primary hip replacement surgery: prospective cohort study. *Osteoarthritis Cartilage* 2014;**22**:431–9. doi:10.1016/j.joca.2013.12.018

3 Fordham R, Skinner J, Wang X, *et al.* The economic benefit of hip replacement: a 5-year follow-up of costs and outcomes in the Exeter Primary Outcomes Study. *BMJ Open* 2012;**2**:e000752. doi:10.1136/bmjopen-2011-000752

4 Dakin H, Gray A, Fitzpatrick R, *et al.* Rationing of total knee replacement: a cost-effectiveness analysis on a large trial data set. *BMJ Open* 2012;**2**:e000332. doi:10.1136/bmjopen-2011-000332

5 Gandhi R, Dhotar H, Razak F, *et al.* Predicting the longer term outcomes of total knee arthroplasty. *The Knee* 2010;**17**:15–8. doi:10.1016/j.knee.2009.06.003

6 The Royal College of Surgeons of England. Is access to surgery a postcode lottery? Published Online First: 2014.https://www.rcseng.ac.uk/-/media/files/rcs/news-and-events/media-centre/is-access-to-surgery-a-postcode-lottery.pdf?la=en (accessed 8 Nov 2016).

7 Coastal West Sussex Clinical Commissioning Group. Low Priority Procedures (LPPs) and Procedures with Restrictions (OPRs), Commissioning for Clinical Effectiveness, NICE Interventional Procedures, version 2.10. Published Online First: 2015.https://www.coastalwestsussexccg.nhs.uk/domains/coastal-west-sussex-ccg.org.uk/local/media/documents/misc/Version_2.10_Low_Priority_Procedures_and_Other_Procedures_with_Restrictions_2015.pdf (accessed 8 Nov 2016).

8 Harrogate and Rural District Clinical Commissioning Group. Clinical thresholds: Hip and knee arthroplasty for osteoarthritis (only). Published Online First: 2014.http://www.harrogateandruraldistrictccg.nhs.uk/data/uploads/rss2/hip-and-knee-arthroplasty.pdf (accessed 8 Nov 2016).

9 Scarborough and Ryedale Clinical Commissioning Group. Hip replacement pathway. Published Online First: 2015.http://www.scarboroughryedaleccg.nhs.uk/data/uploads/rss2/orthopaedics/hip-replacement-march-2015.pdf (accessed 8 Nov 2016).

10 Worcestershire NHS. Commissioning Policy for Musculoskeletal Surgical Interventions. Published Online First: 2010.www.worcestershire.nhs.uk/EasySiteWeb/GatewayLink.aspx?alId=5553 (accessed 8 Nov 2016).

11 Losina E, Walensky RP, Kessler CL, *et al.* Cost-effectiveness of Total Knee Arthroplasty in the United States. *Arch Intern Med* 2009;**169**:1113–22. doi:10.1001/archinternmed.2009.136

For peer review only - http://bmjopen.bmj.com/site/about/guidelines.xhtml

12 Rissanen P, Aro S, Sintonen H, *et al.* Costs and cost-effectiveness in hip and knee replacements. A prospective study. *Int J Technol Assess Health Care* 1997;**13**:575–88.

13 Cushnaghan J, Bennett J, Reading I, *et al.* Long-term outcome following total knee arthroplasty: a controlled longitudinal study. *Ann Rheum Dis* 2009;**68**:642–7. doi:10.1136/ard.2008.093229

14 Ferket BS, Feldman Z, Zhou J, *et al.* Impact of total knee replacement practice: cost effectiveness analysis of data from the Osteoarthritis Initiative. *BMJ* 2017;:j1131. doi:10.1136/bmj.j1131

15 Williams DP, Price AJ, Beard DJ, *et al.* The effects of age on patient-reported outcome measures in total knee replacements. *Bone Jt J* 2013;**95–B**:38–44. doi:10.1302/0301-620X.95B1.28061

16 Judge A, Arden NK, Batra RN, *et al.* The association of patient characteristics and surgical variables on symptoms of pain and function over 5 years following primary hip-replacement surgery: a prospective cohort study. *BMJ Open* 2013;**3**:e002453. doi:10.1136/bmjopen-2012-002453

17 Murray DW, MacLennan GS, Breeman S, *et al.* A randomised controlled trial of the clinical effectiveness and cost-effectiveness of different knee prostheses: the Knee Arthroplasty Trial (KAT). *Health Technol Assess Winch Engl* 2014;**18**:1–235, vii–viii. doi:10.3310/hta18190

18 Arden N, Altman D, Beard D, *et al.* Lower limb arthroplasty: can we predict outcome and failure and is it cost effective? *Health Technol Assess* 2016;**(in press)**.

19 Herbert A, Wijlaars L, Zylbersztejn A, *et al.* Data Resource Profile: Hospital Episode Statistics Admitted Patient Care (HES APC). *Int J Epidemiol* doi:10.1093/ije/dyx015

20 Judge A, Cooper C, Williams S, *et al.* Patient-reported outcomes one year after primary hip replacement in a European Collaborative Cohort. *Arthritis Care Res* 2010;**62**:480–8. doi:10.1002/acr.20038

21 Marques EM, Blom AW, Lenguerrand E, *et al.* Local anaesthetic wound infiltration in addition to standard anaesthetic regimen in total hip and knee replacement: long-term cost-effectiveness analyses alongside the APEX randomised controlled trials. *BMC Med* 2015;**13**:151. doi:10.1186/s12916-015-0389-1

22 Wylde V, Blom AW, Bolink S, *et al.* Assessing function in patients undergoing joint replacement: a study protocol for a cohort study. *BMC Musculoskelet Disord* 2012;**13**:220. doi:10.1186/1471-2474-13-220

23 Feng Y, Parkin D, Devlin NJ. Assessing the performance of the EQ-VAS in the NHS PROMs programme. *Qual Life Res* 2014;**23**:977–89. doi:10.1007/s11136-013-0537-z

24 Dolan P. Modeling Valuations for EuroQol Health States. *Med Care* 1997;**35**:1095–108.

25 The National Casemix Office. HRG4 Grouper Reference Manual Payment 14/15. content.digital.nhs.uk/article/3938/HRG4-201415-Payment-Grouper?tabid=1 (accessed 10 Jun 2015).

For peer review only - http://bmjopen.bmj.com/site/about/guidelines.xhtml

26 Department of Health. Payment by Results in the NHS: tariff for 2014 to 2015: 2014- 15 tariff information spreadsheet. Published Online First: 2014.https://www.gov.uk/government/uploads/system/uploads/attachment_data/file/300551/Annex _5A_National_Prices.xlsx (accessed 19 Oct 2015).

27 Dawson J, Fitzpatrick R, Carr A, *et al.* Questionnaire on the Perceptions of Patients About Total Hip Replacement. *J Bone Jt Surg Br* 1996;**78**–**B**:185–90.

28 Dawson J, Fitzpatrick R, Murray D, *et al.* Questionnaire on the perceptions of patients about total knee replacement. *J Bone Jt Surg Br* 1998;**80**–**B**:63–9.

For peer review only

Figure legends

Figure 1

Sources: NHS PROMs and HES data, 2009-2015; own calculations. Costs are measured in 2014 Pound Sterling (£). Lines show predicted values averaged over all observations in the sample with a given OHS/OKS. All models included pre-operative OHS or OKS score, age at operation and sex of the patient as independent variables, with the exception of the model for pre-operative EQ-5D of THR patients, which did not include an indicator for female patients. Full regression results are shown in appendix C. The grey areas show 95% confidence intervals obtained from 1,000 bootstrap replications.

Figure 2

Sources: NHS PROMs, HES, KAT and COASt data, 2009-2015; own calculations. Costs are measured in 2014 Pound Sterling (£). Lines show predicted values averaged over all observations in the sample with a given OHS/OKS. All models included pre-operative OHS or OKS score and age at operation as independent variables. The models for readmission costs also included a binary indicator for female patients. Full regression results are shown in appendix C. The grey areas show 95% confidence intervals obtained from 1,000 bootstrap replications.

Figure 3

Sources: NHS PROMs and HES data, 2009-2015; own calculations. Costs are measured in 2014 Pound Sterling (£). Lines show predicted values averaged over all observations in the sample with a given OHS/OKS. All models included pre-operative OHS or OKS score as independent variables. The model for annual readmission costs between 1 and 6 years after THR additionally included current age and an indicator for time since primary surgery. The corresponding model for TKR included age at operation, time since primary surgery and an indicator for sex of the patient as independent variables. The models in panel B additionally included age at operation, time since primary surgery and sex of the patient as covariates. The model for annual readmission costs after revision TKR surgery included current age and time since primary surgery as additional covariates. Full regression results are shown in appendix C. The grey areas show 95% confidence intervals obtained from 1,000 bootstrap replications.

For peer review only - http://bmjopen.bmj.com/site/about/guidelines.xhtml

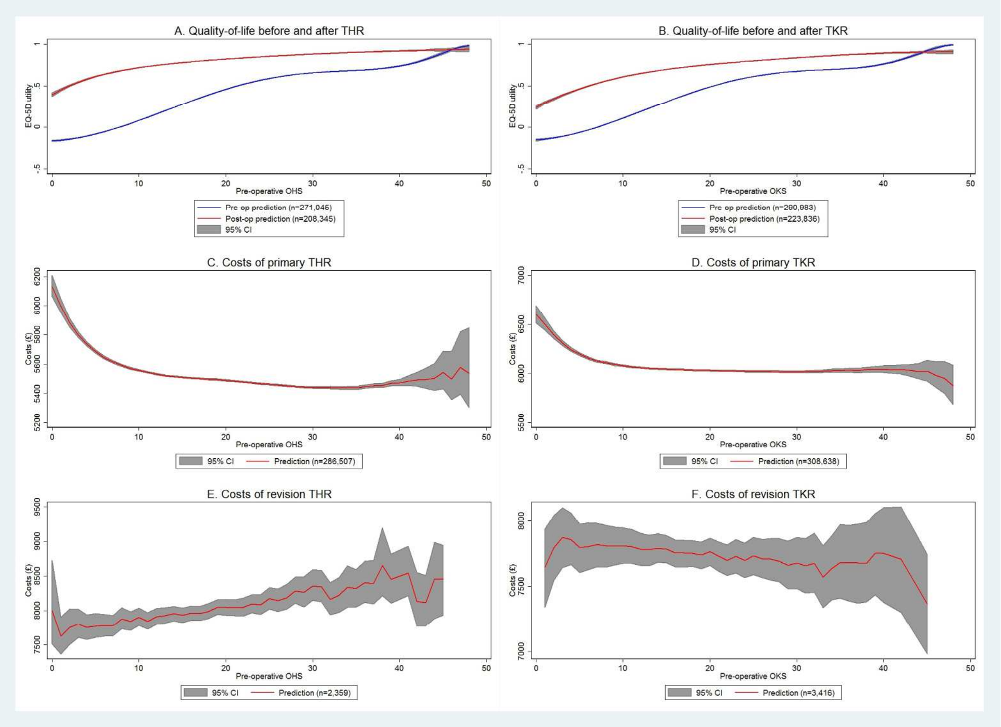

203x147mm (300 x 300 DPI)

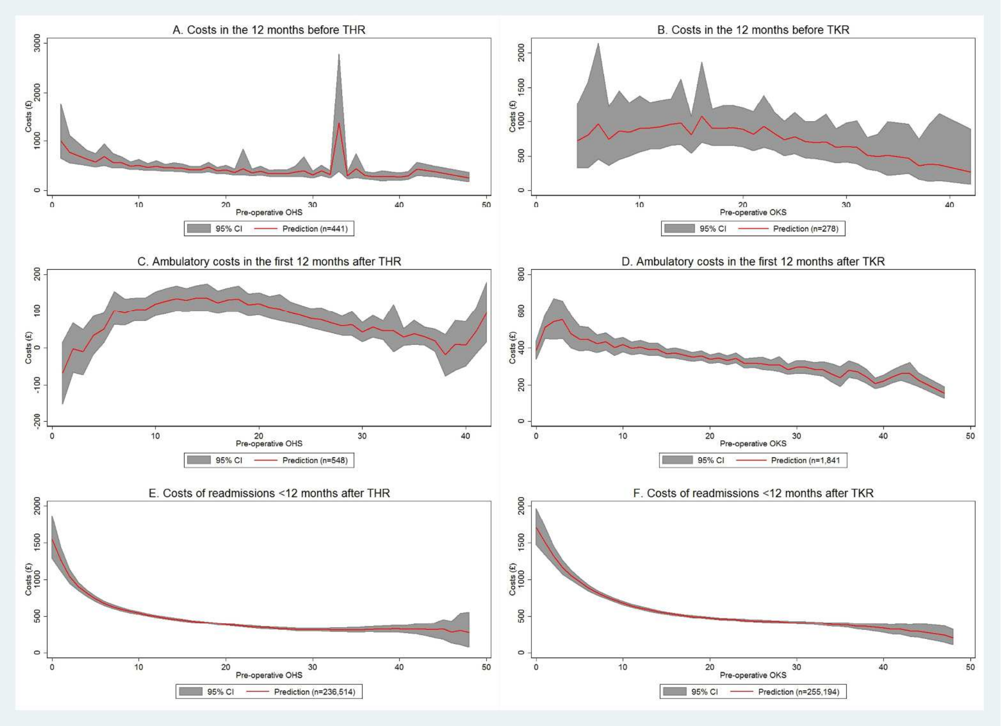

203x147mm (300 x 300 DPI)

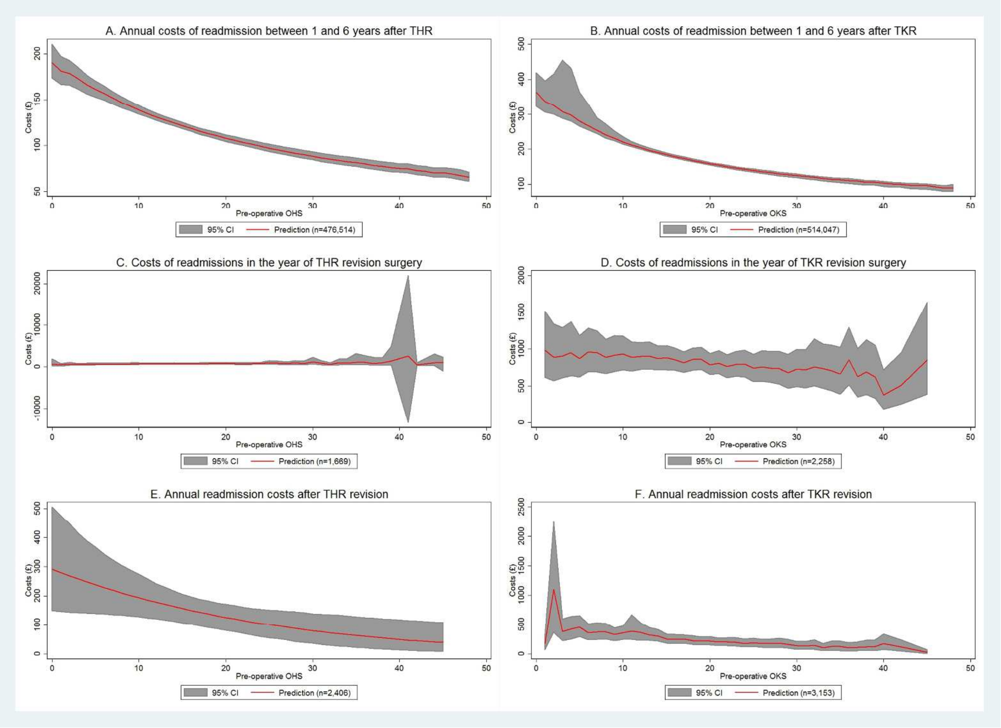

203x147mm (300 x 300 DPI)

ew only

Appendix

A. Diagnoses and procedures codes used to define hospital readmissions

We used ICD-10 diagnosis codes and OPCS 4 procedure codes to determine whether a hospital admission was related to arthroplasty. Specifically, we used the following criteria:

- A hospital admission occurred within 30 days after the primary arthroplasty episode. This criterion was applied regardless of any specific diagnosis or procedure codes.
- The primary diagnosis (i.e., the first recorded ICD-10 diagnosis code) was M17.X (knee arthritis) for a knee replacement episode, or M16.X (hip arthritis) for a hip replacement episode.
- One of the recorded procedure codes referred to the hip or knee joint. The OPCS 4 codes Z843, Z761 or Z756 were used for hip replacement episodes, and the codes Z846, Z765, Z845. Z844, Z774 or Z787 were used for knee replacement episodes.
- The admission had a diagnosis code associated with infections of the skin, the joint or the prosthesis. We used the following ICD-10 codes for this purpose:
    - M25.X with Z96.6 – "Other joint disorder, not elsewhere classified" and "Presence of orthopedic joint implant"
    - T81.X with either Z96.6 or Y83.1 – "Complications of procedures, not elsewhere classified" and "Presence of orthopedic joint implant" or "Surgical operation with implant of artificial internal device […]"
    - T84.X – "Complications of internal orthopedic prosthetic devices, implants and grafts"
    - M96.X – "Intraoperative and postprocedural complications and disorders of musculoskeletal system, not elsewhere classified"
    - M00.X with Z96.6 – "Pyogenic arthritis" and "Presence of orthopedic joint implant"
    - M86.X with Z96.6 – "Osteomyelitis" and "Presence of orthopedic joint implant".

For peer review only

B. Unit costs

Table B.1 Unit costs applied to all primary datasets providing resource use data

Healthcare resource	Units	Unit cost	Reference and details
GP surgery visit	Per visit	£46	PSSRU 2014 page 195[1]: Cost of a surgery patient contact lasting 11.7 minutes was £46 including direct care staff costs and qualifications. A clinic patient contact lasting 17.2 minutes costs £67 including direct care staff costs and qualifications.
GP Practice nurse visit	Per visit	£13.69	PSSRU 2014 page 192: £53 per hour of face-to-face contact including qualifications (£44/hour excluding qualifications). Average surgery consultation for a GP practice nurse lasts 15.5 minutes, giving a consultation cost of £14
Community physiotherapist at clinic/GP surgery	Per visit	£51	NHS reference costs – PSSRU 2014 page 179 states that the mean cost for a one-to-one contact in physiotherapy services in 2013/2014 was £51. PSSRU estimates the unit cost per hour in 2013/2014 to be £32 without qualifications or £36 with qualifications (page 179)
Community physiotherapist – at home	Per visit	£51	For simplicity, applied the same cost as physiotherapy at GP surgery/clinic
Hospital physiotherapist	Per visit	£34	NHS reference costs – PSSRU 2014 page 235 states that the mean average cost for a non-consultant led (not admitted) follow-up physiotherapy attendance in 2013/14 was £34 (IQR £28-£38). PSSRU 2014 estimates the hourly cost of a hospital physiotherapist to be £37/hour including qualifications and £33 excluding qualifications
Physiotherapy – average	Per visit	£42.50	We assumed that 50% of physiotherapy visits are in hospital and 0% are in the community.

For peer review only

Healthcare resource	Units	Unit cost	Reference and details
Outpatient visit – orthopaedics – Follow-up visit	Per visit	£71	Payment by results tariff [2] Tariff information spreadsheet 2013-14: outpatient attendances- WF01A Follow Up Attendance - Single Professional Or WF02A Follow Up Attendance - Multi-Professional (both of these codes are the same price). Follow-up attendance is used here, since patients are assumed to have already attended clinic for their joint replacement
A&E attendance – not admitted	Per visit	£81.26	National schedule of reference costs [3] weighted average of all non-admitted accident and emergency attendances , weighted by the number of attendances.
Admission to hospital related to hip arthroplasty (No data on length of stay)	Per admission	£2,662	Department of Health reference costs 2013-14. [3] This was calculated by taking a weighted average cost of all HRGs with "hip" within the currency description on the EL & NEL worksheets, weighting by the number of FCEs.
Admission to hospital related to knee arthroplasty (No data on length of stay)	Per admission	£3,907	Department of Health reference costs 2013-14 [3]. This was calculated by taking a weighted average cost of all HRGs with "knee" within the currency description on the EL & NEL worksheets, weighting by the number of FCEs.
Readmission to hospital within 30 days of hip or knee arthroplasty	Per bed-day	£395.83	Average costs per bed-day for readmissions in PROMs/HES within 30 days of hip or knee arthroplasty
Readmission to hospital within 30 days of hip or knee arthroplasty (No data on length of stay)	Per admission	£1,255	Average costs for readmissions in PROMs/HES within 30 days of hip or knee arthroplasty
Readmission to hospital with a diagnosis of hip/knee arthritis after hip/knee arthroplasty	Per bed-day	£1,107.25	Average costs per bed-day for readmissions in PROMs/HES with a diagnosis of hip/knee arthritis after hip/knee arthroplasty
Readmission to hospital with a diagnosis of	Per	£5,220	Average costs for readmissions in PROMs/HES with a diagnosis of

For peer review only

Healthcare resource	Units	Unit cost	Reference and details
hip/knee arthritis after hip/knee arthroplasty (No data on length of stay)	admission		hip/knee arthritis after hip/knee arthroplasty
Readmission to hospital for a hip/knee-specific procedure following hip/knee arthroplasty	Per bed-day	£551.33	Average costs per bed-day for readmissions in PROMs/HES for a hip/knee-specific procedure following hip/knee arthroplasty
Readmission to hospital for a hip/knee-specific procedure following hip/knee arthroplasty (No data on length of stay)	Per admission	£1,654	Average costs for readmissions in PROMs/HES for a hip/knee-specific procedure following hip/knee arthroplasty
Readmission to hospital with a diagnosis for infection following hip/knee arthroplasty	Per bed-day	£694.8	Average costs per bed-day for readmissions in PROMs/HES with a diagnosis for infection following hip/knee arthroplasty
Readmission to hospital with a diagnosis for infection following hip/knee arthroplasty (No data on length of stay)	Per admission	£1,876	Average costs for readmissions in PROMs/HES diagnosis for infection following hip/knee arthroplasty

References

[1] PSSRU | Unit Costs of Health and Social Care 2014 n.d. http://www.pssru.ac.uk/project-pages/unit-costs/2014/ (accessed June 14, 2017).
[2] Department of Health. Payment by Results in the NHS: tariff for 2014 to 2015: 2014- 15 tariff information spreadsheet. 2014.
[3] Department of Health. NHS reference costs 2013 to 2014 2014. https://www.gov.uk/government/publications/nhs-reference-costs-2013-to-2014 (accessed June 14, 2017).

For peer review only

C. Regression results

Table C.1: Associations between pre-operative OHS/OKS and post-operative EQ-5D-3L

	Hip arthroplasty			Knee arthroplasty		
Model	*Tobit model censored at -0.594 and 1*			*Tobit model censored at -0.594 and 1*		
**Variable**	**Coefficient**		**Robust S.E.**	**Coefficient**		**Robust S.E.**
OHS/OKS						
Linear term	5.839E-02	***	4.337E-03	5.725E-02	***	2.482E-03
Quadratic term	-3.312E-03	***	5.400E-04	-2.356E-03	***	2.008E-04
Cubic term	1.182E-04	***	2.990E-05	5.390E-05	***	6.600E-06
Quartic term	-2.110E-06	**	7.520E-07	-4.660E-07	***	7.530E-08
Quintic term	1.460E-08	*	6.980E-09	-	-	-
Age (reference category: 50-54)						
Age <30	-9.039E-02	***	1.717E-02	-1.467E-01	*	6.181E-02
Age 30-39	-4.423E-02	***	1.047E-02	-4.303E-02		2.371E-02
Age 40-49	-1.240E-02	*	6.128E-03	-2.316E-02	**	6.787E-03
Age 55-59	3.204E-03		5.012E-03	1.138E-02	*	4.470E-03
Age 60-64	2.120E-02	***	4.592E-03	4.654E-02	***	4.094E-03
Age 65-69	3.235E-02	***	4.422E-03	8.938E-02	***	3.980E-03
Age 70-74	3.831E-03		4.377E-03	8.991E-02	***	3.966E-03
Age 75-79	-2.073E-02	***	4.400E-03	8.561E-02	***	4.000E-03
Age 80-84	-4.607E-02	***	4.629E-03	8.467E-02	***	4.208E-03
Age 85-89	-6.786E-02	***	5.558E-03	7.706E-02	***	5.099E-03
Age 90-94	-6.984E-02	***	1.027E-02	7.987E-02	***	1.066E-02
Age >=95	-1.072E-01	**	4.130E-02	9.356E-02		4.858E-02
Female sex	-3.099E-02	***	1.691E-03	-	-	-
Constant term	4.451E-01	***	1.297E-02	1.741E-01	***	1.105E-02
σ (Tobit term)	3.442E-01	-	8.904E-04	2.997E-01	-	7.109E-04
N	208,345			223,836		
Data	PROMs-HES			PROMs-HES		
Candidate models						
Model class	OLS, Tobit, two-part (Logit-OLS)			OLS, Tobit, two-part (Logit-OLS)		
OHS/OKS trend	Quadratic, cubic, quartic, quintic, log-linear			Quadratic, cubic, quartic, quintic, log-linear		
Age trend	Linear, quadratic, cubic, quartic, quintic, five-year bands, exclusion			Linear, quadratic, cubic, quartic, quintic, linear spline (65), five-year bands, exclusion		
Sex	yes/no			yes/no		

For peer review only

Table C.2: Associations between pre-operative OHS/OKS and EQ-5D-3L before TJR

	Hip arthroplasty			Knee arthroplasty		
Model	*Tobit model censored at -0.594 and 1*			*Tobit model censored at -0.594 and 1*		
**Variable**	**Coefficient**		**Robust S.E.**	**Coefficient**		**Robust S.E.**
			OHS/OKS			
Linear term	5.719E-03	**	2.040E-03	1.001E-02	***	2.403E-03
Quadratic term	2.385E-03	***	2.641E-04	2.150E-03	***	2.997E-04
Cubic term	-3.800E-05	*	1.500E-05	-3.140E-05		1.660E-05
Quartic term	-1.480E-06	***	3.850E-07	-1.650E-06	***	4.190E-07
Quintic term	3.010E-08	***	3.650E-09	3.240E-08	***	3.920E-09
			Age			
Linear term	-8.742E-03	***	3.866E-03	-2.943E-02	**	1.046E-02
Quadratic term	3.742E-04		1.062E-04	7.750E-04	**	2.541E-04
Cubic term	-5.190E-06	***	1.240E-06	-8.360E-06	**	2.700E-06
Quartic term	2.340E-08		5.290E-09	3.270E-08	**	1.060E-08
Female sex	-	-	-	1.040E-02	***	8.351E-04
Constant term	-1.649E-01	**	5.079E-02	1.791E-01		1.592E-01
σ (Tobit term)	2.128E-01	-	3.022E-04	2.177E-01	-	2.895E-04
N	271,045			290,983		
Data	PROMs-HES			PROMs-HES		
			Candidate models			
Model class	OLS, Tobit, two-part (Logit-OLS)			OLS, Tobit, two-part (Logit-OLS)		
OHS/OKS trend	Quadratic, cubic, quartic, quintic, log-linear			Quadratic, cubic, quartic, quintic, log-linear		
Age trend	Linear, quadratic, cubic, quartic, quintic, five-year bands, exclusion			Linear, quadratic, cubic, quartic, quintic, five-year bands, exclusion		
Sex	yes/no			yes/no		

Sources: NHS PROMs and HES data, 2009-2015; own calculations.* p<0.05; ** p<0.01; *** p<0.001. Details on how to calculate predictions are provided in Appendix D.

For peer review only

Table C.3: Associations between pre-operative OKS and EQ-5D-3L between 1 and 12 years after TKR

	Knee arthroplasty		
Model	*OLS*		
**Variable**	**Coefficient**		**Clustered S.E.**
	OKS		
Log-linear term	1.776E-01	***	1.111E-02
	Current age		
Linear term	7.168E-03	***	1.541E-03
Linear spline term (70)	-8.851E-03	**	2.551E-03
Year since operation	-6.154E-03	***	1.417E-03
Interaction between year and linear spline term	-3.729E-04	*	1.669E-04
Previously revised	-1.598E-01	***	3.239E-02
Constant term	-2.372E-01	*	1.031E-01
N	15,414 (2,004 patients)		
Data	KAT		
	Candidate models		
Model class	OLS with clustered standard errors, linear mixed models with random intercept and fixed slopes		
Time trend	Linear, quadratic		
OKS trend	Linear, quadratic, cubic, log-linear		
Age trend	For both age at operation and current age: Linear, quadratic, cubic, linear spline (70), exclusion		
Sex	yes/no		

Sources: KAT data; own calculations.* p<0.05; ** p<0.01; *** p<0.001. Details on how to calculate predictions are provided in Appendix D. The model was estimated on data in long-format (i.e., one observation per patient and year). The inclusion of the time trend was tested after selecting the model class and before choosing a trend for OKS. The linear spline term equals "(current age - 70)" for patients above 70, and "0" for those below. The interaction term is derived by multiplying the linear spline term with the year variable. We did not estimate a model for hips due to a lack of data.

For peer review only

Table C.4: Associations between pre-operative OHS/OKS and EQ-5D-3L before revision surgery

	Hip arthroplasty			Knee arthroplasty		
Model	*Tobit model censored at -0.594 and 1*			*Tobit model censored at -0.594 and 1*		
**Variable**	**Coefficient**		**Clustered S.E.**	**Coefficient**		**Clustered S.E.**
OHS/OKS						
Linear term	2.665E-02	***	4.188E-03	2.631E-02	**	8.271E-03
Quadratic term	-2.867E-04	**	1.041E-04	2.356E-04		4.623E-04
Cubic term	-	-	-	-1.510E-05		7.740E-06
Age at primary operation						
Linear term	-	-	-	4.472E-03	***	9.990E-04
Linear spline term (70)	-	-	-	-5.607E-03		2.879E-03
Age at revision surgery						
Linear term	2.123E-03	**	7.587E-04	-	-	-
Logarithm of year of revision	-	-	-	-3.601E-02		1.882E-02
Male sex	-	-	-	-2.358E-02		1.368E-02
Constant term	-1.444E-01	*	6.087E-02	-3.498E-01	***	7.534E-02
σ (Tobit term)	3.178E-01	-	4.755E-03	2.999E-01	-	3.676E-03
N	1,391 (1,331 patients)			2,227 (2,073 patients)		
Data	PROMs-HES			PROMs-HES		
Candidate models						
Model class	OLS, Tobit			OLS, Tobit		
Year of revision (time trend)	Linear, exclusion			Linear, quadratic, cubic, log-linear, exclusion		
OHS/OKS trend	Linear, quadratic, cubic, log-linear			Linear, quadratic, cubic, quartic, log-linear, linear spline (30)		
Age trend	Age at operation vs. age at revision surgery; for age at revision surgery: linear, quadratic, cubic, exclusion			Age at operation vs. age at revision surgery; for age at operation: linear, quadratic, cubic, linear spline (70), exclusion		
Sex	yes/no			yes/no		

Sources: NHS PROMs and HES data, 2009-2015; own calculations.* p<0.05; ** p<0.01; *** p<0.001. Details on how to calculate predictions are provided in Appendix D. The linear spline term equals "(age at operation - 70)" for patients above 70, and "0" for those below.

For peer review only

Table C.5: Associations between pre-operative OHS/OKS and EQ-5D-3L after revision surgery

Model	Hip arthroplasty *Tobit model censored at -0.594 and 1*			Knee arthroplasty *Tobit model censored at -0.594 and 1*		
**Variable**	**Coefficient**		**Clustered S.E.**	**Coefficient**		**Clustered S.E.**
	OHS/OKS					
Log-linear term	2.201E-01	***	2.580E-02	2.556E-01	***	1.950E-02
	Age at revision surgery					
Linear term	-8.181E-02		6.954E-02	1.352E-02	***	2.570E-03
Quadratic term	1.755E-03		1.099E-03	-	-	-
Cubic term	-1.120E-05		5.680E-06	-	-	-
Linear spline term (65)	-	-	-	-1.533E-02	***	3.747E-03
Male sex	-5.907E-02	*	2.696E-02	-8.874E-02	***	1.982E-02
Constant term	1.120E+00		1.445E+00	-9.426E-01	***	1.623E-01
σ (Tobit term)	3.633E-01	-	1.184E-02	3.441E-01	-	7.576E-03
N	884 (860 patients)			1,398 (1,331 patients)		
Data	PROMs-HES			PROMs-HES		
	Candidate models					
Model class	OLS, Tobit, two-part (Logit-OLS)			OLS, Tobit, two-part (Logit-OLS)		
Year of revision (time trend)	Linear, exclusion			Linear, quadratic, cubic, log-linear, exclusion		
OHS/OKS trend	Linear, quadratic, cubic, log-linear			Linear, quadratic, cubic, quartic, log-linear		
Age trend	Age at operation vs. age at revision surgery; for age at revision surgery: linear, quadratic, cubic, exclusion			Age at operation vs. age at revision surgery; for age at revision surgery: linear, quadratic, cubic, linear spline (65), exclusion		
Sex	yes/no			yes/no		

Sources: NHS PROMs and HES data, 2009-2015; own calculations.* p<0.05; ** p<0.01; *** p<0.001. Details on how to calculate predictions are provided in Appendix D. The linear spline term equals "(age at revision surgery - 65)" for patients above 65, and "0" for those below.

For peer review only

Table C.6: Associations between pre-operative OHS/OKS and the costs of primary arthroplasty

	Hip arthroplasty			Knee arthroplasty		
Model	*gamma-GLM with inverse link function*			*gamma-GLM with inverse link function*		
**Variable**	**Coefficient**		**S.E.**	**Coefficient**		**SE**
			OHS/OKS			
Linear term	3.100E-06	***	1.970E-07	2.670E-06	***	2.920E-07
Quadratic term	-2.580E-07	***	2.500E-08	-2.260E-07	***	3.580E-08
Cubic term	1.070E-08	***	1.400E-09	9.700E-09	***	1.960E-09
Quartic term	-2.080E-10	***	3.500E-11	-2.020E-10	***	4.870E-11
Quintic term	1.500E-12	***	3.220E-13	1.600E-12	***	4.470E-13
			Age			
Linear term	-8.280E-07		1.220E-06	7.640E-07	***	6.090E-08
Quadratic term	4.310E-08		4.730E-08	-7.760E-09	***	4.460E-10
Cubic term	-8.870E-10		8.730E-10	-	-	-
Quartic term	8.530E-12		7.740E-12	-	-	-
Quintic term	-3.510E-14		2.650E-14	-	-	-
Female sex	7.440E-07	***	9.180E-08	-2.470E-07	*	1.110E-07
Constant term	1.717E-04	***	1.190E-05	1.377E-04	***	2.210E-06
N	286,507			308,638		
Data	PROMs-HES			PROMs-HES		
			Candidate models			
Model class	OLS, gamma-GLM with inverse link, gamma-GLM with log-link, Gaussian-GLM with log-link			OLS, gamma-GLM with inverse link, gamma-GLM with log-link, Gaussian-GLM with log-link		
OHS/OKS trend	Linear, cubic, quartic, quintic, sextic, log-linear			Linear, cubic, quartic, quintic, sextic, log-linear		
Age trend	Linear, quadratic, cubic, quartic, quintic, five-year bands, exclusion			Linear, quadratic, cubic, five-year bands, ten-year bands, exclusion		
Sex	yes/no			yes/no		

Sources: NHS PROMs and HES data, 2009-2015; own calculations.* $p<0.05$; ** $p<0.01$; *** $p<0.001$. Details on how to calculate predictions are provided in Appendix D.

For peer review only

Table C.7: Associations between pre-operative OHS/OKS and the costs of revision arthroplasty

	Hip arthroplasty			Knee arthroplasty		
Model	*gamma-GLM with inverse link function*			*OLS*		
**Variable**	**Coefficient**		**Clustered S.E.**	**Coefficient**		**Clustered S.E.**
			OHS/OKS			
Linear term	-2.370E-07	*	9.790E-08	-1.404E+00		6.816E+00
			Age			
Linear term	9.060E-07	*	3.500E-07	-2.101E+01	***	4.846E+00
Quadratic term	-6.620E-09	*	3.010E-09	-	-	-
Female sex	6.700E-06	***	1.570E-06	7.458E+01		9.551E+01
Constant term	9.570E-05	***	1.000E-05	9.111E+03	***	3.275E+02
N	2,359			3,416		
Data	PROMs-HES			PROMs-HES		
			Candidate models			
Model class	OLS, gamma-GLM with inverse link, gamma-GLM with log-link, Gaussian-GLM with log-link			OLS, gamma-GLM with inverse link, gamma-GLM with log-link, Gaussian-GLM with log-link		
OHS/OKS trend	Linear, cubic, log-linear			Linear, cubic, quartic, log-linear		
Age trend	Linear, quadratic, cubic, exclusion			Linear, quadratic, cubic, exclusion		
Sex	yes/no			yes/no		

Sources: NHS PROMs and HES data, 2009-2015; own calculations.* p<0.05; ** p<0.01; *** p<0.001. Details on how to calculate predictions are provided in Appendix D.

For peer review only

Table C.8: Associations between pre-operative OHS/OKS and costs in the 12 months leading up to TJR

	Hip arthroplasty			Knee arthroplasty		
Model	*gamma-GLM with log-link*			*gamma-GLM with log-link*		
**Variable**	**Coefficient**		**S.E.**	**Coefficient**		**S.E.**
OHS/OKS						
Log-linear term	-3.485E-01	**	1.177E-01	-	-	-
Linear term	-	-	-	4.307E-02		7.466E-02
Quadratic term	-	-	-	-1.580E-03		1.741E-03
Age						
Linear term	-8.699E-02		4.441E-02	-2.131E-02		1.430E-02
Quadratic term	5.482E-04		3.489E-04	-	-	-
Female sex	-	-	-	-	-	-
Constant term	1.033E+01	***	1.429E+00	8.027E+00	***	1.302E+00
N	441			278		
Data	COASt			COASt		
Candidate models						
Model class	OLS, Tobit censored at zero, gamma-GLM with log-link, two-part (Logit + OLS/gamma-GLM with log-link/gamma-GLM with inverse link)			OLS, Tobit censored at zero, gamma-GLM with log-link, two-part (Logit + OLS/gamma-GLM with log-link/gamma-GLM with inverse link)		
OHS/OKS trend	Linear, quadratic, cubic, log-linear			Linear, quadratic, cubic		
Age trend	Linear, quadratic, cubic, 20-year bands, exclusion			Linear, quadratic, cubic, 20-year bands, exclusion		
Sex	yes/no			yes/no		

Sources: COASt data; own calculations.* p<0.05; ** p<0.01; *** p<0.001. Details on how to calculate predictions are provided in Appendix D. For observations with OHS=0, we set ln(OHS)=0.

For peer review only

Table C.9: Associations between pre-operative OHS/OKS and ambulatory costs within 12 months after primary arthroplasty

	Hip arthroplasty			Knee arthroplasty		
Model	*OLS Regression*			*gamma-GLM with log-link*		
**Variable**	**Coefficient**		**Robust S.E.**	**Coefficient**		**Robust S.E.**
OHS/OKS						
Linear term	2.881E+01	**	8.642E+00	-1.552E-02	***	3.408E-03
Quadratic term	-1.403E+00	**	4.400E-01	-	-	-
Cubic term	1.827E-02	**	6.815E-03	-	-	-
Age						
Linear term	-2.670E+00	*	1.128E+00	-2.908E-02	***	3.060E-03
Female sex	-	-	-	-	-	-
Constant term	1.371E+02		9.342E+01	8.170E+00	***	2.203E-01
N	548			1,841		
Data	COASt			KAT		
Candidate models						
Model class	OLS, Tobit censored at zero, gamma-GLM with inverse link, gamma-GLM with log-link, Gaussian-GLM with log-link, two-part (Logit + OLS/gamma-GLM with inverse link/gamma-GLM with log-link/Gaussian-GLM with log-link)			OLS, Tobit censored at zero, gamma-GLM with inverse link, gamma-GLM with log-link, Gaussian-GLM with log-link, two-part (Logit + OLS/gamma-GLM with inverse link/gamma-GLM with log-link/Gaussian-GLM with log-link)		
OHS/OKS trend	Linear, quadratic, cubic, linear spline (20)			Linear, cubic, linear spline (25)		
Age trend	Linear, quadratic, cubic, exclusion			Linear, quadratic, cubic, log-linear, exclusion		
Sex	yes/no			yes/no		

Sources: COASt and KAT data; own calculations.* p<0.05; ** p<0.01; *** p<0.001. Details on how to calculate predictions are provided in Appendix D.

For peer review only

Table C.10: Associations between pre-operative OHS/OKS and the costs of hospital readmissions within 12 months after primary arthroplasty

	Hip arthroplasty			Knee arthroplasty		
Model	*Gaussian-GLM with log-link function*			*Gaussian-GLM with log-link function*		
**Variable**	**Coefficient**		**Clustered S.E.**	**Coefficient**		**Clustered S.E.**
	OHS/OKS					
Linear term	-1.727E-01	***	3.719E-02	-1.177E-01	***	1.342E-02
Quadratic term	1.325E-02	**	5.060E-03	3.638E-03	***	7.422E-04
Cubic term	-6.122E-04	*	2.955E-04	-4.350E-05	***	1.230E-05
Quartic term	1.370E-05		7.640E-06	-	-	-
Quintic term	-1.140E-07		7.160E-08	-	-	-
	Age					
Linear term	5.318E-03	**	1.671E-03	-8.114E-02	***	9.237E-03
Linear spline (age ≥70)	3.448E-02	***	3.360E-03	-	-	-
Quadratic term	-	-	-	7.103E-04	***	6.670E-05
Female sex	-1.785E-01	***	2.144E-02	-2.500E-01	***	2.070E-02
Constant term	6.788E+00	***	1.476E-01	9.687E+00	***	3.008E-01
N	236,514			255,194		
Data	PROMs-HES			PROMs-HES		
	Candidate models					
Model class	OLS, Tobit censored at zero, two-part (Logit + OLS/gamma-GLM with inverse link/gamma-GLM with log-link/Gaussian-GLM with log-link), gamma-GLM with inverse link, gamma-GLM with log-link, Gaussian-GLM with log-link			OLS, Tobit censored at zero, two-part (Logit + OLS/gamma-GLM with inverse link/gamma-GLM with log-link/Gaussian-GLM with log-link), gamma-GLM with inverse link, gamma-GLM with log-link, Gaussian-GLM with log-link		
OHS/OKS trend	Linear spline (10), quadratic, cubic, quartic, quintic			Linear spline (10), quadratic, cubic, quartic, quintic		
Age trend	Linear spline (70), linear, quadratic, cubic, exclusion			Linear spline (70), linear, quadratic, cubic, exclusion		
Sex	yes/no			yes/no		

Sources: NHS PROMs and HES data, 2009-2015; own calculations.* p<0.05; ** p<0.01; *** p<0.001. Details on how to calculate predictions are provided in Appendix D. The linear spline term is set to zero for patients aged below 70. For patients above 70, the term equals (age in years -70).

For peer review only

Table C.11: Associations between pre-operative OHS/OKS and annual costs of hospital readmissions between 1 and 6 years after primary arthroplasty

	Hip arthroplasty			Knee arthroplasty		
Model	*gamma-GLM with inverse link function*			*gamma-GLM with inverse link function*		
**Variable**	**Coefficient**		**Clustered S.E.**	**Coefficient**		**Clustered S.E.**
OHS/OKS						
Linear term	1.998E-04	***	1.660E-05	1.655E-04	***	8.150E-06
Current age						
Linear term	4.230E-05	***	9.000E-06	-	-	-
Age at primary arthroplasty						
Linear term	-	-	-	4.600E-05	***	5.300E-06
Time trend						
Linear term	-	-	-	1.227E-03	***	6.870E-05
Year 2 since primary surgery	-2.446E-03	***	2.263E-04	-	-	-
Female sex	-	-	-	4.290E-04	***	1.138E-04
Constant term	3.432E-03	***	7.112E-04	-3.965E-03	***	3.662E-04
N	476,514			514,047		
Data	PROMs-HES			PROMs-HES		
Candidate models						
Model class	OLS, Tobit censored at zero, two-part (Logit + OLS/gamma-GLM with inverse link/gamma-GLM with log-link/Gaussian-GLM with log-link), gamma-GLM with inverse link, gamma-GLM with log-link, Gaussian-GLM with log-link			OLS, Tobit censored at zero, two-part (Logit + OLS/gamma-GLM with inverse link/gamma-GLM with log-link/Gaussian-GLM with log-link), gamma-GLM with inverse link, gamma-GLM with log-link, Gaussian-GLM with log-link		
Year since primary surgery (time trend)	Linear, quadratic, binary indicator for year 2, binary indicators for all years, exclusion			Linear, quadratic, binary indicator for year 2, binary indicators for all years, exclusion		
OHS/OKS trend	Linear, quadratic, cubic, quartic, quintic, log-linear			Linear, quadratic, cubic, quartic, quintic, log-linear		
Age trend	Age at operation vs. current age; for current age: Linear, quadratic, cubic, five-year age bands, exclusion			Age at operation vs. current age; for age at operation: Linear, quadratic, cubic, quartic, quintic, five-year age bands, exclusion		
Sex	yes/no			yes/no		

Sources: NHS PROMs and HES data, 2009-2015; own calculations.* p<0.05; ** p<0.01; *** p<0.001. Details on how to calculate predictions are provided in Appendix D. "Year 2 since primary surgery" is a binary indicator. "Year 1" refers to the first 12 months after primary surgery.

For peer review only

Table C.12: Associations between pre-operative OHS/OKS and the costs of hospital readmissions during the year of revision surgery

	Hip arthroplasty			Knee arthroplasty		
Model	*Two-part model: Logit + gamma-GLM with inverse link*			*Two-part model: Logit + gamma-GLM with log-link*		
**Variable**	**Coefficient**		**Clustered S.E.**	**Coefficient**		**Clustered S.E.**
			**Part 1: Logit model**			
			OHS/OKS			
Linear term	8.407E-03		1.093E-02	3.790E-03		8.559E-03
			Age at primary arthroplasty			
Linear term	9.589E-02	*	4.418E-02	5.140E-04		6.716E-03
Quadratic term	-8.234E-04	*	3.622E-04	-	-	-
			Time trend			
Linear term	-	-	-	-2.488E+00		1.438E+00
Quadratic term	-	-	-	6.157E-01		3.933E-01
Cubic term	-	-	-	-4.586E-02		3.340E-02
Year 2 since primary surgery	3.153E-01		1.675E-01	-	-	-
Female sex	1.724E-01		1.624E-01	2.386E-01		1.235E-01
Constant term	-8.202E-01		1.353E+00	4.622E+00	**	1.699E+00
			**Part 2: gamma-GLM**			
			OHS/OKS			
Linear term	-1.340E-06		9.040E-07	-1.291E-02		7.915E-03
			Age at primary arthroplasty			
Linear term	-9.550E-07		5.400E-06	1.018E-02		5.999E-03
Quadratic term	-4.800E-09		4.080E-08	-	-	-
			Time trend			
Linear term	-	-	-	-3.169E-01		1.725E+00
Quadratic term	-	-	-	1.474E-01		4.819E-01
Cubic term	-	-	-	-1.556E-02		4.192E-02
Year 2 since primary surgery	4.800E-05	**	1.760E-05	-	-	-
Female sex	5.210E-05	*	2.040E-05	-9.075E-02		1.228E-01
Constant term	2.078E-04		1.764E-04	8.352E+00	***	1.982E+00
N	1,669			2,258		
Data	PROMs-HES			PROMs-HES		

...continued on next page

For peer review only

	Candidate models	
Model class	OLS, Tobit censored at zero, two-part (Logit + OLS/gamma-GLM with inverse link/gamma-GLM with log-link/Gaussian-GLM with log-link), gamma-GLM with inverse link, gamma-GLM with log-link, Gaussian-GLM with log-link	OLS, Tobit censored at zero, two-part (Logit + OLS/gamma-GLM with inverse link/gamma-GLM with log-link/Gaussian-GLM with log-link), gamma-GLM with inverse link, gamma-GLM with log-link, Gaussian-GLM with log-link
Year since primary surgery (time trend)	Binary indicator for year 2, exclusion	Linear, quadratic, cubic, exclusion
OHS/OKS trend	Linear, quadratic, cubic	Linear, quadratic, cubic
Age trend	Age at operation vs. current age; for age at operation: Linear, quadratic, cubic, five-year age bands, exclusion	Age at operation vs. current age; for age at operation: Linear, quadratic, cubic, five-year age bands, exclusion
Sex	yes/no	yes/no

Sources: NHS PROMs and HES data, 2009-2015; own calculations.* p<0.05; ** p<0.01; *** p<0.001. Details on how to calculate predictions are provided in Appendix D. "Year 2 since primary surgery" is a binary indicator. "Year 1" refers to the first 12 months after primary surgery.

For peer review only

Table C.13: Associations between pre-operative OHS/OKS and annual costs of hospital readmissions for patient with a previous revision

	Hip arthroplasty			Knee arthroplasty		
Model	*Tobit model censored at zero*			*Tobit model censored at zero*		
**Variable**	**Coefficient**		**Clustered S.E.**	**Coefficient**		**Clustered S.E.**
			OHS/OKS			
Linear term	-2.424E+02	*	1.074E+02	-1.972E+02	*	9.961E+01
			Current age			
Linear term	-	-	-	-1.172E+04	*	4.527E+03
Quadratic term	-	-	-	1.772E+02	*	7.058E+01
Cubic term	-	-	-	-8.911E-01	*	3.609E-01
			Time trend			
Linear term	-	-	-	-1.935E+03	***	4.973E+02
Constant term	-2.454E+04	***	3.657E+03	2.455E+05	**	9.386E+04
σ (Tobit term)	1.472E+04	-	1.859E+03	1.390E+04		2.139E+03
N	2,406			3,153		
Data	PROMs-HES			PROMs-HES		
			Candidate models			
Model class	OLS, Tobit censored at zero, two-part (Logit + OLS/gamma-GLM with inverse link/gamma-GLM with log-link/Gaussian-GLM with log-link), gamma-GLM with inverse link, gamma-GLM with log-link, Gaussian-GLM with log-link			OLS, Tobit censored at zero, two-part (Logit + OLS/gamma-GLM with inverse link/gamma-GLM with log-link/Gaussian-GLM with log-link), gamma-GLM with inverse link, gamma-GLM with log-link, Gaussian-GLM with log-link		
Year since primary surgery (time trend)	Linear, quadratic, exclusion			Linear, quadratic, cubic, exclusion		
OHS/OKS trend	Linear, quadratic, cubic			Linear, quadratic, cubic, quartic, quintic, log-linear		
Age trend	Age at operation vs. current age; for age at operation: linear, quadratic, cubic, five-year age bands, exclusion			Age at operation vs. current age; forcurrent age: linear, quadratic, cubic, exclusion		
Sex	yes/no			yes/no		

Sources: NHS PROMs and HES data, 2009-2015; own calculations.* p<0.05; ** p<0.01; *** p<0.001. Details on how to calculate predictions are provided in Appendix D.

For peer review only

Table C.14: Associations between pre-operative OKS and annual ambulatory costs between 1 and 12 years after TKR

	**Knee arthroplasty**		
Model	*Gaussian-GLM with log-link function*		
**Variable**	**Coefficient**		**Clustered S.E.**
	OKS		
Linear term	1.125E-03		9.113E-04
Quadratic term	-5.597E-01	*	2.420E-01
	Age at primary operation		
Linear term	3.672E-01		2.467E-01
Quadratic term	-7.167E-03		4.043E-03
Cubic term	4.130E-05		2.150E-05
	Year since primary surgery (time trend)		
Linear term	-5.597E-01	*	2.420E-01
Quadratic term	5.295E-02		4.104E-02
Cubic term	-1.810E-03		2.111E-03
Female sex	-3.197E-01	*	1.363E-01
Constant term	1.212E+00		4.801E+00
N	13,271		
Data	KAT		
	Candidate models		
Model class	OLS, Tobit censored at zero, two-part (Logit + OLS/gamma-GLM with log-link), gamma-GLM with inverse link, gamma-GLM with log-link, Gaussian-GLM with log-link		
Time trend	Linear, quadratic, cubic trend, binary indicator for year 2, binary indicator for year 2 and a linear trend, exclusion		
OKS trend	Linear, quadratic, cubic		
Age trend	Age at operation vs. current age; for age at operation: Linear, quadratic, cubic, exclusion		
Sex	yes/no		

Sources: KAT data; own calculations.* p<0.05; ** p<0.01; *** p<0.001. Details on how to calculate predictions are provided in Appendix D. The model was estimated on data in long-format (i.e., one observation per patient and year). The inclusion of the time trend was tested after selecting the model class and before choosing a trend for OKS. We did not estimate a model for hips due to a lack of data.

For peer review only

Table C.15: Associations between pre-operative OKS and ambulatory costs in the year of revision surgery

	Knee arthroplasty		
Model	*gamma-GLM with log-link function*		
**Variable**	**Coefficient**		**Clustered S.E.**
	OKS		
Linear term	-3.271E-02		1.805E-02
	Age at primary operation		
Linear term	2.062E-01	*	8.868E-02
Quadratic term	-1.777E-03	**	6.774E-04
Constant term	1.145E+00		2.861E+00
N	88		
Data	KAT		
	Candidate models		
Model class	OLS, Tobit censored at zero, two-part (Logit + OLS/gamma-GLM with log-link/gamma-GLM with inverse link/Gaussian-GLM with log-link), gamma-GLM with inverse link, gamma-GLM with log-link, Gaussian-GLM with log-link		
OKS trend	Linear, quadratic, cubic		
Age trend	Linear, quadratic, cubic, exclusion		
Sex	yes/no		

Sources: KAT data; own calculations.* p<0.05; ** p<0.01; *** p<0.001. Details on how to calculate predictions are provided in Appendix D. The model was estimated on data in long-format (i.e., one observation per patient and year). We did not estimate a model for hips due to a lack of data.

For peer review only

Table C.16: Associations between pre-operative OKS and annual ambulatory costs for patients with a previous revision

	Knee arthroplasty		
Model	*gamma-GLM with inverse link function*		
**Variable**	**Coefficient**		**Clustered S.E.**
	OKS		
Linear term	1.637E-03		9.185E-04
Quadratic term	-1.439E-04		7.930E-05
Cubic term	3.860E-06	*	1.890E-06
Female sex	1.726E-03		2.068E-03
Constant term	3.737E-04		1.859E-03
N	329		
Data	KAT		
	Candidate models		
Model class	OLS, Tobit censored at zero, two-part (Logit + OLS/gamma-GLM with log-link/gamma-GLM with inverse link/Gaussian-GLM with log-link), gamma-GLM with inverse link, gamma-GLM with log-link, Gaussian-GLM with log-link		
OKS trend	Linear, quadratic, cubic		
Age trend	Linear, quadratic, cubic, exclusion		
Sex	yes/no		

Sources: KAT data; own calculations.* p<0.05; ** p<0.01; *** p<0.001. Details on how to calculate predictions are provided in Appendix D. Notes: The model was estimated on data in long-format (i.e., one observation per patient and year). We did not estimate a model for hips due to a lack of data.

For peer review only

D. Note on calculating predictions

1) OLS models

The predicted value of the outcome variable, $\hat{y}$ is simply the linear predictor Xb, which is obtained by multiplying each variable with its estimated coefficient and then summing over all variables.

Example:

For a patient aged 60 and with an OHS of 18, the ambulatory costs in the first 12 months after primary THR (Table C.9) can be predicted as:

$$\begin{aligned}\cos t &= 28.81 \times OHS - 1.40 \times OHS^2 + 0.02 \times OHS^3 - 2.67 \times age + 137.10 \\ &= 28.81 \times 18 - 1.40 \times 324 + 0.02 \times 5832 - 2.67 \times 60 + 137.10 \\ &= 147.46\end{aligned}$$

2) Gamma-GLM with an inverse link function

Predicted values for the outcome variable can be obtained as $\hat{y} = \frac{1}{Xb}$.

Example:

For a female patient with OKS of 15, the annual ambulatory costs after revision TKR surgery (Table C.16) can be calculated as follows:

$$\begin{aligned}\cos t &= \frac{1}{0.000374 + 0.001726 \times female + 0.001637 \times OKS - 0.000144 \times OKS^2 + 0.000004 \times OKS^3} \\ &= \frac{1}{0.000374 + 0.001726 \times 1 + 0.001637 \times 15 - 0.000144 \times 225 + 0.000004 \times 3375^3} \\ &= \frac{1}{0.007305} \\ &= 136.90\end{aligned}$$

3) Gamma-GLM with a log-link function

Predicted values for the outcome variable can be obtained as $\hat{y} = e^{Xb}$.

For peer review only - http://bmjopen.bmj.com/site/about/guidelines.xhtml

Example: For a patient aged 60 at the time of her primary TKR operation and with an OKS of 10, the ambulatory cost in the year of revision surgery (Table C.15) can be calculated as follows:

$$\begin{aligned} \cos t &= e^{1.145-0.033\times OKS+0.206\times age-0.002\times age^2} \\ &= e^{1.145-0.033\times 10+0.206\times 60-0.002\times 3600} \\ &= e^{6.7927} \\ &= 2.7182^{6.7927} \\ &= 891.32 \end{aligned}$$

For peer review only

4) Gaussian-GLM with a log-link functio

Predicted values for the outcome variable can be obtained as $\hat{y} = e^{Xb}$.

Example: For a female patient aged 70 and with an OKS of 70, the cost of hospital readmission in the first 12 months after primary TKR (Table C.10) can be calculated as follows:

$$\begin{aligned} \cos t &= e^{9.68700-0.25\times female-0.11770\times OKS+0.00364\times OKS^2-0.00004\times OKS^3-0.08114\times age+0.00071\times age^2} \\ &= e^{9.68700-0.25\times 1-0.11770\times 20+0.00364\times 400-0.00004\times 8000-0.08114\times 70+0.00071\times 4900} \\ &= e^{5.99087} \\ &= 2.7182^{5.99087} \\ &= 399.76 \end{aligned}$$

5) Tobit models censored at zero

The predicted value is given by $\hat{y} = -\Phi\left(\frac{Xb}{\sigma}\right)Xb + \sigma\phi\left(\frac{Xb}{\sigma}\right)$

where $\Phi(\cdot)$ denotes the cumulative distribution function of the standard normal distribution, and $\phi(\cdot)$ denotes the probability density function of the standard normal distribution.

Example: For a patient with an OHS of 25, the annual cost of hospital readmissions after revision surgery (Table C.13) can be calculated as follows:

$$\cos t = -\Phi\left(\frac{-24540 - 242.40 \times OHS}{14720}\right)(-24540 - 242.40 \times OHS) + 14720\phi\left(\frac{-24540 - 242.40 \times OHS}{14720}\right)$$
$$= -\Phi\left(\frac{-24540 - 242.40 \times 20}{14720}\right)(-24540 - 242.40 \times 20) + 14720\phi\left(\frac{-24540 - 242.40 \times 20}{14720}\right)$$
$$= -\Phi(-1.99647)(-29388) + 14720\phi(-1.99647)$$
$$= 0.022942 \times (-29388) + 14720 \times 0.054373$$
$$= 126.15$$

6) Tobit models censored at -0.594 and 1

The predicted value is given by

$$\hat{y} = -0.594 * \Phi\left(\frac{-0.594 - Xb}{\sigma}\right) + 1 * \Phi\left(-\frac{1 - Xb}{\sigma}\right)$$
$$+\left(\Phi\left(\frac{1 - Xb}{\sigma}\right) - \Phi\left(\frac{-0.594 - Xb}{\sigma}\right)\right) * \left(Xb - \sigma * \frac{\phi\left(\frac{1 - Xb}{\sigma}\right) - \phi\left(\frac{-0.594 - Xb}{\sigma}\right)}{\Phi\left(\frac{1 - Xb}{\sigma}\right) - \Phi\left(\frac{-0.594 - Xb}{\sigma}\right)}\right)$$

where $\Phi(\cdot)$ denotes the cumulative distribution function of the standard normal distribution, and $\phi(\cdot)$ denotes the probability density function of the standard normal distribution.

Example: For a patient aged 75 in the year of THR revision surgery and with a pre-operative (pre-primary) OHS of 8, the EQ-5D utility before revision surgery (Table C.4) can be calculated with the following steps.

First, the linear predictor (*Xb*) is calculated as:

$$Xb = -0.1444 + 0.0021 \times currentage + 0.0267 \times OHS - 0.0003 \times OHS^2$$
$$= -0.1444 + 0.0021 \times 75 + 0.0267 \times 8 - 0.0003 \times 64$$
$$= 0.21$$

Then, using this value and the formula above the predicted EQ-5D utility can be calculated as follows:

$$QoL = -0.594 \times \Phi\left(\frac{-0.594-0.21}{0.3178}\right) + 1 \times \Phi\left(-\frac{1-0.21}{0.3178}\right)$$

$$+\left(\Phi\left(\frac{1-0.21}{0.3178}\right) - \Phi\left(\frac{-0.594-0.21}{0.3178}\right)\right) \times \left(0.21 - 0.3178 \times \frac{\phi\left(\frac{1-0.21}{0.3178}\right) - \phi\left(\frac{-0.594-0.21}{0.3178}\right)}{\Phi\left(\frac{1-0.21}{0.3178}\right) - \Phi\left(\frac{-0.594-0.21}{0.3178}\right)}\right)$$

$$= -0.594 \times 0.005705 + 1 \times 0.006462 + (0.993538 - 0.005705) \times \left(0.21 - 0.3178 \times \frac{0.018158 - 0.016259}{0.993538 - 0.005705}\right)$$

$$= -0.00339 + 0.006462 + 0.987833 \times (0.21 - 0.000611)$$

$$= 0.209913$$

For peer review only

7) Two-part models

The predicted value from a two-part model is given by $\hat{y} = (1 - P[y=0 \mid X]) * E[y \mid y>0, X]$.

$E[y \mid y>0, X]$ is given by the predicted value in the second part of the model.

$P[y=0 \mid X]$ is given as $P[y=0 \mid X] = \frac{e^{Xb}}{1+e^{Xb}}$ if the first part of the model is a logit model.

Example: For a female patient aged 65 in the year of her primary THR operation and with an OHS of 15, the costs of hospital readmissions in the year of revision surgery in year 3 since her primary operation (Table C.12) can be calculated with the following steps:

First, we calculate the linear predictor, *Xb*, of the first part of the model:

$$Xb_1 = -0.820 + 0.172 \times female + 0.315 \times year2 + 0.008 \times OHS + 0.096 \times age - 0.001 \times age^2$$
$$= -0.820 + 0.172 \times 1 + 0.315 \times 0 + 0.008 \times 15 + 0.096 \times 65 - 0.001 \times 4225$$
$$= 2.23229$$

Then, the probability of zero cost can be calculated as follows:

$$P[\cos t = 0 \mid Xb] = \frac{e^{Xb}}{1+e^{Xb}}$$
$$= \frac{e^{2.23229}}{1+e^{2.23229}}$$
$$= \frac{2.718281^{2.23229}}{1+2.718281^{2.23229}}$$
$$= \frac{9.321187}{1+9.321187}$$
$$= 0.903112$$

Next, the linear predictor of the second part of the model is calculated as:

$$\begin{aligned} Xb_2 &= 0.0002078 + 0.0000521 \times female + 0.000048 \times year2 - 0.00000134 \times OHS \\ &\quad - 0.000000955 \times age - -0.000000005 \times age^2 \\ &= 0.0002078 + 0.0000521 \times 1 + 0.000048 \times 0 - 0.00000134 \times 15 \\ &\quad - 0.000000955 \times 65 - -0.000000005 \times 4225 \\ &= 0.000157 \end{aligned}$$

Then, the predicted costs of the second part of the model can be calculated as:

$$\begin{aligned} E\left[\cos t \mid \cos t > 0, Xb\right] &= \frac{1}{Xb} \\ &= \frac{1}{0.000157} \\ &= 6351.424 \end{aligned}$$

The formula used here is the prediction formula for a gamma-GLM with an inverse link function (see section 2), since this is the model used for the second part in Table C.12. The first part of the model predicts that for the patient described in this example the probability of incurring no cost for hospital readmissions in the year of revision surgery is 90.3%. The second part predicts that if the patient has a hospital admission, than the predicted annual costs for readmissions are £6351.4.

These two results can be combined to derive the predicted cost of hospital readmissions as follows:

$$\begin{aligned} \cos t &= \left(1 - P\left[y = 0 \mid X\right]\right) * E\left[y \mid y > 0, X\right] \\ &= (1 - 0.903112) * 6351.424 \\ &= 615.38 \end{aligned}$$

This values takes into account that there is a high probability that the cost are zero.

For peer review only

STROBE 2007 (v4) checklist of items to be included in reports of observational studies in epidemiology*

Checklist for cohort, case-control, and cross-sectional studies (combined)

Section/Topic	Item #	Recommendation	Reported on page #
**Title and abstract**	1	(*a*) Indicate the study's design with a commonly used term in the title or the abstract	p.1/2
		(*b*) Provide in the abstract an informative and balanced summary of what was done and what was found	p.2
**Introduction**			
Background/rationale	2	Explain the scientific background and rationale for the investigation being reported	p.4
Objectives	3	State specific objectives, including any pre-specified hypotheses	p.4
**Methods**			
Study design	4	Present key elements of study design early in the paper	pp. 4-11
Setting	5	Describe the setting, locations, and relevant dates, including periods of recruitment, exposure, follow-up, and data collection	p.4/5
Participants	6	(*a*) *Cohort study*—Give the eligibility criteria, and the sources and methods of selection of participants. Describe methods of follow-up<br>*Case-control study*—Give the eligibility criteria, and the sources and methods of case ascertainment and control selection. Give the rationale for the choice of cases and controls<br>*Cross-sectional study*—Give the eligibility criteria, and the sources and methods of selection of participants	p.5
		(*b*) *Cohort study*—For matched studies, give matching criteria and number of exposed and unexposed<br>*Case-control study*—For matched studies, give matching criteria and the number of controls per case	
Variables	7	Clearly define all outcomes, exposures, predictors, potential confounders, and effect modifiers. Give diagnostic criteria, if applicable	pp.5-8
Data sources/ measurement	8*	For each variable of interest, give sources of data and details of methods of assessment (measurement). Describe comparability of assessment methods if there is more than one group	*pp.5-8*
Bias	9	Describe any efforts to address potential sources of bias	p. 10/11
Study size	10	Explain how the study size was arrived at	NA
Quantitative variables	11	Explain how quantitative variables were handled in the analyses. If applicable, describe which groupings were chosen and why	pp.5-11
Statistical methods	12	(*a*) Describe all statistical methods, including those used to control for confounding	p.10/11
		(*b*) Describe any methods used to examine subgroups and interactions	p.10/11
		(*c*) Explain how missing data were addressed	p.10/11
		(*d*) *Cohort study*—If applicable, explain how loss to follow-up was addressed<br>*Case-control study*—If applicable, explain how matching of cases and controls was addressed	NA

For peer review only

		Cross-sectional study—If applicable, describe analytical methods taking account of sampling strategy	
		(*e*) Describe any sensitivity analyses	p.10/11
**Results**			
Participants	13*	(a) Report numbers of individuals at each stage of study—eg numbers potentially eligible, examined for eligibility, confirmed eligible, included in the study, completing follow-up, and analysed	p.4/5, Table 1
		(b) Give reasons for non-participation at each stage	NA
		(c) Consider use of a flow diagram	NA
Descriptive data	14*	(a) Give characteristics of study participants (eg demographic, clinical, social) and information on exposures and potential confounders	Table 1
		(b) Indicate number of participants with missing data for each variable of interest	Table
		(c) *Cohort study*—Summarise follow-up time (eg, average and total amount)	NA
Outcome data	15*	*Cohort study*—Report numbers of outcome events or summary measures over time	
		Case-control study—Report numbers in each exposure category, or summary measures of exposure	
		Cross-sectional study—Report numbers of outcome events or summary measures	Table 1
Main results	16	(*a*) Give unadjusted estimates and, if applicable, confounder-adjusted estimates and their precision (eg, 95% confidence interval). Make clear which confounders were adjusted for and why they were included	supplementary material
		(*b*) Report category boundaries when continuous variables were categorized	supplementary material
		(*c*) If relevant, consider translating estimates of relative risk into absolute risk for a meaningful time period	NA
Other analyses	17	Report other analyses done—eg analyses of subgroups and interactions, and sensitivity analyses	
**Discussion**			
Key results	18	Summarise key results with reference to study objectives	p. 16
Limitations	19	Discuss limitations of the study, taking into account sources of potential bias or imprecision. Discuss both direction and magnitude of any potential bias	p. 17/18
Interpretation	20	Give a cautious overall interpretation of results considering objectives, limitations, multiplicity of analyses, results from similar studies, and other relevant evidence	p. 16/17
Generalisability	21	Discuss the generalisability (external validity) of the study results	p. 16
**Other information**			
Funding	22	Give the source of funding and the role of the funders for the present study and, if applicable, for the original study on which the present article is based	p. 19/20

*Give information separately for cases and controls in case-control studies and, if applicable, for exposed and unexposed groups in cohort and cross-sectional studies.

For peer review only

Note: An Explanation and Elaboration article discusses each checklist item and gives methodological background and published examples of transparent reporting. The STROBE checklist is best used in conjunction with this article (freely available on the Web sites of PLoS Medicine at http://www.plosmedicine.org/, Annals of Internal Medicine at http://www.annals.org/, and Epidemiology at http://www.epidem.com/). Information on the STROBE Initiative is available at www.strobe-statement.org.

For peer review only

BMJ Open

Association between pre-operative Oxford hip and knee scores and costs and quality-of-life of patients undergoing primary total joint replacement in the NHS England: An observational study

Journal:	*BMJ Open*
Manuscript ID	bmjopen-2017-019477.R2
Article Type:	Research
Date Submitted by the Author:	06-Feb-2018
Complete List of Authors:	Eibich, Peter; University of Oxford, Health Economics Research Centre, Nuffield Department of Population Health; Max Planck Institute for Demographic Research<br>Dakin, Helen; University of Oxford, Health Economics Research Centre, Nuffield Department of Population Health<br>Price, Andrew; University of Oxford, Nuffield Department of Orthopaedics, Rheumatology and Musculoskeletal Sciences<br>Beard, David; University of Oxford, Nuffield Dept of Orthopaedics, Rheumatology and Musculoskeletal Sciences<br>Arden, Nigel; Nuffield Department of Orthopaedics, ;<br>Gray, Alastair; University of Oxford, Nuffield Department of Population Health
**Primary Subject Heading**:	Rheumatology
Secondary Subject Heading:	Health policy
Keywords:	hip replacement, knee replacement, costs, quality-of-life, Oxford Hip Score, Oxford Knee Score

SCHOLARONE™
Manuscripts

Association between pre-operative Oxford hip and knee scores and costs and quality-of-life of patients undergoing primary total joint replacement in the NHS England: An observational study

Peter Eibich[1,2], Helen Dakin[1], Andrew Price[3], David Beard[3], Nigel Arden[3,4] and Alastair M. Gray[1]

[1] Health Economics Research Centre, Nuffield Department of Population Health, University of Oxford, Oxford, UK

[2] Max Planck Institute for Demographic Research, Rostock, Germany

[3] Nuffield Department of Orthopaedics, Rheumatology and Musculoskeletal Sciences, University of Oxford, Oxford, UK

[4] Faculty of Medicine, University of Southampton, Southampton, UK

Running title: Associations between OHS/OKS and costs and quality-of-life

5912 words

3 tables

3 figures

Corresponding author:

Peter Eibich, Max Planck Institute for Demographic Research, Konrad-Zuse-Str. 1, 18057 Rostock, Germany. Tel.: +49 (0)381 2081-220. E-Mail: eibich@demogr.mpg.de.

For peer review only - http://bmjopen.bmj.com/site/about/guidelines.xhtml

Abstract

Objectives: To assess how costs and quality-of-life (measured by EQ-5D) before and after total hip and knee replacement (THR/TKR) vary with age, gender and pre-operative Oxford hip and knee scores (OHS/OKS).

Design: Regression analyses using prospectively collected data from clinical trials, cohort studies and administrative data bases.

Setting: UK secondary care.

Participants: Men and women undergoing primary THR or TKR. The Hospital Episode Statistics data linked to patient reported outcome measures included 602,176 patients undergoing hip or knee replacement who were followed up for up to six years. The Knee Arthroplasty Trial included 2,217 TKR patients who were followed up for 12 years. The Clinical Outcomes in Arthroplasty Study cohort included 806 THR and 484 TKR patients who were observed for one year.

Outcome measures: EQ-5D-3L quality-of-life before and after surgery, costs of primary arthroplasty, costs of revision arthroplasty and the costs of hospital readmissions and ambulatory costs in the year before and up to 12 years after joint replacement.

Results: Average post-operative utility for patients at the 5th percentile of the OHS/OKS distribution was 0.61/0.5 for THR/TKR, and 0.89/0.85 for patients at the 95th percentile. The difference between post- and pre-operative EQ-5D utility was highest for patients with pre-operative OHS/OKS lower than 10. However, post-operative EQ-5D utility was higher than pre-operative utility for all patients with OHS ≤46 and those with OKS ≤44. In contrast, costs were generally higher for patients with low pre-operative OHS/OKS than those with high OHS/OKS. For example, costs of hospital readmissions within 12 months after primary THR/TKR were £740/£888 for patients at the 5th percentile compared to £314/£404 at the 95th percentile of the OHS/OKS distribution.

Conclusions: Our findings suggest that costs and quality-of-life associated with total joint replacement vary systematically with pre-operative symptoms measured by OHS/OKS.

293 words

Keywords: Total joint replacement, hip replacement, knee replacement, costs, quality-of-life, Oxford Hip Score, Oxford Knee Score, regression model.

For peer review only - http://bmjopen.bmj.com/site/about/guidelines.xhtml

Strengths and limitations of this study

- This study used data from different sources, including clinical trials, cohort studies and administrative databases.
- The sample sizes are considerably larger than those used in previous studies.
- The optimal regression models were identified in a systematic model selection process designed to maximise predictive accuracy.
- Our largest dataset contained no information on potentially important variables such as body mass index.

For peer review only

For peer review only - http://bmjopen.bmj.com/site/about/guidelines.xhtml

INTRODUCTION

Total joint replacement (TJR) has been shown to greatly improve the quality-of-life of patients with osteoarthritis.[1–5] Moreover, previous studies have demonstrated that both total knee replacement (TKR) and total hip replacement (THR) are highly cost-effective.[3,4] Nevertheless, in the UK access to TJR surgery is frequently rationed based on patient characteristics (such as age and functional status),[6] since it is a non-urgent procedure with a high impact on healthcare budgets. In particular, the Oxford hip and knee scores (OHS/OKS) were used in certain regions to define threshold values (varying from 19 to 30) above which patients are not eligible for TJR.[7–10] This is based on the argument that patients with lower pre-operative functional status benefit more from TJR, but the evidence to support this is limited, and very few studies have examined how outcomes or costs of TJR differ by pre-operative patient characteristics.[1–5,11–16]

Most of these studies focused on demographic characteristics such as age, with only five studies exploring the impact of pre-operative function.[3,4,13,14,16] Cushnaghan et al.[13] examined associations between baseline physical functioning (measured by SF-36) and the long-term outcome of TKR (measured by the change in SF-36). Judge et al.[16] investigated associations between pre-operative OHS and post-operative OHS and concluded that all patients benefitted substantially from THR. Only Dakin et al.[4] and Fordham et al.[3] investigated the impact of OKS and OHS on costs and outcomes of TKR and TJR, respectively, and both studies concluded that while health benefits and costs do vary with pre-operative OHS/OKS score, TJR improves quality-of-life for almost all patients.

One recent US study by Ferket et al.[14] concluded that TKR results in only minimal health gains for patients with good physical function (measured by the SF-12 physical score), and that, on economic grounds, access to TKR should be restricted to patients with severe symptoms; however this study was based on only 382 TKR patients. Other studies in this area have also relied on small datasets (e.g., 2,138 TKR[4] or 938 THR patients[3] followed for five years), which are likely to contain very few patients with low or high scores. The results from these studies are therefore subject to considerable uncertainty.

This study aimed to estimate the relationship between pre-operative patient characteristics (age, sex and OHS/OKS) and the costs and quality-of-life of patients undergoing TJR in England. We used administrative data from the NHS patient reported outcome measures (PROMs) programme linked to Hospital Episode Statistics (HES) data, alongside smaller datasets from the Knee Arthroplasty Trial (KAT) and the Clinical Outcomes in Arthroplasty Study (COASt) cohort study to obtain reliable estimates on a wide range of outcomes and patients, including for patients with very high OHS/OKS.

METHODS

Datasets

The study focused on patients undergoing elective primary hip or knee arthroplasty from three data sources –the KAT trial,[17] the COASt cohort study,[18] and data from the NHS PROMs programme linked to HES data.[19] These datasets were chosen from a wider range

For peer review only - http://bmjopen.bmj.com/site/about/guidelines.xhtml

of available candidate datasets[3,20–22] based on the availability of relevant outcome and baseline data as well as sample size considerations.

The NHS PROMs programme routinely collects data on all patients undergoing hip and knee replacement on the NHS in England.[23] Data are collected via questionnaires at baseline (typically the day of admission for surgery) and at minimum six months post-surgery. The dataset used in this study covered the period from April 2009 to October 2015. We linked these data to Admitted Patient Care data from HES,[19] which contains routine data on all patients treated in hospitals in the NHS England, with a particular focus on diagnoses and procedures. This yielded a sample of up to 602,176 patients who were followed up for up to six years. The data include patients undergoing primary joint replacement surgery other than TJR (e.g., hip resurfacing), since we were not able to reliably identify the type of operation from the available data.

KAT is a randomised controlled trial comparing different types of knee prosthesis.[17] We used data on EQ-5D utility and ambulatory costs following 2217 UK patients, who were followed up annually for up to 12 years after TKR.

COASt[18] is a prospective, dual-centre longitudinal cohort study recruiting patients across two hospitals: University Hospital Southampton NHS Foundation Trust and Nuffield Orthopaedic Centre (part of Oxford University Hospitals NHS Trust). COASt was established in 2010 and recruited patients placed on the waiting list for knee or hip replacement surgery. The datasets extracted for this study contained observations for 810 hip surgery patients and 858 patients undergoing knee surgery. After excluding patients who underwent procedures other than THR/TKR (e.g., partial knee replacement or hip resurfacing), the datasets contained 806 THR patients and 484 TKR patients. Data are collected prior to surgery as well as at six weeks and then annually for five years thereafter, although only the first year of data were used in this study.

Outcomes

Quality-of-life

Pre- and post-operative quality-of-life was measured using EQ-5D-3L, valued using the UK time trade-off tariff.[24] EQ-5D-3L health utility takes values between -0.594 and 1, with higher values representing higher quality-of-life. We chose to focus on EQ-5D, since it is the preferred measure of quality-of-life for economic evaluations submitted to the National Institute for Health and Care Excellence (NICE).

Linked NHS PROMs/HES data were used to estimate quality-of-life before surgery and six months after THR or TKR. Data from KAT were used to estimate annual changes in quality-of-life between six months and 12 years after TKR.

Costs

Primary and revision arthroplasty

For peer review only - http://bmjopen.bmj.com/site/about/guidelines.xhtml

We estimated the impact of OHS/OKS on the costs of primary and revision TJR using linked NHS PROMs/HES data. We identified HES observations related to primary or revision arthroplasty by linking NHS PROMs data to the HES database, and then excluded all observations marked as revisions in the corresponding NHS PROMs questionnaire. If a patient was observed to have the same procedure (THR or TKR) carried out at the same joint (right- or left-side) more than once, we assumed that the earliest observed procedure was a primary surgery episode, while all subsequent procedures were revision episodes. Similarly, we identified revisions as those episodes that were either marked as a revision in the NHS PROMs data, or where we observed an earlier procedure on the same joint in the HES data. This implies that for patients undergoing primary joint replacement other than TJR first and TJR on the same joint later, the first procedure would be coded as primary surgery and the second procedure would be coded as a revision, since we were not able to reliably identify the type of operation based on the available data.

Readmissions after primary and revision arthroplasty

We derived the costs of hospital readmissions after primary arthroplasty using PROMs/HES data. Our HES data extract included all hospital admissions for patients contained in the NHS PROMs data (i.e., who underwent primary or revision arthroplasty between April 2009 and October 2015). In a first step, we split the data into two datasets, one consisting of all “index” (i.e., primary or revision arthroplasty) episodes and one containing all other episodes. Then we matched the non-index admissions to the index episodes. If a hospital admission could be matched to more than one index episode, we matched the admission to the closest arthroplasty episode that occurred before the hospital admission. However, we excluded arthroplasty episodes from the match if the hospital admission referred to a different joint or side of the body than the arthroplasty episode.

To determine whether a matched hospital admission can be attributed to primary or revision arthroplasty, we applied the following set of criteria:

- The admission occurred within 30 days after primary arthroplasty.
- The admission had a primary diagnosis for hip or knee arthritis.
- The admission had a procedure code referring to the hip or knee joint.
- The admission had a diagnosis code associated with infections of the skin, the joint or the prosthesis.

These criteria were developed based on discussions with a clinical registrar and a clinical coding manager. The detailed diagnosis and procedure codes are available in the online supplementary file (section A). Any admission that met at least one of the criteria was retained as a relevant episode.

Finally, we aggregated all readmissions by year from primary arthroplasty to create an annual measure of readmissions for each observed primary arthroplasty procedure. We assumed that readmissions were zero for those patients known to be alive with no recorded admissions in any given year. The resulting dataset contains observations for all patients (including those without hospital readmissions) and all years for up to six years following primary

For peer review only - http://bmjopen.bmj.com/site/about/guidelines.xhtml

arthroplasty. Patients were considered to be observed either until the end of the study period (October 2015), or until their death if they died in hospital. Deaths outside of the hospital were not observed in the data. We analysed readmission costs in the first 12 months after primary arthroplasty separately from annual readmission costs between one and six years after surgery. We also conducted a separate analysis of readmission costs in the year of revision surgery and annual readmission costs after revision surgery.

Ambulatory costs after primary arthroplasty and costs before primary arthroplasty

Ambulatory costs included outpatient and community costs related to primary arthroplasty, e.g., GP visits, nurse visits, physiotherapy, outpatient appointments at a hospital or visits to the "Accidents & Emergency" department. We excluded costs such as medication, nursing home care, personal care, and equipment or home modifications due to a lack of data.

For THR, data on ambulatory costs in the first 12 months after primary arthroplasty were obtained from the COASt study, but annual ambulatory costs more than 12 months after primary hip arthroplasty could not be analysed due to a lack of data. For TKR, we used data on annual ambulatory costs from the KAT trial for up to 12 years following primary knee arthroplasty, and derived separate estimates for costs in the first 12 months after TKR and annual costs between one and 12 years after TKR. Data on nurse visits and visits to the "Accidents & Emergency" department were not available from KAT, which means that ambulatory costs after TKR might be slightly underestimated. For TKR, we also conducted a separate analysis of ambulatory costs in the year of revision surgery and in subsequent years.

Finally, we analysed costs in the year before primary arthroplasty based on data from COASt. We included both ambulatory costs (as above) and hospital admissions.

Costing

We chose 2014 as the reference year for costs, since this is the most recent year which is completely observed in our data extract.

Hospital admissions in the HES data were valued using the NHS Local Payment Grouper 2014/15[25] and prices from the 2014/15 National Schedule.[26] For readmissions, we distinguished between elective and non-elective admissions and used the respective prices. For those hospital readmissions that could not be costed (e.g., due to errors in the grouping process or missing data on length of stay) we imputed costs by using either the mean cost per bed-day for admissions in the same category (if data on length of stay was available), or (if data on length of stay was missing) by using the mean cost per admission that meet the same criteria as a relevant readmission episode. The imputed values are provided in Table B.1 of the supplementary file. We did not impute costs for primary and revision arthroplasty episodes and omitted observations that could not be costed.

Costs for ambulatory visits and hospital admissions in KAT and COASt were derived by attaching the relevant unit costs for 2014 (see supplementary file Table B.1) to the number of visits/appointments.

For peer review only - http://bmjopen.bmj.com/site/about/guidelines.xhtml

Covariates

The OHS and OKS are PROMs, each consisting of 12 questions about pain and functional limitations specific to the joint. The total score varies between 0 and 48, with higher values indicating less pain and functional limitations.[27,28] We used OHS/OKS scores measured before primary arthroplasty in all models.

We also included age and sex in the regression models. When studying the impact of OHS/OKS on costs of primary arthroplasty, quality-of-life, costs before arthroplasty and costs in the first 12 months following arthroplasty, we included age at operation in the regression models. When studying the impact of OHS/OKS on costs more than 12 months after primary arthroplasty, we also considered including current age instead of age at operation as well as a time trend into the model. While the inclusion of further covariates (e.g., body mass index or the American Society of Anesthesiologists (ASA) physical status classification) would be desirable, this information was unfortunately not available in the NHS PROMs and HES datasets.

Descriptive statistics

Table 1 below provides an overview over all outcomes for which regression models were developed. Average post-operative EQ-5D utility was considerably higher than average pre-operative utility. Interestingly, average EQ-5D utility before revision surgery was similar to or even lower than average pre-operative utility. Average utility after revision was higher than average utility values before revision, however, it was lower than the average post-operative utility. On average, primary hip arthroplasty costed £5,522 and primary knee arthroplasty costed £6,053. Average cost in the 12 months before arthroplasty were £444 for THR and £836 for TKR. In the first 12 months after THR, average costs were £101 for ambulatory services and £455 for hospital readmissions. For TKR, average costs in the first 12 months after surgery were £361 for ambulatory services and £550 for hospital readmissions. Average pre-operative OHS scores varied between 16.8 and 18.7 (due to the different samples), while average pre-operative OKS scores varied between 16.9 and 19.4. For the sake of brevity, we discuss the modelling results for selected outcomes in this paper; the models for all other outcomes are provided in the appendix.

For peer review only - http://bmjopen.bmj.com/site/about/guidelines.xhtml

Table 1: Data sources and sample sizes

Variable of interest	Dataset	Mean ± Standard deviation (dependent variable)	Mean OHS	Final sample size	Missing covariates	Table	Figure
**A. Quality-of-life for THR**							
Post-operative EQ-5D utility	PROMs-HES	0.784 ± 0.247	18.1	208,345	0.96%	C.1	1.A
Pre-operative EQ-5D utility	PROMs-HES	0.334 ± 0.324	17.6	271,045	0.83%	C.2	1.A
EQ-5D utility before revision surgery	PROMs-HES	0.348 ± 0.341	17.2	1,391	1.21%	C.4	-
EQ-5D utility after revision surgery	PROMs-HES	0.648 ± 0.316	17.7	884	0.90%	C.5	-
**B. Costs (in £) for THR**							
Costs of primary arthroplasty	PROMs-HES	5,522 ± 744	17.5	286,507	1.12%	C.6	1.C
Costs of revision arthroplasty	PROMs-HES	7,994 ± 2,362	16.9	2,359	1.17%	C.7	1.E
Costs in the 12 months before arthroplasty	COASt	444 ± 679	18.7	441	16.00%	C.8	2.A
Ambulatory costs <12 months after primary arthroplasty	COASt	101 ± 244	18.5	548	16.72%	C.9	2.E
Readmissions <12 months after primary arthroplasty	PROMs-HES	455 ± 2,150	17.5	236,514	1.48%	C.10	2.C
Annual cost of readmissions between 1 and 6 years after primary arthrolasty	PROMs-HES	118 ± 1,137	17.6	476,514	0.98%	C.11	3.A
Readmissions in the year of revision surgery	PROMs-HES	780 ± 3,507	16.8	1,669	1.07%	C.12	3.C
Annual cost of readmissions after revision surgery	PROMs-HES	148 ± 1,309	17.0	2,406	0.87%	C.13	3.E
**C. Quality-of-life for TKR**							
Post-operative EQ-5D utility	PROMs-HES	0.723 ± 0.259	19.0	223,836	0.96%	C.1	1.B
Pre-operative EQ-5D utility	PROMs-HES	0.391 ± 0.317	18.4	290,983	0.98%	C.2	1.B
EQ-5D utility between 1 and 12 years after primary arthroplasty	KAT	0.693 ± 0.276	18.4	15,414	0.00%	C.3	-
EQ-5D utility before revision surgery	PROMs-HES	0.291 ± 0.331	17.0	2,227	1.07%	C.4	-
EQ-5D utility after revision surgery	PROMs-HES	0.544 ± 0.340	17.7	1,398	0.99%	C.5	-
**D. Costs (in £) for TKR**							
Costs of primary arthroplasty	PROMs-HES	6,053 ± 1,097	18.4	308,638	1.21%	C.6	1.D

For peer review only - http://bmjopen.bmj.com/site/about/guidelines.xhtml

Costs of revision arthroplasty	PROMs-HES	7,759 ± 2,777	16.9	3,416	1.13%	C.7	1.F
Costs in the 12 months before arthroplasty	COASt	836 ± 2,131	19.4	278	10.03%	C.8	2.B
Ambulatory costs <12 months after primary arthroplasty	KAT	361 ± 400	18.3	1,841	3.21%	C.9	2.F
Readmissions <12 months after primary arthroplasty	PROMs-HES	550 ± 2,472	18.4	255,194	1.22%	C.10	2.C
Annual cost of readmissions between 1 and 6 years after primary arthrolasty	PROMs-HES	176 ± 1,311	18.4	514,047	1.18%	C.11	3.B
Readmissions in the year of revision surgery	PROMs-HES	844 ± 3,361	16.9	2,258	1.05%	C.12	3.D
Annual cost of readmissions after revision surgery	PROMs-HES	246 ± 1,877	16.9	3,153	1.10%	C.13	3.F
Annual ambulatory costs between 1 and 12 years after primary arthroplasty	KAT	29 ± 104	18.4	13,271	4.13%	C.14	-
Ambulatory costs in the year of revision surgery	KAT	578 ± 718	17.8	88	4.35%	C.15	-
Annual ambulatory costs after revision surgery	KAT	129 ± 235	18.1	329	3.24%	C.16	-

Notes: Tables C.1-C.16 are shown in the online supplementary file. "Missing covariates" shows the fraction of observations that were excluded from the final sample due to missing data on one of the covariates (incl. OHS/OKS) included into the model.
*: Model estimated on longitudinal data.

For peer review only

Statistical methods

The regression models presented in this study focus on predictive accuracy rather than causal inference. To this end, we developed a model selection procedure to systematically identify the best performing model from a range of candidate models. We began by conducting an exploratory data analysis for each outcome of interest to identify a range of candidate models. We visually inspected the distribution of each outcome as well as plots of the outcome against OHS/OKS and age. Based on these figures (available upon request), we identified candidates for *(i)* the statistical model class (e.g., ordinary least squares (OLS) or Tobit regression), *(ii)* the functional form for OHS/OKS (e.g., linear or quadratic trends), *(iii)* the functional form of the age trend, and *(iv)* functional forms for the time trend (where applicable). In principle, any combination of these four components identifies a valid candidate model. However, in practice it was not feasible to test all possible candidate models against each other. Therefore, we selected our final model in four steps:

- In the first step, we selected the most appropriate model class from among the candidate models. All models included a linear trend for OHS/OKS, a linear trend for age and an indicator for sex of the patient and models on longitudinal data also

For peer review only - http://bmjopen.bmj.com/site/about/guidelines.xhtml

included a linear trend for time since arthroplasty. Depending on the exploratory data analysis, we considered linear regression models, generalized linear models (GLM), two-part and Tobit regression models for censored data.

- Where applicable, we then chose the functional form for the time trend using the model class identified in the first step.
- In the second step, we chose the functional form for OHS/OKS. We used the model class identified in the first step and included a linear trend for age as well as an indicator for sex of the patient. We considered polynomials (e.g., linear, quadratic or cubic polynomials) as well as linear splines, where the spline points were identified in the exploratory data analysis.
- The third step consisted of choosing the functional form for age. We used the model class identified in the first step and the functional form for OHS/OKS from the second step. We considered polynomial trends, linear splines as well as age categories, depending on the exploratory data analysis. We also considered whether excluding age would improve prediction accuracy. When modelling costs more than 12 months after primary arthroplasty, we first examined whether age at operation or current age resulted in higher predictive accuracy using linear trends before choosing the functional form for the chosen age variable.
- In the fourth and last step, we evaluated an alternative model selected in the third step that omitted the indicator for sex of the patient, to assess whether this improved prediction accuracy.

We used mean squared error (MSE) as our criterion for model selection and used a 10-fold cross-validation process to avoid overfitting. We randomly divided our sample into 10 slices. For each of the steps outlined above, we estimated the candidate models using nine of these data slices, then predicted the outcomes for the slice left out of the estimation, and finally calculated the mean squared error of the prediction. This was repeated 10 times, so that each data slice was used once for prediction (i.e., internal validation) and the squared error was calculated for each candidate model and for every observation in the sample. Then, we averaged across all patients (in all 10 slices) to estimate the MSE for each candidate model, and chose the model with the lowest MSE. Finally, we re-estimated the model using the whole dataset to obtain our final estimates.

All regression models were estimated using Stata version 14 (StataCorp, College Station TX). We conducted a complete-case analysis, omitting observations with missing data on any of the variables of interest. For the model selection procedure, we omitted all observations with missing data on outcome, OHS or OKS, age as well as sex to ensure that all candidate models were evaluated based on the same dataset. For the final estimation, we only omitted observations with missing data on any of the included variables. With the exception of the models estimated on COASt data, very few observations were excluded due to missing information on covariates (Table 1). We visualise the estimated associations between OHS/OKS and quality-of-life as well as costs by plotting predicted costs and quality-of-life against OHS/OKS scores. We generated predictions using the estimation sample, i.e., for each OHS/OKS score we averaged predictions over the sample distributions of the other covariates. The full estimation results as well as the candidate models considered for each outcome are provided in section C of the supplementary file. Section D of the supplementary file provides details and examples for calculating predictions from the different models used in this study.

For peer review only - http://bmjopen.bmj.com/site/about/guidelines.xhtml

RESULTS

Quality-of-life

Panel A and B in Figure 1 below show the relationship between OHS/OKS and pre-operative as well as post-operative EQ-5D utility. The lines show the fitted values, while the grey areas represent 95% confidence intervals. Both pre-operative and post-operative quality-of-life were higher for patients with high OHS/OKS scores (Figure 1A-B). However, the relationship is clearly non-linear. Pre-operative quality-of-life increased considerably from -0.17/-0.16 for OHS/OKS of 0 to 0.59/0.61 for OHS/OKS scores of 25, beyond which the increase in quality-of-life by OHS/OKS was much smaller. In contrast, post-operative quality-of-life increased strongly from 0.39/0.24 for OHS/OKS of 0 to 0.72/0.61 for scores of 10, after which there were only small differences in quality-of-life by OHS/OKS. As a consequence, the health gain associated with TJR (i.e., the difference between the two curves) was largest for patients with low scores (0.69 for OHS of 5 and 0.53 for OKS of 6). While the models predicted that post-operative EQ-5D utility was higher than pre-operative utility for all patients with OHS ≤46 and those with OKS ≤44, the differences between post- and pre-operative utility were marginal (0.005 for OHS of 46 and 0.02 for OKS of 44).

There was some variation in post-operative EQ-5D by age and sex: post-operative utility was highest for THR patients aged 60 to 69, and increased with age for patients aged 30 to 60 and then decreased with age for patients aged above 70. Post-operative utility for TKR patients increased with age for all patients. Compared to men, women had higher pre-operative utility before TKR ($p<0.001$), but lower post-operative utility after THR ($p<0.001$). Sex was excluded in the models for utility before THR and utility after TKR.

Costs of primary and revision arthroplasty

The cost of primary arthroplasty decreased with OHS/OKS for scores between 0 (£6,132 for THR and £6,603 for TKR on average) and 10 (£5,559 for THR and £6,080 for TKR on average) (Figure 1C and 1D). For scores between 10 and 40, the costs remained approximately constant. For scores above 40, the predicted costs changed only slightly, while the uncertainty around these estimates increased considerably, since less than 0.4% of the patients in the sample had a score above 40. Costs for primary THR were lower (£22 on average) for women than for men ($p<0.001$), while the costs of TKR were higher (£9 on average) for women ($p=0.026$).

Figures 1E and 1F show the relationship between the cost of revision arthroplasty and pre-operative OHS/OKS score. Due to the smaller number of revisions, the uncertainty around these estimates is larger than for the costs of primary surgery, but the estimates suggest that the costs of THR revision surgery increased on average by £15 for a one-point increase in OHS ($p=0.016$), while the costs of TKR revision surgery remained constant ($p=0.837$ on the linear trend). The cost of revision arthroplasty was lower for older patients. Costs for THR revision surgery were on average £429 lower for women than for men ($p<0.001$).

Costs in the year before TJR and in the first 12 months after surgery

Costs in the year before THR decreased significantly with increasing OHS (e.g., £579 at OHS of 5 and £320 at OHS of 32; $p=0.003$), while costs before TKR varied little between OKS values of 0 and 20 (£897 at OKS of 10, £886 at OKS of 20), before decreasing thereafter (£513 at OKS of 32) (Figure 2A and 2B) However, for both THR and TKR the curves are relatively flat. Costs decreased (albeit insignificantly) with age for both THR and TKR. Since

For peer review only - http://bmjopen.bmj.com/site/about/guidelines.xhtml

there was only one patient with an OHS of 33, who was relatively young, the age effect produced an apparent spike in costs for an OHS score of 33.

Figures 2C and 2D show the estimated association between OHS/OKS and ambulatory costs in the first 12 months after TJR. Overall, ambulatory costs after THR were considerably lower than costs after TKR. A sensitivity analysis estimating ambulatory costs for TKR patients in COASt confirmed this finding, suggesting that this trend reflects differences between joints rather than between datasets. Ambulatory costs decreased with increasing OHS/OKS scores from £51/£446 at OHS/OKS scores of 5 and 6, respectively, to £46/£286 at OHS/OKS scores of 32. The negative predicted costs in Figure 2C are an artefact of the simple linear regression model, which does not restrict the outcome to be non-negative. It should be noted that the estimates for Figures 2A-D are based on substantially smaller numbers than previous figures and are subject to considerable uncertainty.

Costs of hospital readmissions

In line with the findings for the costs of primary surgery, the cost of hospital readmissions in the first 12 months after TJR decreased steeply with OHS/OKS for scores between 0 (£1,543 for THR, £1,700 for TKR) and 10 (£545 for THR, £684 for TKR), while for scores above 10 the curve was almost flat (Figure 2E and 2F). Both for THR and TKR the costs were lower for women than for men (on average £78 for THR and £133 for TKR; $p<0.001$ for both models).

Figure 3 shows the relationship between OHS/OKS and the annual costs of hospital readmissions between 1 and 6 years after primary arthroplasty. The costs of hospital readmissions per year for patients without revision surgery are shown in Figures 3A and 3B. For both THR and TKR the costs decreased with OHS/OKS (£161/£266 for OHS/OKS scores of 5 and 6, respectively, £85/£120 for OHS/OKS scores of 32; $p<0.001$). For THR, the costs in year 2 (i.e., between 12 and 24 months after THR) were significantly (on average £32; $p<0.001$) higher than in subsequent years. For TKR, the time trend indicates that readmission costs decreased over time (on average £31 per year; $p<0.001$).

Hospital readmission costs in the year of revision surgery did not have a statistically significant association with OHS/OKS scores measured before primary arthroplasty (Figures 3C and 3D).

Finally, the costs of hospital readmissions per year for patients who had undergone revision surgery >12 months previously (Figures 3E and 3F) were higher for patients who had low OHS/OKS scores before primary arthroplasty ($p<0.05$). However, the difference between patients with very low and very high scores was relatively small.

Table 2 summarizes the predicted quality-of-life and costs for patients with selected OHS/OKS scores averaged over the sample distribution of age and sex. Differences in quality-of-life between patients with very high scores (95th percentile) and very low scores (5th percentile) were very large (0.75/0.72 point difference in pre-operative EQ-5D utility and 0.28/0.35 point difference in post-operative utility for THR/TKR). Differences in costs were moderate in comparison. The costs of primary arthroplasty differed by £245 and £136 for THR and TKR, respectively. Costs in the year before surgery differed by £259 and £450, respectively. The cost of hospital readmissions in the first year differed by £427 and £484 for THR and TKR, while annual costs for hospital readmissions in years 1 to 6 after primary arthroplasty differed by £76 and £146 for THR and TKR patients.

For peer review only - http://bmjopen.bmj.com/site/about/guidelines.xhtml

Table 3 shows predicted average quality-of-life and costs for patients in different age groups. Associations between quality-of-life and age were weak overall, the largest difference between patients aged 80 and above and patients below age 60 was predicted for utility after revision TKR revision surgery (0.1 point difference). Differences in costs were more pronounced, with differences in primary arthroplasty costs of £343 and £316 for THR and TKR. However, in contrast to Table 2 the differences were not systematic. For example, costs of primary arthroplasty, readmission costs in the first 12 months and readmission costs in the year of revision surgery were higher for older patients, while other costs (e.g., ambulatory costs in the first 12 months) were lower for older patients.

For peer review only

Table 2: Predicted quality-of-life and costs by pre-operative OHS/OKS at primary operation

Outcome	THR			TKR		
	OHS 5 (5th pct)	OHS 17 (50th pct)	OHS 32 (95th pct)	OKS 6 (5th pct)	OKS 18 (50th pct)	OKS 32 (95th pct)
**A. Quality-of-life**						
Post-operative EQ-5D utility	0.612	0.798	0.890	0.498	0.735	0.851
Pre-operative EQ-5D utility	-0.082	0.354	0.671	-0.033	0.421	0.687
EQ-5D utility > 12 months after primary arthroplasty	-	-	-	0.501	0.700	0.813
EQ-5D utility before revision surgery	0.120	0.363	0.544	0.054	0.355	0.474
EQ-5D utility after revision surgery	0.437	0.673	0.783	0.293	0.572	0.684
**B. Costs (in £)**						
Costs of primary arthroplasty	5,687	5,502	5,442	6,158	6,036	6,021
Costs of revision arthroplasty	7,768	7,957	8,160	7,804	7,751	7,678
Costs in the 12 months before arthroplasty	579	416	320	963	898	513
Readmissions <12 months after primary arthroplasty	740	422	314	888	499	404
Ambulatory costs <12 months after primary arthroplasty	52	131	47	446	352	286
Readmissions >12 months after primary arthrolasty	161	115	85	266	169	120
Readmissions in the year of revision surgery	675	747	621	959	860	758
Readmissions > 12 months after revision surgery	238	144	73	360	225	150
Ambulatory costs >12 months after primary arthroplasty	-	-	-	48	27	19
Ambulatory costs in the year of revision surgery	-	-	-	816	533	351
Ambulatory costs for patients after revision surgery	-	-	-	128	155	36

Notes: The columns show average predicted quality-of-life and costs for patients with different pre-operative OHS and OKS scores. Predictions are averaged across the estimation sample. Column 1 and 4 show values for patients at the 5th percentile of the OHS/OKS distribution. Columns 2 and 4 show outcomes for patients at the median of OHS/OKS distributions, and columns 3 and 6 show outcomes for patients at the 95th percentile of the OHS/OKS distribution.

For peer review only - http://bmjopen.bmj.com/site/about/guidelines.xhtml

Table 3: Predicted quality-of-life and costs by age at operation

	THR			TKR		
**Outcome**	**Age <60**	**Age 60-79**	**Age ≥80**	**Age <60**	**Age 60-79**	**Age ≥80**
**A. Quality-of-life**						
Post-operative EQ-5D utility	0.778	0.794	0.729	0.637	0.731	0.722
Pre-operative EQ-5D utility	0.316	0.348	0.281	0.310	0.404	0.397
EQ-5D utility > 12 months after primary arthroplasty	-	-	-	0.616	0.689	0.645
EQ-5D utility before revision surgery	0.301	0.356	0.386	0.225	0.329	0.312
EQ-5D utility after revision surgery	0.572	0.670	0.562	0.418	0.558	0.521
**B. Costs (in £)**						
Costs of primary arthroplasty	5,420	5,504	5,763	5,939	6,039	6,255
Costs of revision arthroplasty	8,108	7,938	7,991	8,020	7,685	7,390
Costs in the 12 months before arthroplasty	626	394	398	1,150	804	634
Readmissions <12 months after primary arthroplasty	370	430	726	504	519	762
Ambulatory costs <12 months after primary arthroplasty	151	96	62	561	353	243
Readmissions >12 months after primary arthrolasty	135	117	110	218	171	156
Readmissions in the year of revision surgery	633	761	1,344	763	857	941
Readmissions > 12 months after revision surgery	159	151	139	428	242	105
Ambulatory costs >12 months after primary arthroplasty	-	-	-	50	27	27
Ambulatory costs in the year of revision surgery	-	-	-	711	556	160
Ambulatory costs for patients after revision surgery	-	-	-	145	124	118

Notes: The columns show average predicted quality-of-life and costs for patients in different age groups at primary operation. Predictions are averaged across the estimation sample.

For peer review only

DISCUSSION

OHS and OKS were developed as outcome measures reported by patients undergoing THR and TKR. Previous studies found that OHS and OKS are correlated with both clinical scores (such as the Charnley score or the American Knee Society score) and patient-reported general health measures (such as SF-36).[27,28] Thus, it is not surprising that we found that higher scores were systematically associated with higher quality-of-life. However, the difference between post-operative and pre-operative EQ-5D utility decreased with OHS/OKS. Patients with low scores have worse symptoms and more functional limitations due to osteoarthritis. Therefore, they have more potential to benefit from primary arthroplasty.

For peer review only - http://bmjopen.bmj.com/site/about/guidelines.xhtml

We also found that costs are moderately higher for patients with low pre-operative scores. Patients with worse symptoms due to osteoarthritis might require longer recovery times. In addition, they are likely to have comorbidities or complications, which also increase the costs of primary arthroplasty. While the differences in costs are moderate compared to differences in quality-of-life (between £100 and £500 for most outcomes), it is worth noting that some of these costs are incurred annually, and therefore even moderate differences can be economically important in the long-run.

Our findings do not allow us to draw conclusions about the cost-effectiveness of TJR for patients with different pre-operative scores without further assumptions about the costs and quality-of-life for patients without TJR. Nevertheless, based on these findings it appears promising to investigate differences in cost-effectiveness as well as the impact of rationing policies. In particular, the finding that the associations between pre-operative score and costs as well as quality-of-life are nonlinear suggests that there could be a threshold score beyond which TJR might not be considered beneficial or cost-effective. However, identifying this threshold is beyond the scope of this paper and left to future research.

Two previous studies on the cost-effectiveness of TKR and THR, respectively, found that while OHS/OKS were systematically associated with both costs and outcomes of TJR, both procedures were nonetheless cost-effective for almost all patients.[3,4] Similarly, our results indicate that quality-of-life improved for almost all patients undergoing TKR and THR. While the improvement was largest for patients with low scores, we also found that costs tended to be higher for patients with low scores.

A recent study concluded that, in the US, access to TKR could be restricted to patients with more severe symptoms.[14] Similar to our findings, they reported that changes in quality-of-life were lower for patients with fewer pre-operative symptoms (measured by SF-12 physical score). However, in their study TKR had minimal effects on quality-of-life on average (SF-6D utility increased by 0.008). Our findings contrast strongly with this, indicating that at least in the UK, TJR is associated with sizable increases in EQ-5D-3L utility (average change of 0.32 for TKR and 0.45 for THR) for almost all patients currently undergoing surgery. This discrepancy may be due to differences between EQ-5D-3L and SF-6D, differences between our sample covering almost all UK knee replacement procedures and a small US sample of early osteoarthritis patients, or methodological differences between the regression models selected for predictive accuracy in this study and marginal structural models used in the previous study.

Since OHS and OKS are already being used to determine eligibility for TJR,[6] evidence on their impact on the cost, efficacy and cost-effectiveness of primary arthroplasty is required to inform guidelines. The results presented in this study provide evidence on the association between pre-operative scores and important measures of costs and quality-of-life. Moreover, the models presented here can be used as inputs in future model-based studies assessing the cost, efficacy and/or cost-effectiveness of THR and TKR, and in particular to explore heterogeneity between patient subgroups.

In this study, we used data from a range of different sources, including clinical trials and comprehensive administrative databases. The sample sizes in this study are up to 1000 times larger than those used in previous studies.[3,4,14] Crucially, this reduces the uncertainty around our estimates for patients with very high scores – i.e., patients who are most likely to be affected by rationing. Our data also had a longer follow-up duration for TKR than previous studies (up to 12 years compared with five years in previous studies[4,14]).

For peer review only - http://bmjopen.bmj.com/site/about/guidelines.xhtml

Moreover, we employed a systematic model selection procedure to select the most appropriate statistical model from a range of different candidate models.

Nevertheless, there are several limitations to our study. KAT and COASt are markedly smaller than NHS PROMs-HES, and consequently models estimated on those datasets have more uncertainty and are relatively parsimonious. On the other hand, these datasets allowed us to estimate models for a larger range of relevant outcomes. While NHS PROMs-HES data include information on (nearly) all elective TJR procedures carried out in the NHS England, it nevertheless is a selected sample. In particular, pre-operative scores are already being used in some regions to restrict access to TJR, which affects the distribution of pre-operative scores in the data. Moreover, the findings of this study might not hold for patients that are currently denied access to TJR if those patients differ systematically from those observed in the data. A report commissioned by the Royal College of Surgeons in 2014 noted that 16 out of 52 Clinical Commissioning Groups (CCG) restricted access to THR based on an OHS threshold score.[6] However, current guidelines do not recommend a threshold for referral, and thus existing threshold scores are not based on evidence and they are not applied systematically. Therefore, these existing approaches should not have a major impact on our findings. We were not able to draw conclusions on total costs or cost-effectiveness based on the findings of this study. This requires long-term modelling work, for which the models presented in this study could serve as parameters. The costs derived in this study could be affected by coding and classification errors. For example, we identified primary arthroplasty episodes in NHS PROMs-HES data based on links between NHS PROMs questionnaires and HES episodes instead of narrowly defined procedure and diagnosis codes. We were also not able to distinguish between one-stage and two-stage revisions. Consequently, our estimates of the costs could be affected by miscoded diagnoses, procedures or data linkage. We used specific criteria to determine whether hospital readmissions were related to primary arthroplasty. Similarly, in COASt and KAT patients were asked to report health services used for problems related to their joint and their primary arthroplasty operation. Therefore, ambulatory and hospital readmission costs might be affected by classification errors. While coding and classification errors might bias the costs reported in this study, they are unlikely to affect the estimated associations between OHS/OKS and costs, since it does not seem plausible that the frequency of such errors is related to patient's pre-operative score. The costs were derived from the National Tariff as well as published reference costs. In some cases these costs might not reflect the true costs to the hospital, although there is no reason to suspect that they would differ systematically.

We conducted a complete-case analysis, since the use of multiple imputation would have further increased the complexity of our analysis. However, there is little reason to expect that missing data would be systematically related to the shape of the relationship between OHS/OKS and costs or quality-of-life. While we studied a wide range of outcome variables, we were not able to examine revision rates or long-term outcomes for THR due to a lack of appropriate data. We also focused on a limited range of covariates (OHS/OKS, age and sex). We did not investigate the impact of other covariates that may affect our outcomes and are used in clinical decision-making (e.g., body mass index) due to a lack of data. While we tested a wide range of candidate models, our model selection was done sequentially, and therefore it is possible that there are interactions between model components that were not considered in this study. Our model selection process was developed to assess prediction accuracy, and therefore we did not consider statistical significance or confounding when selecting our models. Finally, our model selection process did not take into account whether differences in model fit were statistically significant and it did not penalize model complexity.

For peer review only - http://bmjopen.bmj.com/site/about/guidelines.xhtml

These limitations affect our findings to varying degrees: There is very little uncertainty around the associations between OHS/OKS and quality-of-life as well as the cost of primary arthroplasty, since the underlying models were estimated on large samples. Similarly, our findings for the cost of hospital readmissions were based on large samples, however, the cost variables are more likely to be affected by the coding and classification errors mentioned above. While our findings on ambulatory costs and cost after revision arthroplasty are based on the best available datasets, there is still considerable uncertainty around these estimates due to relatively small sample sizes as well as coding and classification errors. These shortcomings could be addressed in future research, e.g., through the use of administrative datasets on primary care such as the Clinical Practice Research Datalink (CPRD) as well as data on revision arthroplasty from the National Joint Registry (NJR). Unfortunately, these datasets were not available for this research.

In summary, our results show that pre-operative OHS and OKS are systematically associated with the costs and quality-of-life of patients undergoing TJR. However, further research is needed to estimate cost-effectiveness, and to determine the impact on revision rates and long-term outcomes for THR.

Acknowledgements

We would like to thank the ACHE investigators, user group and steering group for their role in the project.

We thank the KAT study group for providing access to their data for use in this project, as well as all of the researchers and participants involved in that study. We would like to thank all the participants of the COASt Study, Professor Nigel Arden and the COASt team for their time and dedication, and the NIHR for their funding support.

KAT was funded by the NIHR as an HTA (No. 95/10/01). COASt was funded by the National Institute for Health Research (NIHR) under its Programme Grants for Applied Research Programme (Reference Number RP-PG-0407-10064).

Data on NHS PROMs linked to HES APC data were reused with the permission of NHS Digital, copyright 2015, with all rights reserved.

The views and opinions expressed in this paper are those of the authors and do not necessarily reflect those of the HTA programme, NIHR, NHS, NHS Digital or the Department of Health.

We would also like to thank Chaudhry Shah and Joaquim Soares do Brito for their help with identifying the relevant codes for hospital readmissions used in the HES data.

Funding

This study was conducted as part of the project "Introducing Standardised And Evidence-Based Thresholds for Hip and Knee Replacement Surgery – The Arthroplasty Candidacy Help Engine (The ACHE Tool)", which was funded under the UK National Institute of Health Research (NIHR) Health Technologies Assessment (HTA) Programme (HTA 11/63/01). Visit the HTA programme website (www.hta.ac.uk) for further project information. The funder had no involvement in: the collection, analysis, and interpretation of

For peer review only - http://bmjopen.bmj.com/site/about/guidelines.xhtml

data; in the writing of the report; and in the decision to submit the article for publication. The authors had full access to the data (including statistical reports and tables), can take responsibility for the integrity of the data and the accuracy of the data analysis and are responsible for submitting this paper.

Competing interest statement

All authors have completed the Unified Competing Interest form at www.icmje.org/coi_disclosure.pdf (available on request from the corresponding author). All authors received a grant from NIHR funding the current work. HD reports personal fees from Halyard Health, outside the submitted work. NA reports grants from Bioiberica, personal fees from Bioventus, personal fees from ESCEO, personal fees from Flexion, personal fees from Freshfields Bruckhaus Deringer, personal fees from Merck, personal fees from Regeneron, outside the submitted work. The other authors declare that there are no relevant conflicts of interests.

Ethical Approval

No primary data was collected for this study, and therefore ethical approval was not required. The COASt study obtained ethical approval from Oxford research Ethics Committee (REC Ref: 10/H0604/91) and written consent was obtained from each participant. The KAT trial was approved by the Multi Centre Research Ethics Committee for Scotland in November 1998 (research protocol MREC/98/0/100) and was approved by the Local Research Ethics Committees in each study centre recruiting trial participants.

Copyright/licence

The Corresponding Author has the right to grant on behalf of all authors and does grant on behalf of all authors, a worldwide licence to the Publishers and its licensees in perpetuity, in all forms, formats and media (whether known now or created in the future), to i) publish, reproduce, distribute, display and store the Contribution, ii) translate the Contribution into other languages, create adaptations, reprints, include within collections and create summaries, extracts and/or, abstracts of the Contribution, iii) create any other derivative work(s) based on the Contribution, iv) to exploit all subsidiary rights in the Contribution, v) the inclusion of electronic links from the Contribution to third party material where-ever it may be located; and, vi) licence any third party to do any or all of the above.

Authorship statement

AP, DB, HD, AG and NA conceived, designed and conducted the ACHE study. PE, HD and AG designed and conducted the regression analysis reported in this manuscript. PE and HD analysed the data. PE drafted the manuscript. All authors edited the manuscript for important intellectual content and approved the final version.

Transparency declaration

The manuscript's guarantor (PE) affirms that the manuscript is an honest, accurate, and transparent account of the study being reported; that no important aspects of the study have been omitted; and that any discrepancies from the study as planned (and, if relevant, registered) have been explained.

Data sharing

For peer review only - http://bmjopen.bmj.com/site/about/guidelines.xhtml

The statistical code as well as variance-covariance matrices for the analyses reported in this study are available from the authors upon request. PROMs/HES data are available from NHS Digital (http://content.digital.nhs.uk/dars); enquiries about access to the KAT data should be directed to kat@abdn.ac.uk; enquiries about access to the COASt data should be directed to coast.study@ndorms.ox.ac.uk.

For peer review only

References

1 Gandhi R, Dhotar H, Davey JR, *et al.* Predicting the Longer-term Outcomes of Total Hip Replacement. *J Rheumatol* 2010;**37**:2573–7. doi:10.3899/jrheum.100149

2 Judge A, Batra RN, Thomas GE, *et al.* Body mass index is not a clinically meaningful predictor of patient reported outcomes of primary hip replacement surgery: prospective cohort study. *Osteoarthritis Cartilage* 2014;**22**:431–9. doi:10.1016/j.joca.2013.12.018

3 Fordham R, Skinner J, Wang X, *et al.* The economic benefit of hip replacement: a 5-year follow-up of costs and outcomes in the Exeter Primary Outcomes Study. *BMJ Open* 2012;**2**:e000752. doi:10.1136/bmjopen-2011-000752

4 Dakin H, Gray A, Fitzpatrick R, *et al.* Rationing of total knee replacement: a cost-effectiveness analysis on a large trial data set. *BMJ Open* 2012;**2**:e000332. doi:10.1136/bmjopen-2011-000332

5 Gandhi R, Dhotar H, Razak F, *et al.* Predicting the longer term outcomes of total knee arthroplasty. *The Knee* 2010;**17**:15–8. doi:10.1016/j.knee.2009.06.003

6 The Royal College of Surgeons of England. Is access to surgery a postcode lottery? Published Online First: 2014.https://www.rcseng.ac.uk/-/media/files/rcs/news-and-events/media-centre/is-access-to-surgery-a-postcode-lottery.pdf?la=en (accessed 8 Nov 2016).

7 Coastal West Sussex Clinical Commissioning Group. Low Priority Procedures (LPPs) and Procedures with Restrictions (OPRs), Commissioning for Clinical Effectiveness, NICE Interventional Procedures, version 2.10. Published Online First: 2015.https://www.coastalwestsussexccg.nhs.uk/domains/coastal-west-sussex-ccg.org.uk/local/media/documents/misc/Version_2.10_Low_Priority_Procedures_and_Other_Procedures_with_Restrictions_2015.pdf (accessed 8 Nov 2016).

8 Harrogate and Rural District Clinical Commissioning Group. Clinical thresholds: Hip and knee arthroplasty for osteoarthritis (only). Published Online First: 2014.http://www.harrogateandruraldistrictccg.nhs.uk/data/uploads/rss2/hip-and-knee-arthroplasty.pdf (accessed 8 Nov 2016).

9 Scarborough and Ryedale Clinical Commissioning Group. Hip replacement pathway. Published Online First: 2015.http://www.scarboroughryedaleccg.nhs.uk/data/uploads/rss2/orthopaedics/hip-replacement-march-2015.pdf (accessed 8 Nov 2016).

10 Worcestershire NHS. Commissioning Policy for Musculoskeletal Surgical Interventions. Published Online First: 2010.www.worcestershire.nhs.uk/EasySiteWeb/GatewayLink.aspx?alId=5553 (accessed 8 Nov 2016).

11 Losina E, Walensky RP, Kessler CL, *et al.* Cost-effectiveness of Total Knee Arthroplasty in the United States. *Arch Intern Med* 2009;**169**:1113–22. doi:10.1001/archinternmed.2009.136

For peer review only - http://bmjopen.bmj.com/site/about/guidelines.xhtml

12 Rissanen P, Aro S, Sintonen H, *et al.* Costs and cost-effectiveness in hip and knee replacements. A prospective study. *Int J Technol Assess Health Care* 1997;**13**:575–88.

13 Cushnaghan J, Bennett J, Reading I, *et al.* Long-term outcome following total knee arthroplasty: a controlled longitudinal study. *Ann Rheum Dis* 2009;**68**:642–7. doi:10.1136/ard.2008.093229

14 Ferket BS, Feldman Z, Zhou J, *et al.* Impact of total knee replacement practice: cost effectiveness analysis of data from the Osteoarthritis Initiative. *BMJ* 2017;:j1131. doi:10.1136/bmj.j1131

15 Williams DP, Price AJ, Beard DJ, *et al.* The effects of age on patient-reported outcome measures in total knee replacements. *Bone Jt J* 2013;**95–B**:38–44. doi:10.1302/0301-620X.95B1.28061

16 Judge A, Arden NK, Batra RN, *et al.* The association of patient characteristics and surgical variables on symptoms of pain and function over 5 years following primary hip-replacement surgery: a prospective cohort study. *BMJ Open* 2013;**3**:e002453. doi:10.1136/bmjopen-2012-002453

17 Murray DW, MacLennan GS, Breeman S, *et al.* A randomised controlled trial of the clinical effectiveness and cost-effectiveness of different knee prostheses: the Knee Arthroplasty Trial (KAT). *Health Technol Assess Winch Engl* 2014;**18**:1–235, vii–viii. doi:10.3310/hta18190

18 Arden N, Altman D, Beard D, *et al.* Lower limb arthroplasty: can we predict outcome and failure and is it cost effective? *Health Technol Assess* 2016;**(in press)**.

19 Herbert A, Wijlaars L, Zylbersztejn A, *et al.* Data Resource Profile: Hospital Episode Statistics Admitted Patient Care (HES APC). *Int J Epidemiol* doi:10.1093/ije/dyx015

20 Judge A, Cooper C, Williams S, *et al.* Patient-reported outcomes one year after primary hip replacement in a European Collaborative Cohort. *Arthritis Care Res* 2010;**62**:480–8. doi:10.1002/acr.20038

21 Marques EM, Blom AW, Lenguerrand E, *et al.* Local anaesthetic wound infiltration in addition to standard anaesthetic regimen in total hip and knee replacement: long-term cost-effectiveness analyses alongside the APEX randomised controlled trials. *BMC Med* 2015;**13**:151. doi:10.1186/s12916-015-0389-1

22 Wylde V, Blom AW, Bolink S, *et al.* Assessing function in patients undergoing joint replacement: a study protocol for a cohort study. *BMC Musculoskelet Disord* 2012;**13**:220. doi:10.1186/1471-2474-13-220

23 Feng Y, Parkin D, Devlin NJ. Assessing the performance of the EQ-VAS in the NHS PROMs programme. *Qual Life Res* 2014;**23**:977–89. doi:10.1007/s11136-013-0537-z

24 Dolan P. Modeling Valuations for EuroQol Health States. *Med Care* 1997;**35**:1095–108.

25 The National Casemix Office. HRG4 Grouper Reference Manual Payment 14/15. content.digital.nhs.uk/article/3938/HRG4-201415-Payment-Grouper?tabid=1 (accessed 10 Jun 2015).

For peer review only - http://bmjopen.bmj.com/site/about/guidelines.xhtml

26 Department of Health. Payment by Results in the NHS: tariff for 2014 to 2015: 2014- 15 tariff information spreadsheet. Published Online First: 2014.https://www.gov.uk/government/uploads/system/uploads/attachment_data/file/300551/Annex _5A_National_Prices.xlsx (accessed 19 Oct 2015).

27 Dawson J, Fitzpatrick R, Carr A, *et al.* Questionnaire on the Perceptions of Patients About Total Hip Replacement. *J Bone Jt Surg Br* 1996;**78–B**:185–90.

28 Dawson J, Fitzpatrick R, Murray D, *et al.* Questionnaire on the perceptions of patients about total knee replacement. *J Bone Jt Surg Br* 1998;**80–B**:63–9.

For peer review only

Figure legends

Figure 1

Sources: NHS PROMs and HES data, 2009-2015; own calculations. Costs are measured in 2014 Pound Sterling (£). Lines show predicted values averaged over all observations in the sample with a given OHS/OKS. All models included pre-operative OHS or OKS score, age at operation and sex of the patient as independent variables, with the exception of the model for pre-operative EQ-5D of THR patients, which did not include an indicator for female patients. Full regression results are shown in appendix C. The grey areas show 95% confidence intervals obtained from 1,000 bootstrap replications.

Figure 2

Sources: NHS PROMs, HES, KAT and COASt data, 2009-2015; own calculations. Costs are measured in 2014 Pound Sterling (£). Lines show predicted values averaged over all observations in the sample with a given OHS/OKS. All models included pre-operative OHS or OKS score and age at operation as independent variables. The models for readmission costs also included a binary indicator for female patients. Full regression results are shown in appendix C. The grey areas show 95% confidence intervals obtained from 1,000 bootstrap replications.

Figure 3

Sources: NHS PROMs and HES data, 2009-2015; own calculations. Costs are measured in 2014 Pound Sterling (£). Lines show predicted values averaged over all observations in the sample with a given OHS/OKS. All models included pre-operative OHS or OKS score as independent variables. The model for annual readmission costs between 1 and 6 years after THR additionally included current age and an indicator for time since primary surgery. The corresponding model for TKR included age at operation, time since primary surgery and an indicator for sex of the patient as independent variables. The models in panel B additionally included age at operation, time since primary surgery and sex of the patient as covariates. The model for annual readmission costs after revision TKR surgery included current age and time since primary surgery as additional covariates. Full regression results are shown in appendix C. The grey areas show 95% confidence intervals obtained from 1,000 bootstrap replications.

For peer review only - http://bmjopen.bmj.com/site/about/guidelines.xhtml

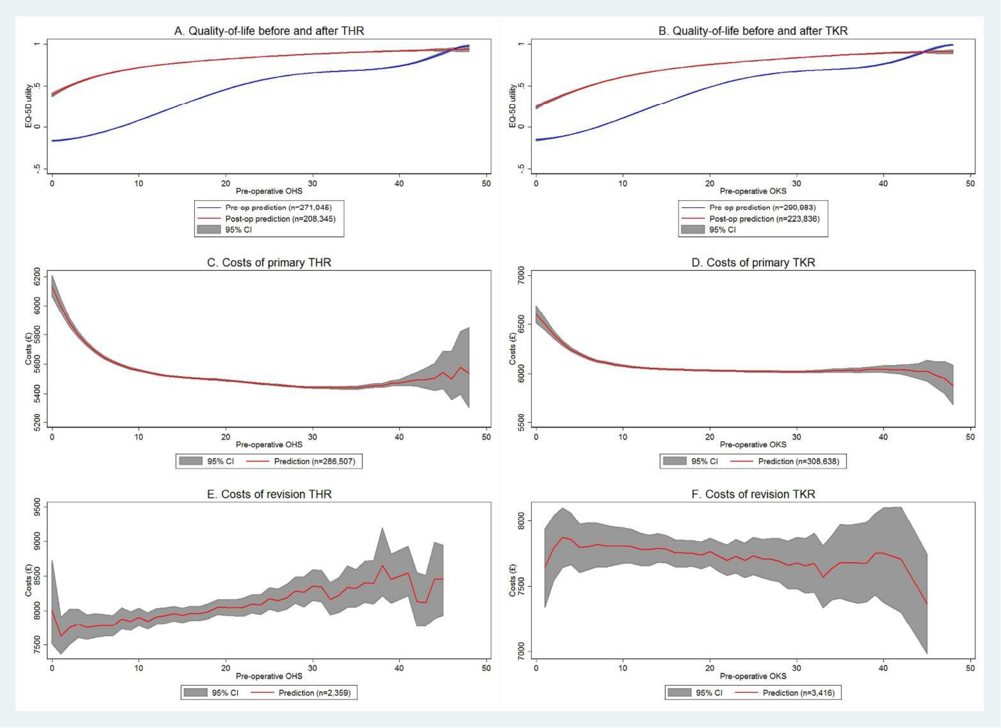

203x147mm (300 x 300 DPI)

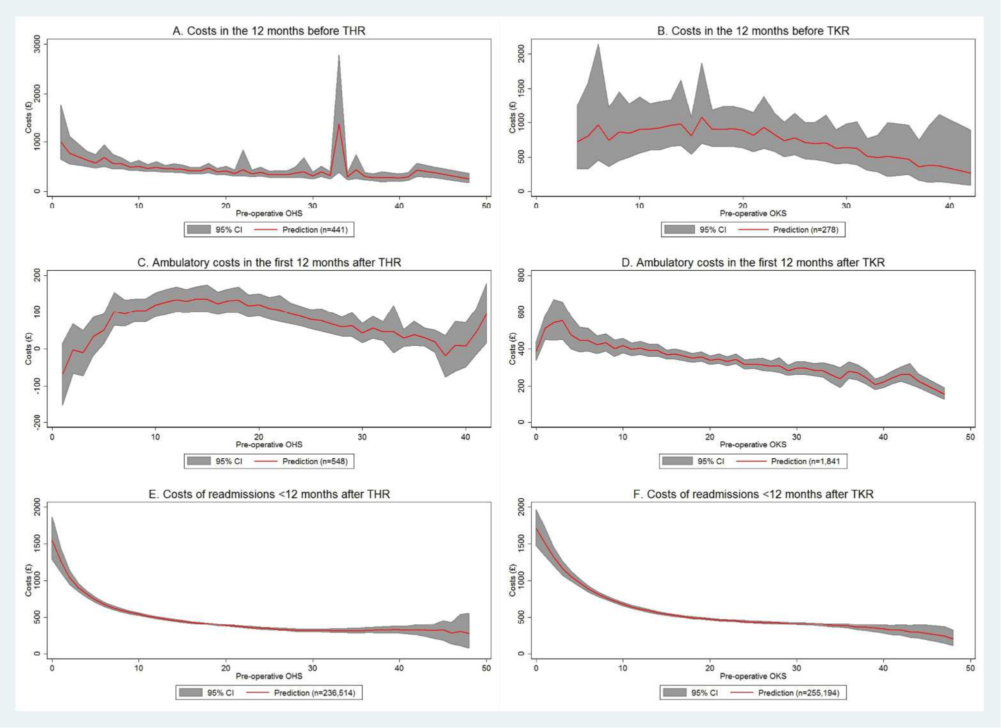

203x147mm (300 x 300 DPI)

ew only

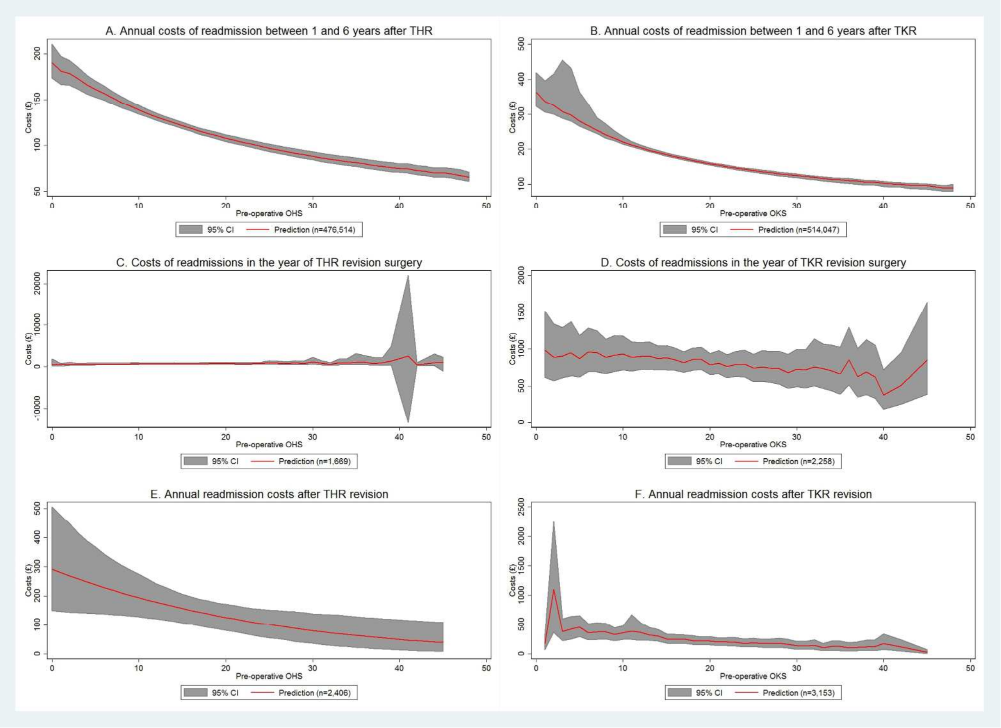

203x147mm (300 x 300 DPI)

ew only

Appendix

A. Diagnoses and procedures codes used to define hospital readmissions

We used ICD-10 diagnosis codes and OPCS 4 procedure codes to determine whether a hospital admission was related to arthroplasty. Specifically, we used the following criteria:

- A hospital admission occurred within 30 days after the primary arthroplasty episode. This criterion was applied regardless of any specific diagnosis or procedure codes.
- The primary diagnosis (i.e., the first recorded ICD-10 diagnosis code) was M17.X (knee arthritis) for a knee replacement episode, or M16.X (hip arthritis) for a hip replacement episode.
- One of the recorded procedure codes referred to the hip or knee joint. The OPCS 4 codes Z843, Z761 or Z756 were used for hip replacement episodes, and the codes Z846, Z765, Z845. Z844, Z774 or Z787 were used for knee replacement episodes.
- The admission had a diagnosis code associated with infections of the skin, the joint or the prosthesis. We used the following ICD-10 codes for this purpose:
    - M25.X with Z96.6 – "Other joint disorder, not elsewhere classified" and "Presence of orthopedic joint implant"
    - T81.X with either Z96.6 or Y83.1 – "Complications of procedures, not elsewhere classified" and "Presence of orthopedic joint implant" or "Surgical operation with implant of artificial internal device […]"
    - T84.X – "Complications of internal orthopedic prosthetic devices, implants and grafts"
    - M96.X – "Intraoperative and postprocedural complications and disorders of musculoskeletal system, not elsewhere classified"
    - M00.X with Z96.6 – "Pyogenic arthritis" and "Presence of orthopedic joint implant"
    - M86.X with Z96.6 – "Osteomyelitis" and "Presence of orthopedic joint implant".

For peer review only

B. Unit costs

Table B.1 Unit costs applied to all primary datasets providing resource use data

Healthcare resource	Units	Unit cost	Reference and details
GP surgery visit	Per visit	£46	PSSRU 2014 page 195[1]: Cost of a surgery patient contact lasting 11.7 minutes was £46 including direct care staff costs and qualifications. A clinic patient contact lasting 17.2 minutes costs £67 including direct care staff costs and qualifications.
GP Practice nurse visit	Per visit	£13.69	PSSRU 2014 page 192: £53 per hour of face-to-face contact including qualifications (£44/hour excluding qualifications). Average surgery consultation for a GP practice nurse lasts 15.5 minutes, giving a consultation cost of £14
Community physiotherapist at clinic/GP surgery	Per visit	£51	NHS reference costs – PSSRU 2014 page 179 states that the mean cost for a one-to-one contact in physiotherapy services in 2013/2014 was £51. PSSRU estimates the unit cost per hour in 2013/2014 to be £32 without qualifications or £36 with qualifications (page 179)
Community physiotherapist – at home	Per visit	£51	For simplicity, applied the same cost as physiotherapy at GP surgery/clinic
Hospital physiotherapist	Per visit	£34	NHS reference costs – PSSRU 2014 page 235 states that the mean average cost for a non-consultant led (not admitted) follow-up physiotherapy attendance in 2013/14 was £34 (IQR £28-£38). PSSRU 2014 estimates the hourly cost of a hospital physiotherapist to be £37/hour including qualifications and £33 excluding qualifications
Physiotherapy – average	Per visit	£42.50	We assumed that 50% of physiotherapy visits are in hospital and 0% are in the community.

For peer review only

Healthcare resource	Units	Unit cost	Reference and details
Outpatient visit – orthopaedics – Follow-up visit	Per visit	£71	Payment by results tariff [2] Tariff information spreadsheet 2013-14: outpatient attendances- WF01A Follow Up Attendance - Single Professional Or WF02A Follow Up Attendance - Multi-Professional (both of these codes are the same price). Follow-up attendance is used here, since patients are assumed to have already attended clinic for their joint replacement
A&E attendance – not admitted	Per visit	£81.26	National schedule of reference costs [3] weighted average of all non-admitted accident and emergency attendances , weighted by the number of attendances.
Admission to hospital related to hip arthroplasty (No data on length of stay)	Per admission	£2,662	Department of Health reference costs 2013-14. [3] This was calculated by taking a weighted average cost of all HRGs with "hip" within the currency description on the EL & NEL worksheets, weighting by the number of FCEs.
Admission to hospital related to knee arthroplasty (No data on length of stay)	Per admission	£3,907	Department of Health reference costs 2013-14 [3]. This was calculated by taking a weighted average cost of all HRGs with "knee" within the currency description on the EL & NEL worksheets, weighting by the number of FCEs.
Readmission to hospital within 30 days of hip or knee arthroplasty	Per bed-day	£395.83	Average costs per bed-day for readmissions in PROMs/HES within 30 days of hip or knee arthroplasty
Readmission to hospital within 30 days of hip or knee arthroplasty (No data on length of stay)	Per admission	£1,255	Average costs for readmissions in PROMs/HES within 30 days of hip or knee arthroplasty
Readmission to hospital with a diagnosis of hip/knee arthritis after hip/knee arthroplasty	Per bed-day	£1,107.25	Average costs per bed-day for readmissions in PROMs/HES with a diagnosis of hip/knee arthritis after hip/knee arthroplasty
Readmission to hospital with a diagnosis of	Per	£5,220	Average costs for readmissions in PROMs/HES with a diagnosis of

For peer review only

Healthcare resource	Units	Unit cost	Reference and details
hip/knee arthritis after hip/knee arthroplasty (No data on length of stay)	admission		hip/knee arthritis after hip/knee arthroplasty
Readmission to hospital for a hip/knee-specific procedure following hip/knee arthroplasty	Per bed-day	£551.33	Average costs per bed-day for readmissions in PROMs/HES for a hip/knee-specific procedure following hip/knee arthroplasty
Readmission to hospital for a hip/knee-specific procedure following hip/knee arthroplasty (No data on length of stay)	Per admission	£1,654	Average costs for readmissions in PROMs/HES for a hip/knee-specific procedure following hip/knee arthroplasty
Readmission to hospital with a diagnosis for infection following hip/knee arthroplasty	Per bed-day	£694.8	Average costs per bed-day for readmissions in PROMs/HES with a diagnosis for infection following hip/knee arthroplasty
Readmission to hospital with a diagnosis for infection following hip/knee arthroplasty (No data on length of stay)	Per admission	£1,876	Average costs for readmissions in PROMs/HES diagnosis for infection following hip/knee arthroplasty

References

[1] PSSRU | Unit Costs of Health and Social Care 2014 n.d. http://www.pssru.ac.uk/project-pages/unit-costs/2014/ (accessed June 14, 2017).
[2] Department of Health. Payment by Results in the NHS: tariff for 2014 to 2015: 2014- 15 tariff information spreadsheet. 2014.
[3] Department of Health. NHS reference costs 2013 to 2014 2014. https://www.gov.uk/government/publications/nhs-reference-costs-2013-to-2014 (accessed June 14, 2017).

For peer review only

C. Regression results

Table C.1: Associations between pre-operative OHS/OKS and post-operative EQ-5D-3L

	**Hip arthroplasty**			**Knee arthroplasty**		
Model	*Tobit model censored at -0.594 and 1*			*Tobit model censored at -0.594 and 1*		
**Variable**	**Coefficient**		**Robust S.E.**	**Coefficient**		**Robust S.E.**
OHS/OKS						
Linear term	5.839E-02	***	4.337E-03	5.725E-02	***	2.482E-03
Quadratic term	-3.312E-03	***	5.400E-04	-2.356E-03	***	2.008E-04
Cubic term	1.182E-04	***	2.990E-05	5.390E-05	***	6.600E-06
Quartic term	-2.110E-06	**	7.520E-07	-4.660E-07	***	7.530E-08
Quintic term	1.460E-08	*	6.980E-09	-	-	-
Age (reference category: 50-54)						
Age <30	-9.039E-02	***	1.717E-02	-1.467E-01	*	6.181E-02
Age 30-39	-4.423E-02	***	1.047E-02	-4.303E-02		2.371E-02
Age 40-49	-1.240E-02	*	6.128E-03	-2.316E-02	**	6.787E-03
Age 55-59	3.204E-03		5.012E-03	1.138E-02	*	4.470E-03
Age 60-64	2.120E-02	***	4.592E-03	4.654E-02	***	4.094E-03
Age 65-69	3.235E-02	***	4.422E-03	8.938E-02	***	3.980E-03
Age 70-74	3.831E-03		4.377E-03	8.991E-02	***	3.966E-03
Age 75-79	-2.073E-02	***	4.400E-03	8.561E-02	***	4.000E-03
Age 80-84	-4.607E-02	***	4.629E-03	8.467E-02	***	4.208E-03
Age 85-89	-6.786E-02	***	5.558E-03	7.706E-02	***	5.099E-03
Age 90-94	-6.984E-02	***	1.027E-02	7.987E-02	***	1.066E-02
Age >=95	-1.072E-01	**	4.130E-02	9.356E-02		4.858E-02
Female sex	-3.099E-02	***	1.691E-03	-	-	-
Constant term	4.451E-01	***	1.297E-02	1.741E-01	***	1.105E-02
σ (Tobit term)	3.442E-01	-	8.904E-04	2.997E-01	-	7.109E-04
N	208,345			223,836		
Data	PROMs-HES			PROMs-HES		
Candidate models						
Model class	OLS, Tobit, two-part (Logit-OLS)			OLS, Tobit, two-part (Logit-OLS)		
OHS/OKS trend	Quadratic, cubic, quartic, quintic, log-linear			Quadratic, cubic, quartic, quintic, log-linear		
Age trend	Linear, quadratic, cubic, quartic, quintic, five-year bands, exclusion			Linear, quadratic, cubic, quartic, quintic, linear spline (65), five-year bands, exclusion		
Sex	yes/no			yes/no		

For peer review only

Table C.2: Associations between pre-operative OHS/OKS and EQ-5D-3L before TJR

	Hip arthroplasty			Knee arthroplasty		
Model	*Tobit model censored at -0.594 and 1*			*Tobit model censored at -0.594 and 1*		
**Variable**	**Coefficient**		**Robust S.E.**	**Coefficient**		**Robust S.E.**
			OHS/OKS			
Linear term	5.719E-03	**	2.040E-03	1.001E-02	***	2.403E-03
Quadratic term	2.385E-03	***	2.641E-04	2.150E-03	***	2.997E-04
Cubic term	-3.800E-05	*	1.500E-05	-3.140E-05		1.660E-05
Quartic term	-1.480E-06	***	3.850E-07	-1.650E-06	***	4.190E-07
Quintic term	3.010E-08	***	3.650E-09	3.240E-08	***	3.920E-09
			Age			
Linear term	-8.742E-03	***	3.866E-03	-2.943E-02	**	1.046E-02
Quadratic term	3.742E-04		1.062E-04	7.750E-04	**	2.541E-04
Cubic term	-5.190E-06	***	1.240E-06	-8.360E-06	**	2.700E-06
Quartic term	2.340E-08		5.290E-09	3.270E-08	**	1.060E-08
Female sex	-	-	-	1.040E-02	***	8.351E-04
Constant term	-1.649E-01	**	5.079E-02	1.791E-01		1.592E-01
σ (Tobit term)	2.128E-01	-	3.022E-04	2.177E-01	-	2.895E-04
N	271,045			290,983		
Data	PROMs-HES			PROMs-HES		
			Candidate models			
Model class	OLS, Tobit, two-part (Logit-OLS)			OLS, Tobit, two-part (Logit-OLS)		
OHS/OKS trend	Quadratic, cubic, quartic, quintic, log-linear			Quadratic, cubic, quartic, quintic, log-linear		
Age trend	Linear, quadratic, cubic, quartic, quintic, five-year bands, exclusion			Linear, quadratic, cubic, quartic, quintic, five-year bands, exclusion		
Sex	yes/no			yes/no		

Sources: NHS PROMs and HES data, 2009-2015; own calculations.* p<0.05; ** p<0.01; *** p<0.001. Details on how to calculate predictions are provided in Appendix D.

For peer review only

Table C.3: Associations between pre-operative OKS and EQ-5D-3L between 1 and 12 years after TKR

	Knee arthroplasty		
Model	*OLS*		
**Variable**	**Coefficient**		**Clustered S.E.**
	OKS		
Log-linear term	1.776E-01	***	1.111E-02
	Current age		
Linear term	7.168E-03	***	1.541E-03
Linear spline term (70)	-8.851E-03	**	2.551E-03
Year since operation	-6.154E-03	***	1.417E-03
Interaction between year and linear spline term	-3.729E-04	*	1.669E-04
Previously revised	-1.598E-01	***	3.239E-02
Constant term	-2.372E-01	*	1.031E-01
N	15,414 (2,004 patients)		
Data	KAT		
	Candidate models		
Model class	OLS with clustered standard errors, linear mixed models with random intercept and fixed slopes		
Time trend	Linear, quadratic		
OKS trend	Linear, quadratic, cubic, log-linear		
Age trend	For both age at operation and current age: Linear, quadratic, cubic, linear spline (70), exclusion		
Sex	yes/no		

Sources: KAT data; own calculations.* p<0.05; ** p<0.01; *** p<0.001. Details on how to calculate predictions are provided in Appendix D. The model was estimated on data in long-format (i.e., one observation per patient and year). The inclusion of the time trend was tested after selecting the model class and before choosing a trend for OKS. The linear spline term equals "(current age - 70)" for patients above 70, and "0" for those below. The interaction term is derived by multiplying the linear spline term with the year variable. We did not estimate a model for hips due to a lack of data.

For peer review only

Table C.4: Associations between pre-operative OHS/OKS and EQ-5D-3L before revision surgery

	Hip arthroplasty			Knee arthroplasty		
Model	*Tobit model censored at -0.594 and 1*			*Tobit model censored at -0.594 and 1*		
**Variable**	**Coefficient**		**Clustered S.E.**	**Coefficient**		**Clustered S.E.**
OHS/OKS						
Linear term	2.665E-02	***	4.188E-03	2.631E-02	**	8.271E-03
Quadratic term	-2.867E-04	**	1.041E-04	2.356E-04		4.623E-04
Cubic term	-	-	-	-1.510E-05		7.740E-06
Age at primary operation						
Linear term	-	-	-	4.472E-03	***	9.990E-04
Linear spline term (70)	-	-	-	-5.607E-03		2.879E-03
Age at revision surgery						
Linear term	2.123E-03	**	7.587E-04	-	-	-
Logarithm of year of revision	-	-	-	-3.601E-02		1.882E-02
Male sex	-	-	-	-2.358E-02		1.368E-02
Constant term	-1.444E-01	*	6.087E-02	-3.498E-01	***	7.534E-02
σ (Tobit term)	3.178E-01	-	4.755E-03	2.999E-01	-	3.676E-03
N	1,391 (1,331 patients)			2,227 (2,073 patients)		
Data	PROMs-HES			PROMs-HES		
Candidate models						
Model class	OLS, Tobit			OLS, Tobit		
Year of revision (time trend)	Linear, exclusion			Linear, quadratic, cubic, log-linear, exclusion		
OHS/OKS trend	Linear, quadratic, cubic, log-linear			Linear, quadratic, cubic, quartic, log-linear, linear spline (30)		
Age trend	Age at operation vs. age at revision surgery; for age at revision surgery: linear, quadratic, cubic, exclusion			Age at operation vs. age at revision surgery; for age at operation: linear, quadratic, cubic, linear spline (70), exclusion		
Sex	yes/no			yes/no		

Sources: NHS PROMs and HES data, 2009-2015; own calculations.* $p<0.05$; ** $p<0.01$; *** $p<0.001$. Details on how to calculate predictions are provided in Appendix D. The linear spline term equals "(age at operation - 70)" for patients above 70, and "0" for those below.

For peer review only

Table C.5: Associations between pre-operative OHS/OKS and EQ-5D-3L after revision surgery

	Hip arthroplasty			Knee arthroplasty		
Model	*Tobit model censored at -0.594 and 1*			*Tobit model censored at -0.594 and 1*		
**Variable**	**Coefficient**		**Clustered S.E.**	**Coefficient**		**Clustered S.E.**
OHS/OKS						
Log-linear term	2.201E-01	***	2.580E-02	2.556E-01	***	1.950E-02
Age at revision surgery						
Linear term	-8.181E-02		6.954E-02	1.352E-02	***	2.570E-03
Quadratic term	1.755E-03		1.099E-03	-	-	-
Cubic term	-1.120E-05		5.680E-06	-	-	-
Linear spline term (65)	-	-	-	-1.533E-02	***	3.747E-03
Male sex	-5.907E-02	*	2.696E-02	-8.874E-02	***	1.982E-02
Constant term	1.120E+00		1.445E+00	-9.426E-01	***	1.623E-01
σ (Tobit term)	3.633E-01	-	1.184E-02	3.441E-01	-	7.576E-03
N	884 (860 patients)			1,398 (1,331 patients)		
Data	PROMs-HES			PROMs-HES		
Candidate models						
Model class	OLS, Tobit, two-part (Logit-OLS)			OLS, Tobit, two-part (Logit-OLS)		
Year of revision (time trend)	Linear, exclusion			Linear, quadratic, cubic, log-linear, exclusion		
OHS/OKS trend	Linear, quadratic, cubic, log-linear			Linear, quadratic, cubic, quartic, log-linear		
Age trend	Age at operation vs. age at revision surgery; for age at revision surgery: linear, quadratic, cubic, exclusion			Age at operation vs. age at revision surgery; for age at revision surgery: linear, quadratic, cubic, linear spline (65), exclusion		
Sex	yes/no			yes/no		

Sources: NHS PROMs and HES data, 2009-2015; own calculations.* p<0.05; ** p<0.01; *** p<0.001. Details on how to calculate predictions are provided in Appendix D. The linear spline term equals "(age at revision surgery - 65)" for patients above 65, and "0" for those below.

For peer review only

Table C.6: Associations between pre-operative OHS/OKS and the costs of primary arthroplasty

	Hip arthroplasty			Knee arthroplasty		
Model	*gamma-GLM with inverse link function*			*gamma-GLM with inverse link function*		
**Variable**	**Coefficient**		**S.E.**	**Coefficient**		**SE**
OHS/OKS						
Linear term	3.100E-06	***	1.970E-07	2.670E-06	***	2.920E-07
Quadratic term	-2.580E-07	***	2.500E-08	-2.260E-07	***	3.580E-08
Cubic term	1.070E-08	***	1.400E-09	9.700E-09	***	1.960E-09
Quartic term	-2.080E-10	***	3.500E-11	-2.020E-10	***	4.870E-11
Quintic term	1.500E-12	***	3.220E-13	1.600E-12	***	4.470E-13
Age						
Linear term	-8.280E-07		1.220E-06	7.640E-07	***	6.090E-08
Quadratic term	4.310E-08		4.730E-08	-7.760E-09	***	4.460E-10
Cubic term	-8.870E-10		8.730E-10	-	-	-
Quartic term	8.530E-12		7.740E-12	-	-	-
Quintic term	-3.510E-14		2.650E-14	-	-	-
Female sex	7.440E-07	***	9.180E-08	-2.470E-07	*	1.110E-07
Constant term	1.717E-04	***	1.190E-05	1.377E-04	***	2.210E-06
N	286,507			308,638		
Data	PROMs-HES			PROMs-HES		
Candidate models						
Model class	OLS, gamma-GLM with inverse link, gamma-GLM with log-link, Gaussian-GLM with log-link			OLS, gamma-GLM with inverse link, gamma-GLM with log-link, Gaussian-GLM with log-link		
OHS/OKS trend	Linear, cubic, quartic, quintic, sextic, log-linear			Linear, cubic, quartic, quintic, sextic, log-linear		
Age trend	Linear, quadratic, cubic, quartic, quintic, five-year bands, exclusion			Linear, quadratic, cubic, five-year bands, ten-year bands, exclusion		
Sex	yes/no			yes/no		

Sources: NHS PROMs and HES data, 2009-2015; own calculations.* p<0.05; ** p<0.01; *** p<0.001. Details on how to calculate predictions are provided in Appendix D.

For peer review only

Table C.7: Associations between pre-operative OHS/OKS and the costs of revision arthroplasty

	Hip arthroplasty			Knee arthroplasty		
Model	*gamma-GLM with inverse link function*			*OLS*		
**Variable**	**Coefficient**		**Clustered S.E.**	**Coefficient**		**Clustered S.E.**
			OHS/OKS			
Linear term	-2.370E-07	*	9.790E-08	-1.404E+00		6.816E+00
			Age			
Linear term	9.060E-07	*	3.500E-07	-2.101E+01	***	4.846E+00
Quadratic term	-6.620E-09	*	3.010E-09	-	-	-
Female sex	6.700E-06	***	1.570E-06	7.458E+01		9.551E+01
Constant term	9.570E-05	***	1.000E-05	9.111E+03	***	3.275E+02
N	2,359			3,416		
Data	PROMs-HES			PROMs-HES		
			Candidate models			
Model class	OLS, gamma-GLM with inverse link, gamma-GLM with log-link, Gaussian-GLM with log-link			OLS, gamma-GLM with inverse link, gamma-GLM with log-link, Gaussian-GLM with log-link		
OHS/OKS trend	Linear, cubic, log-linear			Linear, cubic, quartic, log-linear		
Age trend	Linear, quadratic, cubic, exclusion			Linear, quadratic, cubic, exclusion		
Sex	yes/no			yes/no		

Sources: NHS PROMs and HES data, 2009-2015; own calculations.* p<0.05; ** p<0.01; *** p<0.001. Details on how to calculate predictions are provided in Appendix D.

For peer review only

Table C.8: Associations between pre-operative OHS/OKS and costs in the 12 months leading up to TJR

	**Hip arthroplasty**			**Knee arthroplasty**		
Model	*gamma-GLM with log-link*			*gamma-GLM with log-link*		
**Variable**	**Coefficient**		**S.E.**	**Coefficient**		**S.E.**
	OHS/OKS					
Log-linear term	-3.485E-01	**	1.177E-01	-	-	-
Linear term	-	-	-	4.307E-02		7.466E-02
Quadratic term	-	-	-	-1.580E-03		1.741E-03
	Age					
Linear term	-8.699E-02		4.441E-02	-2.131E-02		1.430E-02
Quadratic term	5.482E-04		3.489E-04	-	-	-
Female sex	-	-	-	-	-	-
Constant term	1.033E+01	***	1.429E+00	8.027E+00	***	1.302E+00
N	441			278		
Data	COASt			COASt		
	Candidate models					
Model class	OLS, Tobit censored at zero, gamma-GLM with log-link, two-part (Logit + OLS/gamma-GLM with log-link/gamma-GLM with inverse link)			OLS, Tobit censored at zero, gamma-GLM with log-link, two-part (Logit + OLS/gamma-GLM with log-link/gamma-GLM with inverse link)		
OHS/OKS trend	Linear, quadratic, cubic, log-linear			Linear, quadratic, cubic		
Age trend	Linear, quadratic, cubic, 20-year bands, exclusion			Linear, quadratic, cubic, 20-year bands, exclusion		
Sex	yes/no			yes/no		

Sources: COASt data; own calculations.* p<0.05; ** p<0.01; *** p<0.001. Details on how to calculate predictions are provided in Appendix D. For observations with OHS=0, we set ln(OHS)=0.

For peer review only

Table C.9: Associations between pre-operative OHS/OKS and ambulatory costs within 12 months after primary arthroplasty

	Hip arthroplasty			Knee arthroplasty		
Model	*OLS Regression*			*gamma-GLM with log-link*		
**Variable**	**Coefficient**		**Robust S.E.**	**Coefficient**		**Robust S.E.**
OHS/OKS						
Linear term	2.881E+01	**	8.642E+00	-1.552E-02	***	3.408E-03
Quadratic term	-1.403E+00	**	4.400E-01	-	-	-
Cubic term	1.827E-02	**	6.815E-03	-	-	-
Age						
Linear term	-2.670E+00	*	1.128E+00	-2.908E-02	***	3.060E-03
Female sex	-	-	-	-	-	-
Constant term	1.371E+02		9.342E+01	8.170E+00	***	2.203E-01
N	548			1,841		
Data	COASt			KAT		
Candidate models						
Model class	OLS, Tobit censored at zero, gamma-GLM with inverse link, gamma-GLM with log-link, Gaussian-GLM with log-link, two-part (Logit + OLS/gamma-GLM with inverse link/gamma-GLM with log-link/Gaussian-GLM with log-link)			OLS, Tobit censored at zero, gamma-GLM with inverse link, gamma-GLM with log-link, Gaussian-GLM with log-link, two-part (Logit + OLS/gamma-GLM with inverse link/gamma-GLM with log-link/Gaussian-GLM with log-link)		
OHS/OKS trend	Linear, quadratic, cubic, linear spline (20)			Linear, cubic, linear spline (25)		
Age trend	Linear, quadratic, cubic, exclusion			Linear, quadratic, cubic, log-linear, exclusion		
Sex	yes/no			yes/no		

Sources: COASt and KAT data; own calculations.* p<0.05; ** p<0.01; *** p<0.001. Details on how to calculate predictions are provided in Appendix D.

For peer review only

Table C.10: Associations between pre-operative OHS/OKS and the costs of hospital readmissions within 12 months after primary arthroplasty

	Hip arthroplasty			Knee arthroplasty		
Model	*Gaussian-GLM with log-link function*			*Gaussian-GLM with log-link function*		
**Variable**	**Coefficient**		**Clustered S.E.**	**Coefficient**		**Clustered S.E.**
OHS/OKS						
Linear term	-1.727E-01	***	3.719E-02	-1.177E-01	***	1.342E-02
Quadratic term	1.325E-02	**	5.060E-03	3.638E-03	***	7.422E-04
Cubic term	-6.122E-04	*	2.955E-04	-4.350E-05	***	1.230E-05
Quartic term	1.370E-05		7.640E-06	-	-	-
Quintic term	-1.140E-07		7.160E-08	-	-	-
Age						
Linear term	5.318E-03	**	1.671E-03	-8.114E-02	***	9.237E-03
Linear spline (age ≥70)	3.448E-02	***	3.360E-03	-	-	-
Quadratic term	-	-	-	7.103E-04	***	6.670E-05
Female sex	-1.785E-01	***	2.144E-02	-2.500E-01	***	2.070E-02
Constant term	6.788E+00	***	1.476E-01	9.687E+00	***	3.008E-01
N	236,514			255,194		
Data	PROMs-HES			PROMs-HES		
Candidate models						
Model class	OLS, Tobit censored at zero, two-part (Logit + OLS/gamma-GLM with inverse link/gamma-GLM with log-link/Gaussian-GLM with log-link), gamma-GLM with inverse link, gamma-GLM with log-link, Gaussian-GLM with log-link			OLS, Tobit censored at zero, two-part (Logit + OLS/gamma-GLM with inverse link/gamma-GLM with log-link/Gaussian-GLM with log-link), gamma-GLM with inverse link, gamma-GLM with log-link, Gaussian-GLM with log-link		
OHS/OKS trend	Linear spline (10), quadratic, cubic, quartic, quintic			Linear spline (10), quadratic, cubic, quartic, quintic		
Age trend	Linear spline (70), linear, quadratic, cubic, exclusion			Linear spline (70), linear, quadratic, cubic, exclusion		
Sex	yes/no			yes/no		

Sources: NHS PROMs and HES data, 2009-2015; own calculations.* p<0.05; ** p<0.01; *** p<0.001. Details on how to calculate predictions are provided in Appendix D. The linear spline term is set to zero for patients aged below 70. For patients above 70, the term equals (age in years -70).

For peer review only

Table C.11: Associations between pre-operative OHS/OKS and annual costs of hospital readmissions between 1 and 6 years after primary arthroplasty

	Hip arthroplasty			Knee arthroplasty		
Model	*gamma-GLM with inverse link function*			*gamma-GLM with inverse link function*		
**Variable**	**Coefficient**		**Clustered S.E.**	**Coefficient**		**Clustered S.E.**
OHS/OKS						
Linear term	1.998E-04	***	1.660E-05	1.655E-04	***	8.150E-06
Current age						
Linear term	4.230E-05	***	9.000E-06	-	-	-
Age at primary arthroplasty						
Linear term	-	-	-	4.600E-05	***	5.300E-06
Time trend						
Linear term	-	-	-	1.227E-03	***	6.870E-05
Year 2 since primary surgery	-2.446E-03	***	2.263E-04	-	-	-
Female sex	-	-	-	4.290E-04	***	1.138E-04
Constant term	3.432E-03	***	7.112E-04	-3.965E-03	***	3.662E-04
N	476,514			514,047		
Data	PROMs-HES			PROMs-HES		
Candidate models						
Model class	OLS, Tobit censored at zero, two-part (Logit + OLS/gamma-GLM with inverse link/gamma-GLM with log-link/Gaussian-GLM with log-link), gamma-GLM with inverse link, gamma-GLM with log-link, Gaussian-GLM with log-link			OLS, Tobit censored at zero, two-part (Logit + OLS/gamma-GLM with inverse link/gamma-GLM with log-link/Gaussian-GLM with log-link), gamma-GLM with inverse link, gamma-GLM with log-link, Gaussian-GLM with log-link		
Year since primary surgery (time trend)	Linear, quadratic, binary indicator for year 2, binary indicators for all years, exclusion			Linear, quadratic, binary indicator for year 2, binary indicators for all years, exclusion		
OHS/OKS trend	Linear, quadratic, cubic, quartic, quintic, log-linear			Linear, quadratic, cubic, quartic, quintic, log-linear		
Age trend	Age at operation vs. current age; for current age: Linear, quadratic, cubic, five-year age bands, exclusion			Age at operation vs. current age; for age at operation: Linear, quadratic, cubic, quartic, quintic, five-year age bands, exclusion		
Sex	yes/no			yes/no		

Sources: NHS PROMs and HES data, 2009-2015; own calculations.* p<0.05; ** p<0.01; *** p<0.001. Details on how to calculate predictions are provided in Appendix D. "Year 2 since primary surgery" is a binary indicator. "Year 1" refers to the first 12 months after primary surgery.

For peer review only

Table C.12: Associations between pre-operative OHS/OKS and the costs of hospital readmissions during the year of revision surgery

	Hip arthroplasty			Knee arthroplasty		
Model	*Two-part model: Logit + gamma-GLM with inverse link*			*Two-part model: Logit + gamma-GLM with log-link*		
**Variable**	**Coefficient**		**Clustered S.E.**	**Coefficient**		**Clustered S.E.**
	**Part 1: Logit model**					
	OHS/OKS					
Linear term	8.407E-03		1.093E-02	3.790E-03		8.559E-03
	Age at primary arthroplasty					
Linear term	9.589E-02	*	4.418E-02	5.140E-04		6.716E-03
Quadratic term	-8.234E-04	*	3.622E-04	-	-	-
	Time trend					
Linear term	-	-	-	-2.488E+00		1.438E+00
Quadratic term	-	-	-	6.157E-01		3.933E-01
Cubic term	-	-	-	-4.586E-02		3.340E-02
Year 2 since primary surgery	3.153E-01		1.675E-01	-	-	-
Female sex	1.724E-01		1.624E-01	2.386E-01		1.235E-01
Constant term	-8.202E-01		1.353E+00	4.622E+00	**	1.699E+00
	**Part 2: gamma-GLM**					
	OHS/OKS					
Linear term	-1.340E-06		9.040E-07	-1.291E-02		7.915E-03
	Age at primary arthroplasty					
Linear term	-9.550E-07		5.400E-06	1.018E-02		5.999E-03
Quadratic term	-4.800E-09		4.080E-08	-	-	-
	Time trend					
Linear term	-	-	-	-3.169E-01		1.725E+00
Quadratic term	-	-	-	1.474E-01		4.819E-01
Cubic term	-	-	-	-1.556E-02		4.192E-02
Year 2 since primary surgery	4.800E-05	**	1.760E-05	-	-	-
Female sex	5.210E-05	*	2.040E-05	-9.075E-02		1.228E-01
Constant term	2.078E-04		1.764E-04	8.352E+00	***	1.982E+00
N	1,669			2,258		
Data	PROMs-HES			PROMs-HES		

...continued on next page

For peer review only

	Candidate models	
Model class	OLS, Tobit censored at zero, two-part (Logit + OLS/gamma-GLM with inverse link/gamma-GLM with log-link/Gaussian-GLM with log-link), gamma-GLM with inverse link, gamma-GLM with log-link, Gaussian-GLM with log-link	OLS, Tobit censored at zero, two-part (Logit + OLS/gamma-GLM with inverse link/gamma-GLM with log-link/Gaussian-GLM with log-link), gamma-GLM with inverse link, gamma-GLM with log-link, Gaussian-GLM with log-link
Year since primary surgery (time trend)	Binary indicator for year 2, exclusion	Linear, quadratic, cubic, exclusion
OHS/OKS trend	Linear, quadratic, cubic	Linear, quadratic, cubic
Age trend	Age at operation vs. current age; for age at operation: Linear, quadratic, cubic, five-year age bands, exclusion	Age at operation vs. current age; for age at operation: Linear, quadratic, cubic, five-year age bands, exclusion
Sex	yes/no	yes/no

Sources: NHS PROMs and HES data, 2009-2015; own calculations.* p<0.05; ** p<0.01; *** p<0.001. Details on how to calculate predictions are provided in Appendix D. "Year 2 since primary surgery" is a binary indicator. "Year 1" refers to the first 12 months after primary surgery.

For peer review only

Table C.13: Associations between pre-operative OHS/OKS and annual costs of hospital readmissions for patient with a previous revision

	Hip arthroplasty			Knee arthroplasty		
Model	*Tobit model censored at zero*			*Tobit model censored at zero*		
**Variable**	**Coefficient**		**Clustered S.E.**	**Coefficient**		**Clustered S.E.**
OHS/OKS						
Linear term	-2.424E+02	*	1.074E+02	-1.972E+02	*	9.961E+01
Current age						
Linear term	-	-	-	-1.172E+04	*	4.527E+03
Quadratic term	-	-	-	1.772E+02	*	7.058E+01
Cubic term	-	-	-	-8.911E-01	*	3.609E-01
Time trend						
Linear term	-	-	-	-1.935E+03	***	4.973E+02
Constant term	-2.454E+04	***	3.657E+03	2.455E+05	**	9.386E+04
σ (Tobit term)	1.472E+04	-	1.859E+03	1.390E+04		2.139E+03
N	2,406			3,153		
Data	PROMs-HES			PROMs-HES		
Candidate models						
Model class	OLS, Tobit censored at zero, two-part (Logit + OLS/gamma-GLM with inverse link/gamma-GLM with log-link/Gaussian-GLM with log-link), gamma-GLM with inverse link, gamma-GLM with log-link, Gaussian-GLM with log-link			OLS, Tobit censored at zero, two-part (Logit + OLS/gamma-GLM with inverse link/gamma-GLM with log-link/Gaussian-GLM with log-link), gamma-GLM with inverse link, gamma-GLM with log-link, Gaussian-GLM with log-link		
Year since primary surgery (time trend)	Linear, quadratic, exclusion			Linear, quadratic, cubic, exclusion		
OHS/OKS trend	Linear, quadratic, cubic			Linear, quadratic, cubic, quartic, quintic, log-linear		
Age trend	Age at operation vs. current age; for age at operation: linear, quadratic, cubic, five-year age bands, exclusion			Age at operation vs. current age; forcurrent age: linear, quadratic, cubic, exclusion		
Sex	yes/no			yes/no		

Sources: NHS PROMs and HES data, 2009-2015; own calculations.* p<0.05; ** p<0.01; *** p<0.001. Details on how to calculate predictions are provided in Appendix D.

For peer review only

Table C.14: Associations between pre-operative OKS and annual ambulatory costs between 1 and 12 years after TKR

	Knee arthroplasty		
Model	*Gaussian-GLM with log-link function*		
**Variable**	**Coefficient**		**Clustered S.E.**
	OKS		
Linear term	1.125E-03		9.113E-04
Quadratic term	-5.597E-01	*	2.420E-01
	Age at primary operation		
Linear term	3.672E-01		2.467E-01
Quadratic term	-7.167E-03		4.043E-03
Cubic term	4.130E-05		2.150E-05
	Year since primary surgery (time trend)		
Linear term	-5.597E-01	*	2.420E-01
Quadratic term	5.295E-02		4.104E-02
Cubic term	-1.810E-03		2.111E-03
Female sex	-3.197E-01	*	1.363E-01
Constant term	1.212E+00		4.801E+00
N	13,271		
Data	KAT		
	Candidate models		
Model class	OLS, Tobit censored at zero, two-part (Logit + OLS/gamma-GLM with log-link), gamma-GLM with inverse link, gamma-GLM with log-link, Gaussian-GLM with log-link		
Time trend	Linear, quadratic, cubic trend, binary indicator for year 2, binary indicator for year 2 and a linear trend, exclusion		
OKS trend	Linear, quadratic, cubic		
Age trend	Age at operation vs. current age; for age at operation: Linear, quadratic, cubic, exclusion		
Sex	yes/no		

Sources: KAT data; own calculations.* p<0.05; ** p<0.01; *** p<0.001. Details on how to calculate predictions are provided in Appendix D. The model was estimated on data in long-format (i.e., one observation per patient and year). The inclusion of the time trend was tested after selecting the model class and before choosing a trend for OKS. We did not estimate a model for hips due to a lack of data.

For peer review only

Table C.15: Associations between pre-operative OKS and ambulatory costs in the year of revision surgery

	Knee arthroplasty		
Model	*gamma-GLM with log-link function*		
**Variable**	**Coefficient**		**Clustered S.E.**
	OKS		
Linear term	-3.271E-02		1.805E-02
	Age at primary operation		
Linear term	2.062E-01	*	8.868E-02
Quadratic term	-1.777E-03	**	6.774E-04
Constant term	1.145E+00		2.861E+00
N	88		
Data	KAT		
	Candidate models		
Model class	OLS, Tobit censored at zero, two-part (Logit + OLS/gamma-GLM with log-link/gamma-GLM with inverse link/Gaussian-GLM with log-link), gamma-GLM with inverse link, gamma-GLM with log-link, Gaussian-GLM with log-link		
OKS trend	Linear, quadratic, cubic		
Age trend	Linear, quadratic, cubic, exclusion		
Sex	yes/no		

Sources: KAT data; own calculations.* p<0.05; ** p<0.01; *** p<0.001. Details on how to calculate predictions are provided in Appendix D. The model was estimated on data in long-format (i.e., one observation per patient and year). We did not estimate a model for hips due to a lack of data.

For peer review only

Table C.16: Associations between pre-operative OKS and annual ambulatory costs for patients with a previous revision

	Knee arthroplasty		
Model	*gamma-GLM with inverse link function*		
**Variable**	**Coefficient**		**Clustered S.E.**
	OKS		
Linear term	1.637E-03		9.185E-04
Quadratic term	-1.439E-04		7.930E-05
Cubic term	3.860E-06	*	1.890E-06
Female sex	1.726E-03		2.068E-03
Constant term	3.737E-04		1.859E-03
N	329		
Data	KAT		
	Candidate models		
Model class	OLS, Tobit censored at zero, two-part (Logit + OLS/gamma-GLM with log-link/gamma-GLM with inverse link/Gaussian-GLM with log-link), gamma-GLM with inverse link, gamma-GLM with log-link, Gaussian-GLM with log-link		
OKS trend	Linear, quadratic, cubic		
Age trend	Linear, quadratic, cubic, exclusion		
Sex	yes/no		

Sources: KAT data; own calculations.* p<0.05; ** p<0.01; *** p<0.001. Details on how to calculate predictions are provided in Appendix D. Notes: The model was estimated on data in long-format (i.e., one observation per patient and year). We did not estimate a model for hips due to a lack of data.

For peer review only

D. Note on calculating predictions

1) OLS models

The predicted value of the outcome variable, $\hat{y}$ is simply the linear predictor Xb, which is obtained by multiplying each variable with its estimated coefficient and then summing over all variables.

Example:

For a patient aged 60 and with an OHS of 18, the ambulatory costs in the first 12 months after primary THR (Table C.9) can be predicted as:

$$\begin{aligned} \cos t &= 28.81 \times OHS - 1.40 \times OHS^2 + 0.02 \times OHS^3 - 2.67 \times age + 137.10 \\ &= 28.81 \times 18 - 1.40 \times 324 + 0.02 \times 5832 - 2.67 \times 60 + 137.10 \\ &= 147.46 \end{aligned}$$

2) Gamma-GLM with an inverse link function

Predicted values for the outcome variable can be obtained as $\hat{y} = \dfrac{1}{Xb}$.

Example:

For a female patient with OKS of 15, the annual ambulatory costs after revision TKR surgery (Table C.16) can be calculated as follows:

$$\begin{aligned} \cos t &= \frac{1}{0.000374 + 0.001726 \times female + 0.001637 \times OKS - 0.000144 \times OKS^2 + 0.000004 \times OKS^3} \\ &= \frac{1}{0.000374 + 0.001726 \times 1 + 0.001637 \times 15 - 0.000144 \times 225 + 0.000004 \times 3375^3} \\ &= \frac{1}{0.007305} \\ &= 136.90 \end{aligned}$$

3) Gamma-GLM with a log-link function

Predicted values for the outcome variable can be obtained as $\hat{y} = e^{Xb}$.

For peer review only

Example: For a patient aged 60 at the time of her primary TKR operation and with an OKS of 10, the ambulatory cost in the year of revision surgery (Table C.15) can be calculated as follows:

$$\begin{aligned}\cos t &= e^{1.145-0.033\times OKS+0.206\times age-0.002\times age^2}\\ &= e^{1.145-0.033\times 10+0.206\times 60-0.002\times 3600}\\ &= e^{6.7927}\\ &= 2.7182^{6.7927}\\ &= 891.32\end{aligned}$$

4) Gaussian-GLM with a log-link functio

Predicted values for the outcome variable can be obtained as $\hat{y} = e^{Xb}$.

Example: For a female patient aged 70 and with an OKS of 70, the cost of hospital readmission in the first 12 months after primary TKR (Table C.10) can be calculated as follows:

$$\begin{aligned}\cos t &= e^{9.68700-0.25\times female-0.11770\times OKS+0.00364\times OKS^2-0.00004\times OKS^3-0.08114\times age+0.00071\times age^2}\\ &= e^{9.68700-0.25\times 1-0.11770\times 20+0.00364\times 400-0.00004\times 8000-0.08114\times 70+0.00071\times 4900}\\ &= e^{5.99087}\\ &= 2.7182^{5.99087}\\ &= 399.76\end{aligned}$$

5) Tobit models censored at zero

The predicted value is given by $\hat{y} = -\Phi\left(\frac{Xb}{\sigma}\right)Xb + \sigma\phi\left(\frac{Xb}{\sigma}\right)$

where $\Phi(\cdot)$ denotes the cumulative distribution function of the standard normal distribution, and $\phi(\cdot)$ denotes the probability density function of the standard normal distribution.

Example: For a patient with an OHS of 25, the annual cost of hospital readmissions after revision surgery (Table C.13) can be calculated as follows:

For peer review only

$$\cos t = -\Phi\left(\frac{-24540 - 242.40 \times OHS}{14720}\right)(-24540 - 242.40 \times OHS) + 14720\phi\left(\frac{-24540 - 242.40 \times OHS}{14720}\right)$$
$$= -\Phi\left(\frac{-24540 - 242.40 \times 20}{14720}\right)(-24540 - 242.40 \times 20) + 14720\phi\left(\frac{-24540 - 242.40 \times 20}{14720}\right)$$
$$= -\Phi(-1.99647)(-29388) + 14720\phi(-1.99647)$$
$$= 0.022942 \times (-29388) + 14720 \times 0.054373$$
$$= 126.15$$

6) Tobit models censored at -0.594 and 1

The predicted value is given by

$$\hat{y} = -0.594 * \Phi\left(\frac{-0.594 - Xb}{\sigma}\right) + 1 * \Phi\left(-\frac{1 - Xb}{\sigma}\right)$$
$$+\left(\Phi\left(\frac{1 - Xb}{\sigma}\right) - \Phi\left(\frac{-0.594 - Xb}{\sigma}\right)\right) * \left(Xb - \sigma * \frac{\phi\left(\frac{1 - Xb}{\sigma}\right) - \phi\left(\frac{-0.594 - Xb}{\sigma}\right)}{\Phi\left(\frac{1 - Xb}{\sigma}\right) - \Phi\left(\frac{-0.594 - Xb}{\sigma}\right)}\right)$$

where $\Phi(\cdot)$ denotes the cumulative distribution function of the standard normal distribution, and $\phi(\cdot)$ denotes the probability density function of the standard normal distribution.

Example: For a patient aged 75 in the year of THR revision surgery and with a pre-operative (pre-primary) OHS of 8, the EQ-5D utility before revision surgery (Table C.4) can be calculated with the following steps.

First, the linear predictor (*Xb*) is calculated as:

$$Xb = -0.1444 + 0.0021 \times currentage + 0.0267 \times OHS - 0.0003 \times OHS^2$$
$$= -0.1444 + 0.0021 \times 75 + 0.0267 \times 8 - 0.0003 \times 64$$
$$= 0.21$$

Then, using this value and the formula above the predicted EQ-5D utility can be calculated as follows:

$$QoL = -0.594 \times \Phi\left(\frac{-0.594-0.21}{0.3178}\right) + 1 \times \Phi\left(-\frac{1-0.21}{0.3178}\right)$$
$$+\left(\Phi\left(\frac{1-0.21}{0.3178}\right) - \Phi\left(\frac{-0.594-0.21}{0.3178}\right)\right) \times \left(0.21 - 0.3178 \times \frac{\phi\left(\frac{1-0.21}{0.3178}\right) - \phi\left(\frac{-0.594-0.21}{0.3178}\right)}{\Phi\left(\frac{1-0.21}{0.3178}\right) - \Phi\left(\frac{-0.594-0.21}{0.3178}\right)}\right)$$
$$= -0.594 \times 0.005705 + 1 \times 0.006462 + (0.993538 - 0.005705) \times \left(0.21 - 0.3178 \times \frac{0.018158 - 0.016259}{0.993538 - 0.005705}\right)$$
$$= -0.00339 + 0.006462 + 0.987833 \times (0.21 - 0.000611)$$
$$= 0.209913$$

7) Two-part models

The predicted value from a two-part model is given by
$\hat{y} = (1 - P[y=0 \mid X]) * E[y \mid y>0, X]$.

$E[y \mid y>0, X]$ is given by the predicted value in the second part of the model.

$P[y=0 \mid X]$ is given as $P[y=0 \mid X] = \frac{e^{Xb}}{1+e^{Xb}}$ if the first part of the model is a logit model.

Example: For a female patient aged 65 in the year of her primary THR operation and with an OHS of 15, the costs of hospital readmissions in the year of revision surgery in year 3 since her primary operation (Table C.12) can be calculated with the following steps:

First, we calculate the linear predictor, *Xb*, of the first part of the model:

$$Xb_1 = -0.820 + 0.172 \times female + 0.315 \times year2 + 0.008 \times OHS + 0.096 \times age - 0.001 \times age^2$$
$$= -0.820 + 0.172 \times 1 + 0.315 \times 0 + 0.008 \times 15 + 0.096 \times 65 - 0.001 \times 4225$$
$$= 2.23229$$

Then, the probability of zero cost can be calculated as follows:

$$P[\cos t = 0 \mid Xb] = \frac{e^{Xb}}{1+e^{Xb}}$$
$$= \frac{e^{2.23229}}{1+e^{2.23229}}$$
$$= \frac{2.718281^{2.23229}}{1+2.718281^{2.23229}}$$
$$= \frac{9.321187}{1+9.321187}$$
$$= 0.903112$$

Next, the linear predictor of the second part of the model is calculated as:

$$Xb_2 = 0.0002078 + 0.0000521 \times female + 0.000048 \times year2 - 0.00000134 \times OHS$$
$$-0.000000955 \times age - -0.000000005 \times age^2$$
$$= 0.0002078 + 0.0000521 \times 1 + 0.000048 \times 0 - 0.00000134 \times 15$$
$$-0.000000955 \times 65 - -0.000000005 \times 4225$$
$$= 0.000157$$

Then, the predicted costs of the second part of the model can be calculated as:

$$E\left[\cos t \mid \cos t > 0, Xb\right] = \frac{1}{Xb}$$
$$= \frac{1}{0.000157}$$
$$= 6351.424$$

The formula used here is the prediction formula for a gamma-GLM with an inverse link function (see section 2), since this is the model used for the second part in Table C.12. The first part of the model predicts that for the patient described in this example the probability of incurring no cost for hospital readmissions in the year of revision surgery is 90.3%. The second part predicts that if the patient has a hospital admission, than the predicted annual costs for readmissions are £6351.4.

These two results can be combined to derive the predicted cost of hospital readmissions as follows:

$$\cos t = \left(1 - P\left[y = 0 \mid X\right]\right) * E\left[y \mid y > 0, X\right]$$
$$= (1 - 0.903112) * 6351.424$$
$$= 615.38$$

This values takes into account that there is a high probability that the cost are zero.

For peer review only

STROBE 2007 (v4) checklist of items to be included in reports of observational studies in epidemiology*
Checklist for cohort, case-control, and cross-sectional studies (combined)

Section/Topic	Item #	Recommendation	Reported on page #
**Title and abstract**	1	(*a*) Indicate the study's design with a commonly used term in the title or the abstract	p.1/2
		(*b*) Provide in the abstract an informative and balanced summary of what was done and what was found	p.2
**Introduction**			
Background/rationale	2	Explain the scientific background and rationale for the investigation being reported	p.4
Objectives	3	State specific objectives, including any pre-specified hypotheses	p.4
**Methods**			
Study design	4	Present key elements of study design early in the paper	pp. 4-11
Setting	5	Describe the setting, locations, and relevant dates, including periods of recruitment, exposure, follow-up, and data collection	p.4/5
Participants	6	(*a*) *Cohort study*—Give the eligibility criteria, and the sources and methods of selection of participants. Describe methods of follow-up<br>*Case-control study*—Give the eligibility criteria, and the sources and methods of case ascertainment and control selection. Give the rationale for the choice of cases and controls<br>*Cross-sectional study*—Give the eligibility criteria, and the sources and methods of selection of participants	p.5
		(*b*) *Cohort study*—For matched studies, give matching criteria and number of exposed and unexposed<br>*Case-control study*—For matched studies, give matching criteria and the number of controls per case	
Variables	7	Clearly define all outcomes, exposures, predictors, potential confounders, and effect modifiers. Give diagnostic criteria, if applicable	pp.5-8
Data sources/ measurement	8*	For each variable of interest, give sources of data and details of methods of assessment (measurement). Describe comparability of assessment methods if there is more than one group	*pp.5-8*
Bias	9	Describe any efforts to address potential sources of bias	p. 10/11
Study size	10	Explain how the study size was arrived at	NA
Quantitative variables	11	Explain how quantitative variables were handled in the analyses. If applicable, describe which groupings were chosen and why	pp.5-11
Statistical methods	12	(*a*) Describe all statistical methods, including those used to control for confounding	p.10/11
		(*b*) Describe any methods used to examine subgroups and interactions	p.10/11
		(*c*) Explain how missing data were addressed	p.10/11
		(*d*) *Cohort study*—If applicable, explain how loss to follow-up was addressed<br>*Case-control study*—If applicable, explain how matching of cases and controls was addressed	NA

For peer review only

		Cross-sectional study—If applicable, describe analytical methods taking account of sampling strategy	
		(*e*) Describe any sensitivity analyses	p.10/11
**Results**			
Participants	13*	(a) Report numbers of individuals at each stage of study—eg numbers potentially eligible, examined for eligibility, confirmed eligible, included in the study, completing follow-up, and analysed	p.4/5, Table 1
		(b) Give reasons for non-participation at each stage	NA
		(c) Consider use of a flow diagram	NA
Descriptive data	14*	(a) Give characteristics of study participants (eg demographic, clinical, social) and information on exposures and potential confounders	Table 1
		(b) Indicate number of participants with missing data for each variable of interest	Table
		(c) *Cohort study*—Summarise follow-up time (eg, average and total amount)	NA
Outcome data	15*	*Cohort study*—Report numbers of outcome events or summary measures over time	
		Case-control study—Report numbers in each exposure category, or summary measures of exposure	
		Cross-sectional study—Report numbers of outcome events or summary measures	Table 1
Main results	16	(*a*) Give unadjusted estimates and, if applicable, confounder-adjusted estimates and their precision (eg, 95% confidence interval). Make clear which confounders were adjusted for and why they were included	supplementary material
		(*b*) Report category boundaries when continuous variables were categorized	supplementary material
		(*c*) If relevant, consider translating estimates of relative risk into absolute risk for a meaningful time period	NA
Other analyses	17	Report other analyses done—eg analyses of subgroups and interactions, and sensitivity analyses	
**Discussion**			
Key results	18	Summarise key results with reference to study objectives	p. 16
Limitations	19	Discuss limitations of the study, taking into account sources of potential bias or imprecision. Discuss both direction and magnitude of any potential bias	p. 17/18
Interpretation	20	Give a cautious overall interpretation of results considering objectives, limitations, multiplicity of analyses, results from similar studies, and other relevant evidence	p. 16/17
Generalisability	21	Discuss the generalisability (external validity) of the study results	p. 16
**Other information**			
Funding	22	Give the source of funding and the role of the funders for the present study and, if applicable, for the original study on which the present article is based	p. 19/20

*Give information separately for cases and controls in case-control studies and, if applicable, for exposed and unexposed groups in cohort and cross-sectional studies.

For peer review only

Note: An Explanation and Elaboration article discusses each checklist item and gives methodological background and published examples of transparent reporting. The STROBE checklist is best used in conjunction with this article (freely available on the Web sites of PLoS Medicine at http://www.plosmedicine.org/, Annals of Internal Medicine at http://www.annals.org/, and Epidemiology at http://www.epidem.com/). Information on the STROBE Initiative is available at www.strobe-statement.org.

For peer review only
